# Cembranoids of Soft Corals: Recent Updates and Their Biological Activities

**DOI:** 10.1007/s13659-021-00303-2

**Published:** 2021-04-22

**Authors:** Marsya Yonna Nurrachma, Deamon Sakaraga, Ahmad Yogi Nugraha, Siti Irma Rahmawati, Asep Bayu, Linda Sukmarini, Akhirta Atikana, Anggia Prasetyoputri, Fauzia Izzati, Mega Ferdina Warsito, Masteria Yunovilsa Putra

**Affiliations:** Research Center for Biotechnology, Indonesian Institute of Sciences (LIPI), Jalan Raya Jakarta-Bogor KM. 46, Cibinong, Bogor, West Java Indonesia

**Keywords:** Cembranoids, Diterpene, Soft corals, *Sarcophyton*, *Sinularia*, *Lobophytum*, Anti-bacterial, Anti-cancer, Anti-inflammatory

## Abstract

**Abstract:**

Soft corals are well-known as excellent sources of marine-derived natural products. Among them, members of the genera *Sarcophyton*, *Sinularia*, and *Lobophytum* are especially attractive targets for marine natural product research. In this review, we reported the marine-derived natural products called cembranoids isolated from soft corals, including the genera *Sarcophyton*, *Sinularia*, and *Lobophytum*. Here, we reviewed 72 reports published between 2016 and 2020, comprising 360 compounds, of which 260 are new compounds and 100 are previously known compounds with newly recognized activities. The novelty of the organic molecules and their relevant biological activities, delivered by the year of publication, are presented. Among the genera presented in this report, *Sarcophyton* spp. produce the most cembranoid diterpenes; thus, they are considered as the most important soft corals for marine natural product research. Cembranoids display diverse biological activities, including anti-cancer, anti-bacterial, and anti-inflammatory. As cembranoids have been credited with a broad range of biological activities, they present a huge potential for the development of various drugs with potential health and ecological benefits.

**Graphic Abstract:**

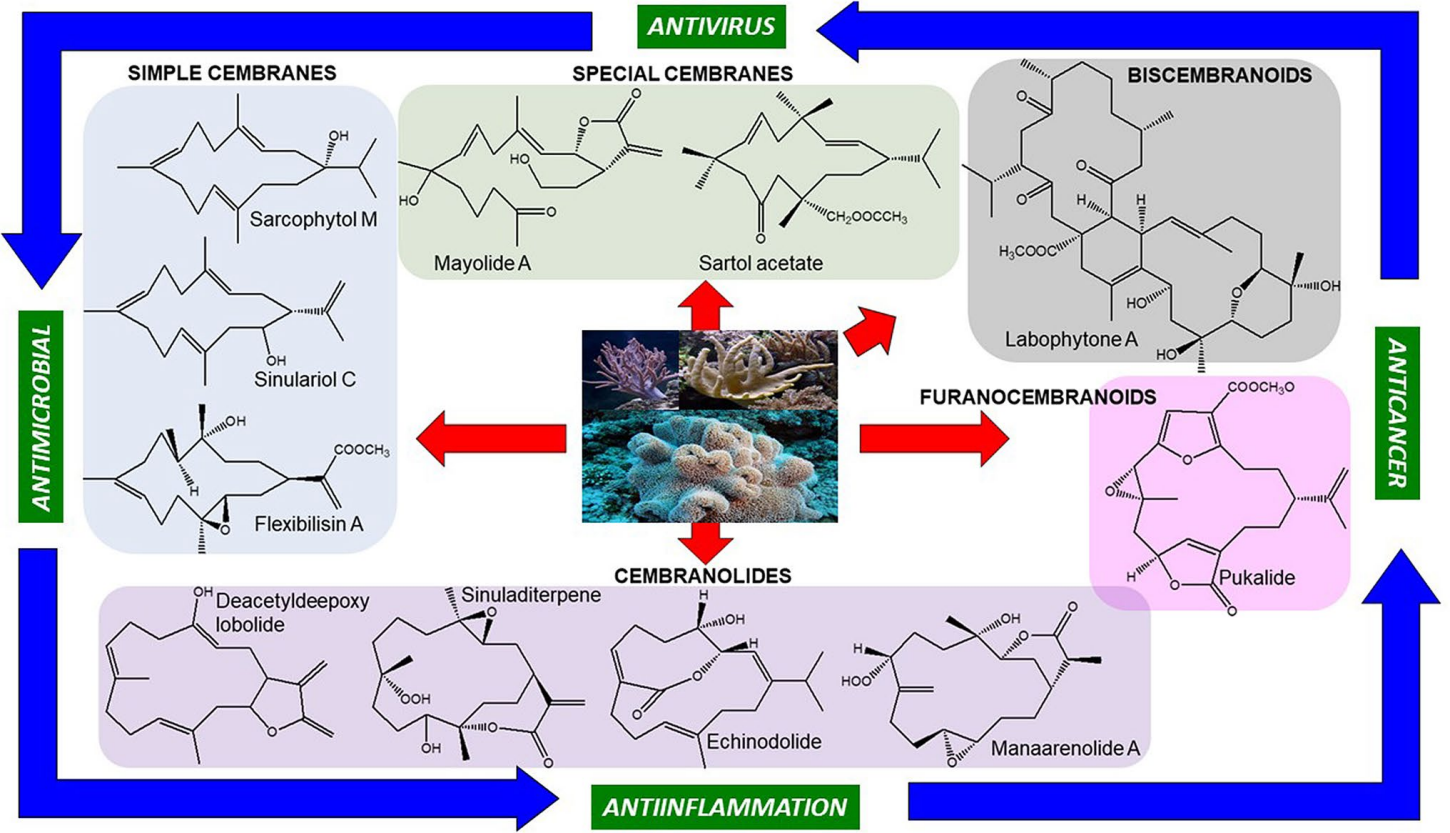

## Introduction

The ocean represents the largest habitat on earth, covering over 70% of the earth surface and harboring a large number of marine organisms whose living environments are quite different from those of their land-based counterparts [1–3]. The extreme ocean conditions, *e.g.* high pressure, high salinity, hypoxia, and low light levels [4], lead marine organisms to synthesize the highly diverse and unique biological and chemical entities. As a result, the ocean is an important source of natural products with remarkable bioactivities for (novel) drug discovery. Among marine organisms, sessile animals such as soft corals have been shown to have strong chemical defense systems, which are reflected in the almost infinite structural diversity and complexity of their secondary metabolites. Hence, these organisms have long attracted the interest of natural product chemists for drug discovery research and development [5].

Soft corals (phylum, Cnidaria; class, Anthozoa; subclass, Octocorallia; order, Alcyonaceae; family, Alcyoniidae) have been studied as sources of marine-derived natural products since the nineteenth century [6]. They are generally found in Indo Pacific reefs, whereas Gorgonian octocorals dominate the biomass in coral reef environments of the north-western Atlantic Ocean and the Caribbean Sea [7]. The subclass Octocorallia including soft corals, gorgonians, and sea pens, are the most commonly studied corals for drug discovery [8]. The main natural product isolated from soft corals is cembranoids, which act as chemical defense compounds against fish predators. Generally, these metabolites are obtained from the genera *Sarcophyton*, *Sinularia*, *Lobophytum*, *Eunicea*, and *Clavularia* [7, 9–11]. Among all, the first three genera attract the most interest in the study of cembranoids [6].

**Fig. 1:**
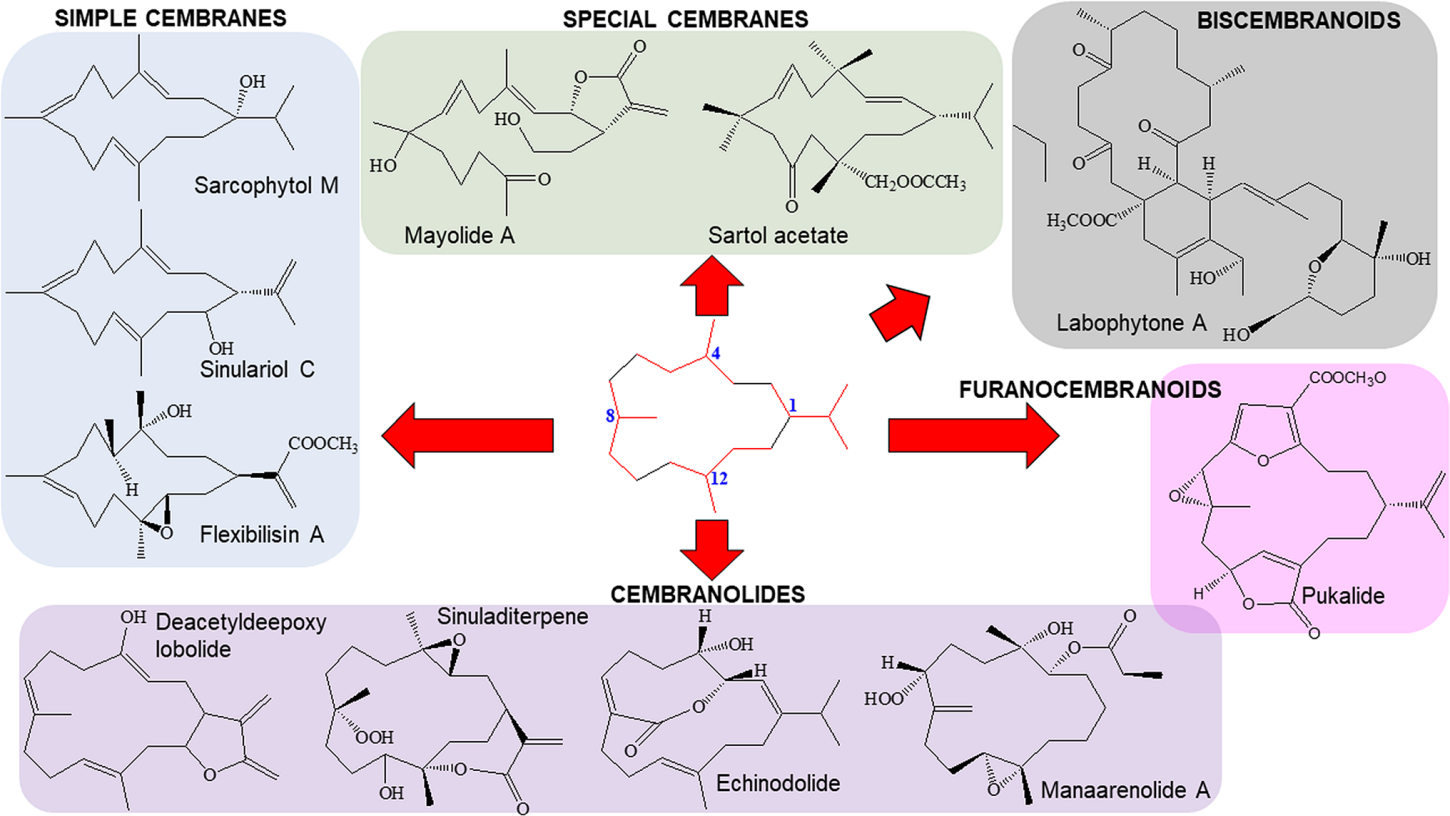
Chemical structures of chembranoid molecules. The isoprene unit of the basic carbon skeleton of cembranoids is bonded head-to-tail (red colors)

Cembranoids are derived from the cyclization of geranylgeranyl pyrophosphate [12], as shown from the double bonds of the cembrane skeleton having the E geometry observed in geranylgeraniol, diterpene alcohol. Theyare a class of isoprenoid and consist of a fourteen-membered carbocyclic ring with an isopropyl residue at position 1, and three methyl groups at positions 4, 8, and 12 [9, 13, 14]. Cembrane diterpenoids have diverse structural variations with many functional groups (lactone, epoxide, furan, ester, aldehyde, hydroxyl, carboxyl moieties) and cyclizations that allow them to be grouped into several families [15, 16]. According to the review of Yang et al. [15], the cembrane-type diterpenoids may be classified as shown in Fig. 1, which are:

1. Simple cembranes include the isopropyl cembranes, isopropenyl cembranes, and isopropyl/isopropenyl acid cembranes subtypes.
2. Cembranolides possess a 14-membered carbocyclic nucleus generally fused to a 5-, 6-, 7-, or 8-membered lactone ring. Cembranolides include the subtypes 5-membered lactone, 6-membered lactone, 7-membered lactone, 8-membered lactone.
3. Furanocembranoids possess a 14-membered carbocyclic nucleus as well as a furan heterocycle. They also have a butenolide moiety involving C-10–C-12 and C-20.
4. Biscembranoids possess a 14-6-14 membered tricyclic backbone of tetraterpenoids.
5. Special cembranes include the subtypes secocembranes, 13-membered carbocyclic cembranoids, cembrane glycosides, cembrane africanane, and other cembranes.

This review highlights secondary metabolites isolated from the genera *Sarcophyton*, *Sinularia*, *Lobophytum* and their biological activities reported in the literature between 2016 to mid-2020. The literatures were collected from different online databases, including Pubmed and Google Scholar, presenting the research progress on secondary metabolites isolated from soft corals within the last five years. This review summarizes the potential application of biomolecules (360 compounds) isolated from these three genera, covering the chemistry as well as the biological activity of their secondary metabolites, with special reference to cembranoids.

## Cembranoids

### Cembranoids Reported from Genus Sarcophyton

A total of 169 cembranoid compounds were isolated from *Sarcophyton* collected from various geographical areas (Table 1). Out of those, 128 were new compounds and 41 were previously known compounds with newly discovered activities. Eleven of the new compounds were newly discovered and have not been thoroughly tested for their biological activities.

**Table 1:** The biological activities of cembranoid isolates from *genera Sarcophyton*

Entry	Compound name (number)	Novelty	Sources	Geographical area of collection	Biological activities	References
1	16-hydroxycembra-1,3,7,11-tetraene (**1**)	New	*Sarcophyton sp.*	Karah Island, Terengganu, West Malaysia	Anti-bacterial activity against *Staphylococcus aureus* with MBC = 75 μg/mL, and MIC = 25 μg/mL	[17]
2	(2*S*,7*S*,8*S*)-sarcophytoxide (**2**)	Known	*Sarcophyton trocheliophorum*	Selayar Island, South Sulawesi, Indonesia	Moderate anti-bacterial activity against *Bacillus subtilis*, *Staphylococcus aureus*, and *Vibrio cholerae* with MIC = 125, 100, and 125 mg/mL, respectively	[18]
3	Bissublivide A (**3**)	New	*Sarcophyton subviride*	Xisha Islands in the South China Sea	No anti-cancer activity against MG-63, A549 and HuH7 with IC_50_ > 30 μM, > 25 μM, and μM 50 μM, respectively	[19]
4	Bissublivide B (**4**)	New	*Sarcophyton subviride*	Xisha Islands in the South China Sea	No anti-cancer activity against MG-63, A549 and HuH7 with IC_50_ > 30 μM, > 25 μM, and μM 50 μM, respectively	[19]
5	Sarcophytol D (**5**)	New	*Sarcophyton trocheliophorum*	Yalong Bay, Hainan Province, China	No Inhibitory effect toward PTP1B	[20]
6	Sarcophytrol E (**6**)	New	*Sarcophyton trocheliophorum*	Yalong Bay, Hainan Province, China	No Inhibitory effect toward PTP1B	[20]
7	Sarcophytrol F (**7**)	New	*Sarcophyton trocheliophorum*	Yalong Bay, Hainan Province, China	No Inhibitory effect toward PTP1B	[20]
8	Trochelian (**8**)	New	*Sarcophyton trocheliophorum*	Red Sea coast, north of Jeddah, Saudi Arabia	Anti-bacterial activity against *Acinetobacter baumannii*, *Eschericia coli*, *Klebsiella pneumonia, Pseudomonas aeruginosa, Staphylococcus aureus, Staphylococcus epidermidis,* and *Streptococcus pneumoniae* with MIC = 4.2, 6.0, 5.8, 5.2, 4.0, 5.7, and 6.0 μM, respectively	[21]
9	Sarcotrocheldiol A (**9**)	New	*Sarcophyton trocheliophorum*	Red Sea coast, north of Jeddah, Saudi Arabia	Very weak anti-bacterial activity against *Acinetobacter baumannii*, *Eschericia coli*, *Klebsiella pneumonia,* and *Pseudomonas aeruginosa* (MIC data not provided)	[21]
10	Sarcotrocheldiol B (**10**)	New	*Sarcophyton trocheliophorum*	Red Sea coast, north of Jeddah, Saudi Arabia	Very weak anti-bacterial activity against *Klebsiella pneumonia, Staphylococcus aureus,* and *Staphylococcus epidermidis* (MIC data not provided)	[21]
11	Sarcophytonoxide A (**11**)	New	*Sarcophyton ehrenbergi*	North Reef (Beijiao) in the Xisha Islands of the South China Sea	No anti-cancer activity against A2780 with IC_50_ > 25 μM	[22]
12	Sarcophytonoxide B (**12**)	New	*Sarcophyton ehrenbergi*	North Reef (Beijiao) in the Xisha Islands of the South China Sea	No anti-cancer activity against A2780 with IC_50_ > 25 μM	[22]
13	Sarcophytonoxide C (**13**)	New	*Sarcophyton ehrenbergi*	North Reef (Beijiao) in the Xisha Islands of the South China Sea	No anti-cancer activity against A2780 with IC_50_ > 25 μM	[22]
14	Sarcophytonoxide D (**14**)	New	*Sarcophyton ehrenbergi*	North Reef (Beijiao) in the Xisha Islands of the South China Sea	No anti-cancer activity against A2780 with IC_50_ > 25 μM	[22]
15	Sarcophytonoxide E (**15**)	New	*Sarcophyton ehrenbergi*	North Reef (Beijiao) in the Xisha Islands of the South China Sea	No anti-cancer activity against A2780 with IC_50_ > 25 μM	[22]
16	9-hydroxy-10,11-dehydro-sarcotrocheliol (**16**)	New	*Sarcophyton trocheliophorum*	Egyptian Red Sea off the coast of Hurghada	No anti-bacterial activity against *Eschericia coli, Candida albicans, Mucor miehei, Chlorella vulgaris, Chlorella sorokiniana, Scenedesmus subspicatus, Rhizoctania solani,* and *Phytium ultimum* at 40 μg per disk. No cytotoxicity against brine shrimp at 10 μg/mL	[23]
17	Sarelengan A (**17**)	New	*Sarcophyton elegans*	Xisha Islands in the South China Sea	No anti-inflammatory activity by inhibtion on NO production in RAW 264.7	[24]
18	Sarelengan B (**18**)	New	*Sarcophyton elegans*	Xisha Islands in the South China Sea	Anti-inflammatory activity by inhibition on NO production in RAW 264.7 with IC_50_ = 18.2 μM	[24]
19	Sarelengan C (**19**)	New	*Sarcophyton elegans*	Xisha Islands in the South China Sea	Anti-inflammatory activity by inhibition on NO production in RAW 264.7 with IC_50_ = 32.5 μM	[24]
20	Sarelengan D (**20**)	New	*Sarcophyton elegans*	Xisha Islands in the South China Sea	No anti-inflammatory activity by inhibition on NO production in RAW 264.7	[24]
21	Sarelengan E (**21**)	New	*Sarcophyton elegans*	Xisha Islands in the South China Sea	No anti-inflammatory activity by inhibition on NO production in RAW 264.7	[24]
22	Sarelengan F (**22**)	New	*Sarcophyton elegans*	Xisha Islands in the South China Sea	No anti-inflammatory activity by inhibition on NO production in RAW 264.7	[24]
23	Sarelengan G (**23**)	New	*Sarcophyton elegans*	Xisha Islands in the South China Sea	No anti-inflammatory activity by inhibition on NO production in RAW 264.7	[24]
24	Sarcoehrenbergilid A (**24**)	New	*Sarcophyton ehrenbergi*	Egyptian Red Sea off the coast of Hurghada	Moderate anti-cancer activity against A549 with IC_50_ = 50.1 μM; low anti-cancer activity against HepG2 with IC_50_ = 98.6 μM. No anti-cancer activity against Caco2 with IC_50_ > 100 μM	[25]
25	Sarcoehrenbergilid B (**25**)	New	*Sarcophyton ehrenbergi*	Egyptian Red Sea off the coast of Hurghada	Low anti-cancer activity against A549 with IC_50_ = 76.4 μM; no anti-cancer activity against Caco2 and HepG2 with IC_50_ > 100 μM	[25]
26	Sarcoehrenbergilid C (**26**)	new	*Sarcophyton ehrenbergi*	Egyptian Red Sea off the coast of Hurghada	Moderate anti-cancer activity against A549 and HepG2 with IC_50_ = 50.8, 53.8 μM, respectively; no anti-cancer activity against Caco2 with IC_50_ > 100 μM	[25]
27	Sarcophinone (**27**)	Known	*Sarcophyton glaucom*	Egyptian Red Sea off the coast of Hurghada	Moderate anti-cancer activity against HepG2 with EC_50_ = 11.32 μg/mL (35.78 nM)	[26]
28	8*-epi*-sarcophinone (**28**)	Known	*Sarcophyton glaucom*	Egyptian Red Sea off the coast of Hurghada	Moderate anti-cancer activity against HepG2 with EC_50_ = 11.32 μg/mL (35.78 nM)	[26]
29	( +)-7α,8β-dihydroxydeepoxysarcophine (**29**)	Known	*Sarcophyton glaucom*	Egyptian Red Sea off the coast of Hurghada	Moderate anti-cancer activity against HepG2 with EC_50_ = 17.84 μg/mL	[26]
30	Sinumaximol G (**30**)	Known	*Sarcophyton glaucom*	Egyptian Red Sea off the coast of Hurghada	Potent anti-cancer activity against HepG2 with EC_50_ = 9.97 μg/mL; moderate anti-proliferation activity against MCF-7 with IC_50_ = 24.97 ± 0.3 μg/mL	[26, 32]
31	Sarcophine (**31**)	Known	*Sarcophyton glaucom*	Egyptian Red Sea off the coast of Hurghada	Moderate anti-cancer activity against HepG2 with EC_50_ = 10.32 μg/mL; anti-inflammatory activity by inhibition on LPS-induced expression of iNOS protein at 50,100 μM and expression of COX2 at 25,50,100 μM in RAW 264.7; moderate anti-proliferation activity against MCF-7 with IC_50_ = 22.39 ± 0.2 μg/mL	[26, 32, 33]
32	( +)-(1*E*,3*E*,11*E*)-7,8-epoxycembra-1,3,11,15-tetraene (**32**)	New	*Sarcophyton stellatum*	Inner reef of Mahambo, Tamatave, Madagascar	Compound not tested	[27]
33	Sarcophytrol M (**33**)	New	*Sarophyton trocheliophorum*	Yalong Bay, Hainan Province, China	Compound not tested	[28]
34	Sarcophytrol N (**34**)	New	*Sarophyton trocheliophorum*	Yalong Bay, Hainan Province, China	Compound not tested	[28]
35	Sarcophytrol O (**35**)	New	*Sarophyton trocheliophorum*	Yalong Bay, Hainan Province, China	Compound not tested	[28]
36	Sarcophytrol P (**36**)	New	*Sarophyton trocheliophorum*	Yalong Bay, Hainan Province, China	Compound not tested	[28]
37	Sarcophytrol Q (**37**)	New	*Sarophyton trocheliophorum*	Yalong Bay, Hainan Province, China	Compound not tested	[28]
38	Sarcophytrol R (**38**)	New	*Sarophyton trocheliophorum*	Yalong Bay, Hainan Province, China	Compound not tested	[28]
39	Sarcophytrol S (**39**)	New	*Sarophyton trocheliophorum*	Yalong Bay, Hainan Province, China	Compound not tested	[28]
40	Sarcophytrol T (**40**)	New	*Sarophyton trocheliophorum*	Yalong Bay, Hainan Province, China	Compound not tested	[28]
41	Sarcophytrol U (**41**)	New	*Sarophyton trocheliophorum*	Yalong Bay, Hainan Province, China	Compound not tested	[28]
42	2-hydroxy-crassocolide E (**42**)	New	*Sarcophyton sp.*	Western side of Mahengetang Island, Indonesia	Anti-cancer activity against MCF7 with GI_50_ = 18.13 ppm	[29]
43	Sarcophytoxide (**43**)	Known	*Sarcophyton sp.*	Western side of Mahengetang Island, Indonesia	Anti-cancer activity against MCF7 with GI_50_ = 12.22 ppm	[29]
44	Sarcassin E (**44**)	Known	*Sarcophyton sp.*	Western side of Mahengetang Island, Indonesia	Anti-cancer activity against MCF7 with GI_50_ = 24.2 ppm	[29]
45	3,7,11‐cembreriene‐2,15‐diol (**45**)	Known	*Sarcophyton sp.*	Western side of Mahengetang Island, Indonesia	Anti-cancer activity against MCF7 with GI_50_ = 22.27 ppm	[29]
46	11,12‐epoxy sarcophytol A (**46**)	Known	*Sarcophyton sp.*	Western side of Mahengetang Island, Indonesia	Anti-cancer activity against MCF7 with GI_50_ = 18.88 ppm	[29]
47	Sarcophytol A (**47**)	Known	*Sarcophyton sp.*	Western side of Mahengetang Island, Indonesia	Anti-cancer activity against MCF7 with GI_50_ = 20.041 ppm	[29]
48	Sarcophytrol G (**48**)	New	*Sarophyton trocheliophorum*	Yalong Bay, Hainan Province, China	No Inhibitory effect toward PTP1B	[30]
49	Sarcophytrol H (**49**)	New	*Sarophyton trocheliophorum*	Yalong Bay, Hainan Province, China	No Inhibitory effect toward PTP1B	[30]
50	Sarcophytrol I (**50**)	New	*Sarophyton trocheliophorum*	Yalong Bay, Hainan Province, China	No Inhibitory effect toward PTP1B	[30]
51	Sarcophytrol J (**51**)	New	*Sarophyton trocheliophorum*	Yalong Bay, Hainan Province, China	No Inhibitory effect toward PTP1B	[30]
52	Sarcophytrol K (**52**)	New	*Sarophyton trocheliophorum*	Yalong Bay, Hainan Province, China	No Inhibitory effect toward PTP1B	[30]
53	Sarcophytrol L (**53**)	New	*Sarophyton trocheliophorum*	Yalong Bay, Hainan Province, China	No Inhibitory effect toward PTP1B	[30]
54	( +)-(6*R*)-6-hydroxyisosarcophytoxide (**54**)	New	*Sarcophyton mililatensis*	Weizhou Island, Beihai, Guangxi Autonomous Region, China	No anti-cancer activity against HL-60 and A-549 with IC_50_ > 10 μmol/L; no inhibitory activity toward TNF-α induced NFκB with < 50% inhibition at 20 μg/mL	[31]
55	( +)-(6*R*)-6-acetoxyisosarcophytoxide (**55**)	New	*Sarcophyton mililatensis*	Weizhou Island, Beihai, Guangxi Autonomous Region, China	No anti-cancer activity against HL-60 and A-549 with IC_50_ > 10 μmol/L; no inhibitory activity toward TNF-α induced NFκB with < 50% inhibition at 20 μg/mL	[31]
56	( +)-17-hydroxyisosarcophytoxide (**56**)	New	*Sarcophyton mililatensis*	Weizhou Island, Beihai, Guangxi Autonomous Region, China	No anti-cancer activity against HL-60 and A-549 with IC_50_ > 10 μmol/L; no inhibitory activity toward TNF-α induced NFκB with < 50% inhibition at 20 μg/mL	[31]
57	Sarcomililatin A (**57**)	New	*Sarcophyton mililatensis*	Weizhou Island, Beihai, Guangxi Autonomous Region, China	No anti-cancer activity against HL-60 and A-549 with IC_50_ > 10 μmol/L; moderate inhibitory activity toward TNF-α induced NFκB with IC_50_ = 35.23 ± 12.42 μmol/L	[31]
58	Sarcomililatin B (**58**)	New	*Sarcophyton mililatensis*	Weizhou Island, Beihai, Guangxi Autonomous Region, China	No anti-cancer activity against HL-60 and A-549 with IC_50_ > 10 μmol/L; no inhibitory activity toward TNF-α induced NFκB with < 50% inhibition at 20 μg/mL	[31]
59	Sarcomiliatin C (**59**)	New	*Sarcophyton mililatensis*	Weizhou Island, Beihai, Guangxi Autonomous Region, China	No anti-cancer activity against HL-60 and A-549 with IC_50_ > 10 μmol/L; no inhibitory activity toward TNF-α induced NFκB with < 50% inhibition at 20 μg/mL	[31]
60	Sarcomililatin D (**60**)	New	*Sarcophyton mililatensis*	Weizhou Island, Beihai, Guangxi Autonomous Region, China	No anti-cancer activity against HL-60 and A-549 with IC_50_ > 10 μmol/L; no inhibitory activity toward TNF-α induced NFκB with < 50% inhibition at 20 μg/mL	[31]
61	Sarcomililatol (**61**)	New	*Sarcophyton mililatensis*	Weizhou Island, Beihai, Guangxi Autonomous Region, China	No anti-cancer activity against HL-60 and A-549 with IC_50_ > 10 μmol/L; no inhibitory activity toward TNF-α induced NFκB with < 50% inhibition at 20 μg/mL	[31]
62	( +)-isosarcophytoxide (**62**)	Known	*Sarcophyton mililatensis*	Weizhou Island, Beihai, Guangxi Autonomous Region, China	Strong anti-cancer activity against HL-60 and A549 with IC_50_ = 0.78 ± 0.21 and 1.26 ± 0.80 μmol/L, respectively; moderate inhibitory activity toward TNF-α induced NFκB with IC_50_ = 22.52 ± 4.44 μmol/L	[31]
63	Stellatumolide A (**63**)	New	*Sarcophyton stellatum*	The coast of Dongsha Atoll, Taiwan	No anti-cancer activity against HepG2, MDA-MBA231, and A549 with IC_50_ > 20 μg/mL	[33]
64	Stellatumolide B (**64**)	New	*Sarcophyton stellatum*	The coast of Dongsha Atoll, Taiwan	No anti-cancer activity against HepG2, MDA-MBA231, and A549 with IC_50_ > 20 μg/mL	[33]
65	Stellatumolide C (**65**)	New	*Sarcophyton stellatum*	The coast of Dongsha Atoll, Taiwan	No anti-cancer activity against HepG2, MDA-MBA231, and A549 with IC_50_ > 20 μg/mL	[33]
66	Stellatumonin A (**66**)	New	*Sarcophyton stellatum*	The coast of Dongsha Atoll, Taiwan	No anti-cancer activity against HepG2, MDA-MBA231, and A549 with IC_50_ > 20 μg/mL	[33]
67	Stellatumonin B (**67**)	New	*Sarcophyton stellatum*	The coast of Dongsha Atoll, Taiwan	No anti-cancer activity against HepG2, MDA-MBA231, and A549 with IC_50_ > 20 μg/mL	[33]
68	Stellatumonone (**68**)	New	*Sarcophyton stellatum*	The coast of Dongsha Atoll, Taiwan	No anti-cancer activity against HepG2, MDA-MBA231, and A549 with IC_50_ > 20 μg/mL	[33]
69	Cherbonolide A (**69**)	New	*Sarcophyton cherbonnieri*	Jihui Fish Port, Taiwan	Moderate anti-inflammatory activity by inhibition of fMLF/CB-induced superoxide anion generation and estalase release in human neutrophils with 32.1 ± 4.3 and 37.6 ± 5.0% inhibition at 30 μM, respectively	[34]
70	Cherbonolide B (**70**)	New	*Sarcophyton cherbonnieri*	Jihui Fish Port, Taiwan	Anti-inflammatory activity by inhibition of fMLF/CB-induced superoxide anion generation and estalase release in human neutrophils with 4.0 ± 6.7 and 23.5 ± 6.6% inhibition at 30 μM, respectively	[34]
71	Cherbonolide C (**71**)	New	*Sarcophyton cherbonnieri*	Jihui Fish Port, Taiwan	Moderate anti-inflammatory activity by inhibition of fMLF/CB-induced superoxide anion generation and estalase release in human neutrophils with 44.5 ± 4.6 and 35.6 ± 6.2% inhibition at 30 μM, respectively	[34]
72	Cherbonolide D (**72**)	New	*Sarcophyton cherbonnieri*	Jihui Fish Port, Taiwan	Anti-inflammatory activity by inhibition of fMLF/CB-induced superoxide anion generation and estalase release in human neutrophils with 6.4 ± 4.2 and 27.6 ± 6.4% inhibition at 30 μM, respectively	[34]
73	Cherbonolide E (**73**)	New	*Sarcophyton cherbonnieri*	Jihui Fish Port, Taiwan	Anti-inflammatory activity by inhibition of fMLF/CB-induced superoxide anion generation and estalase release in human neutrophils with 2.6 ± 6.2 and 30.5 ± 4.6% inhibition at 30 μM, respectively	[34]
74	Bischerbolide peroxide (**74**)	New	*Sarcophyton cherbonnieri*	Jihui Fish Port, Taiwan	Moderate anti-inflammatory activity by inhibition of fMLF/CB-induced superoxide anion generation and estalase release in human neutrophils with 64.6 ± 0.8 (IC_50_ = 26.2 ± 1.0 μM) and 42.6 ± 5.1% inhibition at 30 μM, respectively	[34]
75	Isosarcophine (**75**)	Known	*Sarcophyton cherbonnieri*	Jihui Fish Port, Taiwan	Anti-inflammatory activity by inhibition of fMLF/CB-induced superoxide anion generation and estalase release in human neutrophils with 3.5 ± 5.3 and 20.7 ± 4.1% inhibition at 30 μM, respectively	[34]
76	9-hydroxy-7,8-dehydro-sarcotrocheliol (**76**)	New	*Sarcophyton trocheliophorum*	Near Mahmieat of the Red Sea about ~ 1 km on the coast of Hurghada, East Egypt	No anti-bacterial activity against *Staphylococcus aureus, Bacillus subtilis, Streptomyces viridochromogenes, Escherichia coli, Mucor miehei, Candida albicans,* and *Chlorella vulgaris* at 40 μg/disc	[35]
77	8,9-expoy-sarcotrocheliol acetate (**77**)	New	*Sarcophyton trocheliophorum*	Near Mahmieat of the Red Sea about ~ 1 km on the coast of Hurghada, East Egypt	No anti-bacterial activity against *Staphylococcus aureus, Bacillus subtilis, Streptomyces viridochromogenes, Escherichia coli, Mucor miehei, Candida albicans,* and *Chlorella vulgaris* at 40 μg/disc	[35]
78	Sarcophytonolide S (**78**)	New	*Sarcophyton trocheliophorum*	Yalong Bay, Hainan Province, China	No inhibitory effect toward PTP1B	[36]
79	Sarcophytonolide T (**79**)	New	*Sarcophyton trocheliophorum*	Yalong Bay, Hainan Province, China	No inhibitory effect toward PTP1B	[36]
80	Sarcophytonolide U (**80**)	New	*Sarcophyton trocheliophorum*	Yalong Bay, Hainan Province, China	No inhibitory effect toward PTP1B	[36]
81	Sartrolide H (**81**)	New	*Sarcophyton trocheliophorum*	Yalong Bay, Hainan Province, China	Moderate inhibitory effect toward PTP1B with IC_50_ = 19.9 ± 3.13 μM	[36]
82	Sartrolide I (**82**)	New	*Sarcophyton trocheliophorum*	Yalong Bay, Hainan Province, China	No inhibitory effect toward PTP1B	[36]
83	Sartrolide J (**83**)	New	*Sarcophyton trocheliophorum*	Yalong Bay, Hainan Province, China	No inhibitory effect toward PTP1B	[36]
84	Sarcophytolide (**84**)	Known	*Sarcophyton trocheliophorum*	Yalong Bay, Hainan Province, China	Moderate inhibitory effect toward PTP1B with IC_50_ = 15.4 ± 1.11 μM. Moderate anti-bacterial activity against *Staphylococcus aureus* with MIC_50_ = 250 μM	[36]
85	Sarcophytonolide V (**85**)	New	*Sarcophyton sp.*	Sepanggar Bay, North Borneo	Antifungal activity agains *O. humicola* and *H. milfordensis* with MIC 6.25 μg/mL	[37]
86	Glaucumolide A (**86**)	Known	*Sarcophyton trocheliophorum*	Xisha Islands in the South China Sea	Significantly induce CD3^+^ T cells proliferation and increase CD4^+^/CD8^+^ T cells ratio at 3 μM	[38]
87	Bistrochelide A (**87**)	New	*Sarcophyton trocheliophorum*	Xisha Islands in the South China Sea	Decrease CD4^=^/CD8^+^ T cells ratio on mice splenocytes at 3 μM	[38]
88	Bistrochelide B (**88**)	New	*Sarcophyton trocheliophorum*	Xisha Islands in the South China Sea	Significantly induce CD3^+^ T cells proliferation on mice splenocytes at 3 μM	[38]
89	Bistrochelide C (**89**)	New	*Sarcophyton trocheliophorum*	Xisha Islands in the South China Sea	Significantly increase CD4^+^/CD8^+^ T cells ratio on mice splenocytes at 3 μM	[38]
90	Bistrochelide D (**90**)	New	*Sarcophyton trocheliophorum*	Xisha Islands in the South China Sea	No effect on CD3^+^ T cells proliferation and CD4^+^/CD8^+^ T cells ratio on mice splenocytes at 3 μM	[38]
91	Bistrochelide E (**91**)	New	*Sarcophyton trocheliophorum*	Xisha Islands in the South China Sea	No effect on CD3^+^ T cells proliferation and CD4^+^/CD8^+^ T cells ratio on mice splenocytes at 3 μM	[38]
92	7-acetyl-8-epi-sinumaximol G (**92**)	New	*Sarcophyton sp.*	Egyptian Red Sea off the coast of Hurghada	Moderate anti-proliferation activity against MCF-7 with IC_50_ = 23.84 ± 0.2 μg/mL	[32]
93	8-epi-sinumaximol G (**93**)	New	*Sarcophyton sp.*	Egyptian Red Sea off the coast of Hurghada	Moderate anti-proliferation activity against MCF-7 with IC_50_ = 26.22 ± 0.1 μg/mL	[32]
94	12-acetyl-7, 12-epi-sinumaximol G (**94**)	New	*Sarcophyton sp.*	Egyptian Red Sea off the coast of Hurghada	Moderate anti-proliferation activity against MCF-7 with IC_50_ = 26.81 ± 0.2 μg/mL	[32]
95	12-hydroxysarcoph-10-ene (**95**)	New	*Sarcophyton sp.*	Egyptian Red Sea off the coast of Hurghada	Moderate anti-proliferation activity against MCF-7 with IC_50_ = 25.28 ± 0.3 μg/mL	[32]
96	8-hydroxy-epi-sarcophinone (**96**)	New	*Sarcophyton sp.*	Egyptian Red Sea off the coast of Hurghada	Moderate anti-proliferation activity against MCF-7 with IC_50_ = 27.2 ± 0.5 μg/mL	[32]
97	Sarcoehrenolide A (**97**)	New	*Sarcophyton ehrenbergi*	South China Sea	Moderate anti-inflammatory activity by TNF-α inhibition on RAW 264.7 with IC_50_ = 28.5 μM; no anti-cancer activity against A549, HT-29, SNU-398, and Capan-1 with IC_50_ > 50 μM	[39]
98	Sarcoehrenolide B (**98**)	New	*Sarcophyton ehrenbergi*	South China Sea	Moderate anti-inflammatory activity by TNF-α inhibition on RAW 264.7 with IC_50_ = 8.5 μM; no anti-cancer activity against A549, HT-29, SNU-398, and Capan-1 with IC_50_ > 50 μM	[39]
99	Sarcoehrenolide C (**99**)	New	*Sarcophyton ehrenbergi*	South China Sea	Compound not tested	[39]
100	Sarcoehrenolide D (**100**)	New	*Sarcophyton ehrenbergi*	South China Sea	Moderate anti-inflammatory activity by TNF-α inhibition on RAW 264.7 with IC_50_ = 27.3 μM; no anti-cancer activity against A549, HT-29, SNU-398, and Capan-1 with IC_50_ > 50 μM	[39]
101	Sarcoehrenolide E (**101**)	New	*Sarcophyton ehrenbergi*	South China Sea	No anti-inflammatory activity by TNF-α inhibition on RAW 264.7 with IC_50_ > 50 μM; no anti-cancer activity against A549, HT-29, SNU-398, and Capan-1 with IC_50_ > 50 μM	[39]
102	Ehrenbergol D (**102**)	Known	*Sarcophyton ehrenbergi*	South China Sea	Moderate anti-inflammatory activity by TNF-α inhibition on RAW 264.7 with IC_50_ = 24.2 μM; no anti-cancer activity against A549, HT-29, SNU-398, and Capan-1 with IC_50_ > 50 μM	[39]
103	Sarcoehrenbergilid D (**103**)	Known	*Sarcophyton ehrenbergi*	Egyptian Red Sea off the coast of Hurghada	Potent anti-cancer activity against A549 with IC_25_ = 23.3 μM; no anti-cancer activity against HepG2 and Caco-2 with IC_25_ > 100 μM	[40]
104	Sarcoehrenbergilid E (**104**)	Known	*Sarcophyton ehrenbergi*	Egyptian Red Sea off the coast of Hurghada	Potent anti-cancer activity against A549 with IC_25_ = 27.3 μM; weaker anti-cancer activity against HepG2 with IC_25_ = 22.6 μM; no anti-cancer activity against Caco-2 with IC_25_ > 100 μM	[40]
105	Sarcoehrenbergilid F (**105**)	Known	*Sarcophyton ehrenbergi*	Egyptian Red Sea off the coast of Hurghada	Potent anti-cancer activity against A549 with IC_25_ = 25.4 μM; weaker anti-cancer activity against HepG2 with IC_25_ = 31.8 μM; no anti-cancer activity against Caco-2 with IC_25_ > 100 μM	[40]
106	Sarcoglaucin A (**106**)	New	*Sarcophyton glaucum*	Xisha Islands (YaGong Island) of South China Sea	No anti-cancer activity against K562, HL-60, A549, BEL-7402, HCT-116, Hela and L-02; no anti-bacterial activity against Gram-negative and Gram-positive bacteria; no anti-fouling activity against barnacle *Balanus Amphitrite*	[41]
107	Sarcoglaucin B (**107**)	New	*Sarcophyton glaucum*	Xisha Islands (YaGong Island) of South China Sea	No anti-cancer activity against K562, HL-60, A549, BEL-7402, HCT-116, Hela and L-02; no anti-bacterial activity against Gram-negative and Gram-positive bacteria; anti-larval settlement activity at 25 ppm with adhesive rate of 6.52%. No anti-fouling activity against barnacle *Balanus Amphitrite*	[41]
108	Sarcoglaucin C (**108**)	New	*Sarcophyton glaucum*	Xisha Islands (YaGong Island) of South China Sea	No anti-cancer activity against K562, HL-60, A549, BEL-7402, HCT-116, Hela and L-02; no anti-bacterial activity against Gram-negative and Gram-positive bacteria; no anti-fouling activity against barnacle *Balanus Amphitrite*	[41]
109	Sarcoglaucin D (**109**)	New	*Sarcophyton glaucum*	Xisha Islands (YaGong Island) of South China Sea	No anti-cancer activity against K562, HL-60, A549, BEL-7402, HCT-116, Hela and L-02; no anti-bacterial activity against Gram-negative and Gram-positive bacteria; no anti-fouling activity against barnacle *Balanus Amphitrite*	[41]
110	Sarcoglaucin E (**110**)	New	*Sarcophyton glaucum*	Xisha Islands (YaGong Island) of South China Sea	No anti-cancer activity against K562, HL-60, A549, BEL-7402, HCT-116, Hela and L-02; no anti-bacterial activity against Gram-negative and Gram-positive bacteria; anti-larval settlement activity at 25 ppm with adhesive rate of 4.60%; no anti-fouling activity against barnacle *Balanus Amphitrite*	[41]
111	Sarcoglaucin F (**111**)	New	*Sarcophyton glaucum*	Xisha Islands (YaGong Island) of South China Sea	No anti-cancer activity against K562, HL-60, A549, BEL-7402, HCT-116, Hela and L-02; no anti-bacterial activity against Gram-negative and Gram-positive bacteria; no anti-fouling activity against barnacle *Balanus Amphitrite*	[41]
112	Sarcoglaucin G (**112**)	New	*Sarcophyton glaucum*	Xisha Islands (YaGong Island) of South China Sea	No anti-cancer activity against K562, HL-60, A549, BEL-7402, HCT-116, Hela and L-02; no anti-bacterial activity against Gram-negative and Gram-positive bacteria; no anti-fouling activity against barnacle *Balanus Amphitrite*	[41]
113	Sarcoglaucin H (**113**)	New	*Sarcophyton glaucum*	Xisha Islands (YaGong Island) of South China Sea	No anti-cancer activity against K562, HL-60, A549, BEL-7402, HCT-116, Hela and L-02; no anti-bacterial activity against Gram-negative and Gram-positive bacteria; no anti-fouling activity against barnacle *Balanus Amphitrite*	[41]
114	Sarcoglaucin I (**114**)	New	*Sarcophyton glaucum*	Xisha Islands (YaGong Island) of South China Sea	No anti-cancer activity against K562, HL-60, A549, BEL-7402, HCT-116, Hela and L-02; no anti-bacterial activity against Gram-negative and Gram-positive bacteria; no anti-fouling activity against barnacle *Balanus Amphitrite*	[41]
115	Trochelioid (**115**)	Known	*Sarcophyton glaucum*	Xisha Islands (YaGong Island) of South China Sea	No anti-cancer activity against K562, HL-60, A549, BEL-7402, HCT-116, Hela and L-02; no anti-bacterial activity against Gram-negative and Gram-positive bacteria; strong anti-fouling activity against *Balanus Amphitrite* with adhesive rate 8.19% at 25 ppm	[41]
116	7α-hydroxy-△^8(19)^- deepoxysarcophine (**116**)	Known	*Sarcophyton glaucum*	Xisha Islands (YaGong Island) of South China Sea	No anti-cancer activity against K562, HL-60, A549, BEL-7402, HCT-116, Hela and L-02; no anti-bacterial activity against Gram-negative and Gram-positive bacteria; strong anti-fouling activity against *Balanus Amphitrite* with adhesive rate 14.14% at 25 ppm	[41]
117	(−)-sartrochine (**117**)	Known	*Sarcophyton glaucum*	Xisha Islands (YaGong Island) of South China Sea	No anti-cancer activity against K562, HL-60, A549, BEL-7402, HCT-116, Hela and L-02; no anti-bacterial activity against Gram-negative and Gram-positive bacteria; strong anti-fouling activity against *Balanus Amphitrite* with adhesive rate 7.78% at 25 ppm	[41]
118	Sarcomililate A (**118**)	New	*Sarcophyton mililatensis*	Xigu Island, Hainan Province, China	Anti-proliferation activity against ConA-induced T cell proliferation and LPS-induced B cell proliferation with IC_50_ = 49.8 μM and 20.2 μM, respectively; no anti-cancer activity against A549, HT-29, Hep3B, and MDA-MB-436 at 50 μM	[42]
119	Sarcomililatol A (**119**)	New	*Sarcophyton mililatensis*	Xigu Island, Hainan Province, China	Anti-proliferation activity against ConA-induced T cell proliferation and LPS-induced B cell proliferation with IC_50_ = 38.9 μM and 22.1 μM, respectively; no anti-cancer activity against A549, HT-20, Hep3B, and MDA-MB-436 at 50 μM	[42]
120	Sarcomililatol B (**120**)	New	*Sarcophyton mililatensis*	Xigu Island, Hainan Province, China	No anti-proliferation activity against ConA-induced T cell proliferation, LPS-induced B cell proliferation, A549, HT-20, Hep3B, and MDA-MB-436 at 50 μM	[42]
121	Yalogene A (**121**)	Known	*Sarcophyton mililatensis*	Xigu Island, Hainan Province, China	Anti-proliferation activity against LPS-induced B cell proliferation with IC_50_ = 4.8 μM; no anti-cancer activity against ConA-induced T cell proliferation, A-549, HT-20, Hep3B, and MDA-MB-436 at 50 μM	[42]
122	Sarcophytol M (**122**)	Known	*Sarcophyton mililatensis*	Xigu Island, Hainan Province, China	Anti-proliferation activity against ConA-induced T cell proliferation and LPS-induced B cell proliferation with IC_50_ = 11.4 μM and 4.9 μM, respectively; no anti-cancer activity against A549, HT-29, Hep3B, and MDA-MB-436 at 50 μM	[42]
123	Sarcoehrenin A (**123**)	New	*Sarcophyton ehrenbergi*	Weizhou Island, Guangxi Province, China	No anti-inflammatory activitiy on TNF-α secretion inhibition by RAW 264.7 with IC_50_ > 50 μM	[43]
124	Sarcoehrenin B (**124**)	New	*Sarcophyton ehrenbergi*	Weizhou Island, Guangxi Province, China	No anti-inflammatory activitiy on TNF-α secretion inhibition by RAW 264.7 with IC_50_ > 50 μM	[43]
125	Sarcoehrenin C (**125**)	New	*Sarcophyton ehrenbergi*	Weizhou Island, Guangxi Province, China	No anti-inflammatory activitiy on TNF-α secretion inhibition by RAW 264.7 with IC_50_ > 50 μM	[43]
126	Sarcoehrenin D (**126**)	New	*Sarcophyton ehrenbergi*	Weizhou Island, Guangxi Province, China	No anti-inflammatory activitiy on TNF-α secretion inhibition by RAW 264.7 with IC_50_ > 50 μM	[43]
127	Sarcoehrenin E (**127**)	New	*Sarcophyton ehrenbergi*	Weizhou Island, Guangxi Province, China	No anti-inflammatory activitiy on TNF-α secretion inhibition by RAW 264.7 with IC_50_ > 50 μM	[43]
128	Sarcoehrenin F (**128**)	New	*Sarcophyton ehrenbergi*	Weizhou Island, Guangxi Province, China	No anti-inflammatory activitiy on TNF-α secretion inhibition by RAW 264.7 with IC_50_ > 50 μM	[43]
129	Sarcoehrenin G (**129**)	New	*Sarcophyton ehrenbergi*	Weizhou Island, Guangxi Province, China	Moderate anti-inflammatory activitiy on TNF-α secretion inhibition by RAW 264.7 with IC_50_ = 21.3 μM	[43]
130	Sarcoehrenin H (**130**)	New	*Sarcophyton ehrenbergi*	Weizhou Island, Guangxi Province, China	Moderate anti-inflammatory activitiy on TNF-α secretion inhibition by RAW 264.7 with IC_50_ = 30.8 μM	[43]
131	Sarcoehrenin I (**131**)	New	*Sarcophyton ehrenbergi*	Weizhou Island, Guangxi Province, China	No anti-inflammatory activitiy on TNF-α secretion inhibition by RAW 264.7 with IC_50_ > 50 μM	[43]
132	(2*S*,11*S*,12*S*)-isosarco phytoxide (**132**)	New	*Sarcophyton ehrenbergi*	Weizhou Island, Guangxi Province, China	No anti-inflammatory activitiy on TNF-α secretion inhibition by RAW 264.7 with IC_50_ > 50 μM	[43]
133	Sarcoehrenin J (**133**)	New	*Sarcophyton ehrenbergi*	Weizhou Island, Guangxi Province, China	Moderate anti-inflammatory activitiy on TNF-α secretion inhibition by RAW 264.7 with IC_50_ = 38.6 μM	[43]
134	(13*S*)-cembra-1,3,7,11-tetraen-13-ol (**134**)	Known	*Sarcophyton ehrenbergi*	Weizhou Island, Guangxi Province, China	Potent anti-inflammatory activitiy on TNF-α secretion inhibition by RAW 264.7 with IC_50_ = 9.1 μM	[43]
135	(+)-sarcophtol (**135**)	Known	*Sarcophyton ehrenbergi*	Weizhou Island, Guangxi Province, China	Moderate anti-inflammatory activitiy on TNF-α secretion inhibition by RAW 264.7 with IC_50_ = 15.4 μM	[43]
136	Cembrene-C (**136**)	Known	*Sarcophyton ehrenbergi*	Weizhou Island, Guangxi Province, China	Moderate anti-inflammatory activitiy on TNF-α secretion inhibition by RAW 264.7 with IC_50_ = 29.5 μM	[43]
137	(1*R*,4*R*,2*E*,7*E*,11*E*)-cembra-2,7,11-trien-4-ol (**137**)	Known	*Sarcophyton ehrenbergi*	Weizhou Island, Guangxi Province, China	Moderate anti-inflammatory activitiy on TNF-α secretion inhibition by RAW 264.7 with IC_50_ = 12.5 μM	[43]
138	(1*S*,4*R*,2*E*,7*E*,11*E*)-cembratrien-4-ol (**138**)	Known	*Sarcophyton ehrenbergi*	Weizhou Island, Guangxi Province, China	Potent anti-inflammatory activitiy on TNF-α secretion inhibition by RAW 264.7 with IC_50_ = 7.2 μM	[43]
139	(7*S*,8*R*)-dihydroxy-deepoxysarcophine (**139**)	Known	*Sarcophyton glaucum*	Dahab, Ras Sudr, and Sharm El-Sheikh, Red Sea Coast	Anti-cancer activity against HEK293 with LD_50_ = 123.5 ± 13.00 mM. Neurological activity by competitive inhibition of neuronal glycine receptor with K_I_ = 109 ± 9 μM; no effect on strychnine toxicity in mouse experiment model	[44]
140	Sardigitolide A (**140**)	New	*Sarcophyton digitatum*	Collected from the wild and cultured in National Museum of Marine Biology and Aquarium, Taiwan	Not cytotoxic towards MCF-7, MDA-MB-231, HepG2, and HeLa; no anti-inflammatory activity on LPS-stimulated murine macrophage J774A.1 cell	[45]
141	Sardigitolide B (**141**)	New	*Sarcophyton digitatum*	Collected from the wild and cultured in National Museum of Marine Biology and Aquarium, Taiwan	Cytotoxic towards MCF-7 and MDA-MB-231 with IC_50_ of 9.6 ± 3.0 and 14.8 ± 4.0 µg/mL, respectively; no anti-inflammatory activity on LPS-stimulated murine macrophage J774A.1 cell	[45]
142	Sardigitolide C (**142**)	New	*Sarcophyton digitatum*	Collected from the wild and cultured in National Museum of Marine Biology and Aquarium, Taiwan	Not cytotoxic towards MCF-7, MDA-MB-231, HepG2, and HeLa; no anti-inflammatory activity on LPS-stimulated murine macrophage J774A.1 cell	[45]
143	Sardigitolide D (**143**)	New	*Sarcophyton digitatum*	Collected from the wild and cultured in National Museum of Marine Biology and Aquarium, Taiwan	Not cytotoxic towards MCF-7, MDA-MB-231, HepG2, and HeLa; no anti-inflammatory activity on LPS-stimulated murine macrophage J774A.1 cell	[45]
144	Sarcophytolide L (**144**)	Known	*Sarcophyton digitatum*	Collected from the wild and cultured in National Museum of Marine Biology and Aquarium, Taiwan	Not cytotoxic towards MCF-7, MDA-MB-231, HepG2, and HeLa; no anti-inflammatory activity on LPS-stimulated murine macrophage J774A.1 cell	[45]
145	Glaucumolide A (**145**)	Known	*Sarcophyton digitatum*	Collected from the wild and cultured in National Museum of Marine Biology and Aquarium, Taiwan	Cytotoxic towards MCF-7, HepG2, and HeLa cells with IC_50_ values of 10.1 ± 3.3; 14.9 ± 3.5; and 17.1 ± 4.5 µg/mL, respectively; showed anti-inflammatory activity through inhibiting the production of IL-1β to 68 ± 1% in LPS-stimulated murine macrophages J774A.1 at a concentration of 10 µg/mL with IC_50_ values of 10.7 ± 2.7 µg/mL	[45]
146	Glaucumolide B (**146**)	Known	*Sarcophyton digitatum*	Collected from the wild and cultured in National Museum of Marine Biology and Aquarium, Taiwan	Cytotoxic towards MCF-7, MDA-MB-231, and HepG2 cells with IC_50_ value of 9.4 ± 3.0 17.8 ± 4.5 14.9 ± 4.2 µg/mL, respectively; no anti-inflammatory activity on LPS-stimulated murine macrophage J774A.1 cell	[45]
147	Isosarcophytonolide D (**147**)	Known	*Sarcophyton digitatum*	Collected from the wild and cultured in National Museum of Marine Biology and Aquarium, Taiwan	Cytotoxic towards MCF-7 with IC_50_ value of 10.9 ± 4.3 µg/mL; showed anti-inflammatory activity through inhibiting the production of IL-1β to 56 ± 1% in LPS-stimulated murine macrophages J774A.1 at a concentration of 10 µg/mL with IC_50_ value of 14.9 ± 5.1 µg/mL	[45]
148	Sarcotenusene A (**148**)	New	*Sarcophyton tenuispiculatum*	Collected from southern Taiwan and cultured at the Graduate Institute of Natural Products, Kaohsiung Medical University, Taiwan	Inactive in PPAR-ɣ transcription factor assay; showed cytotoxicity against MCF-7 cell line with IC_50_ value of 34.3 ± 3.7 µm; inactive on cytotoxic assay towards MDA-MB-231, HepG2 and HeLa cell line; inactive in inflammatory assay in LPS-stimulated J774A.1 macrophage cell	[46]
149	Sarcotenusene B (**149**)	New	*Sarcophyton tenuispiculatum*	Collected from southern Taiwan and cultured at the Graduate Institute of Natural Products, Kaohsiung Medical University, Taiwan	Inactive in PPAR-ɣ transcription factor assay; inactive on cytotoxic assay towards MCF-7, MDA-MB-231, HepG2 and HeLa cell line; inactive in inflammatory assay in LPS-stimulated J774A.1 macrophage cell	[46]
150	Sarcotenusene C (**150**)	New	*Sarcophyton tenuispiculatum*	Collected from southern Taiwan and cultured at the Graduate Institute of Natural Products, Kaohsiung Medical University, Taiwan	Inactive in PPAR-ɣ transcription factor assay; inactive on cytotoxic assay towards MCF-7, MDA-MB-231, HepG2 and HeLa cell line; inactive in inflammatory assay in LPS-stimulated J774A.1 macrophage cell	[46]
151	(2S, 7S, 8S)-sarcophytoxide (**151**)	Known	*Sarcophyton tenuispiculatum*	Collected from southern Taiwan and cultured at the Graduate Institute of Natural Products, Kaohsiung Medical University, Taiwan	Inactive in PPAR-ɣ transcription factor assay; showed cytotoxicity against the MCF-7 and HepG2 cell line with an IC_50_ value of 37.6 ± 4.2 and 35.2 ± 4.4 µm, respectively; inactive on cytotoxic assay towards MDA-MB-231 and HeLa cell line; inactive in inflammatory assay in LPS-stimulated J774A.1 macrophage cell	[46]
152	(2S, 7R, 8R)-sarcophytoxide (**152**)	Known	*Sarcophyton tenuispiculatum*	Collected from southern Taiwan and cultured at the Graduate Institute of Natural Products, Kaohsiung Medical University, Taiwan	Inactive in PPAR-ɣ transcription factor assay; showed cytotoxicity against the MCF-7 and HepG2 cell line with an IC_50_ value of 33.3 ± 3.5 and 28.6 ± 3.4 µm, respectively; inactive on cytotoxic assay towards MDA-MB-231 and HeLa cell line; inactive in inflammatory assay in LPS-stimulated J774A.1 macrophage cell	[46]
153	Sarcophytonin F (**153**)	Known	*Sarcophyton tenuispiculatum*	Collected from southern Taiwan and cultured at the Graduate Institute of Natural Products, Kaohsiung Medical University, Taiwan	Inactive in PPAR-ɣ transcription factor assay; showed cytotoxicity against the MCF-7 and MDA-MB-231 cell line with an IC_50_ value of 30.1 ± 3.1 and 38.6 ± 5.0 µm, respectively; inactive on cytotoxic assay towards HepG2 and HeLa cell line; inactive in inflammatory assay in LPS-stimulated J774A.1 macrophage cell	[46]
154	3,4-dihydro-4α-hydroxy-∆2-sarcophine (**154**)	Known	*Sarcophyton tenuispiculatum*	Collected from southern Taiwan and cultured at the Graduate Institute of Natural Products, Kaohsiung Medical University, Taiwan	Inactive in PPAR-ɣ transcription factor assay; showed cytotoxicity against the MCF-7 and HepG2 cell line with an IC_50_ value of 24.3 ± 3.0 and 34.5 ± 4.2 µm, respectively; inactive on cytotoxic assay towards MDA-MB-231 and HeLa cell line; inactive in inflammatory assay in LPS-stimulated J774A.1 macrophage cell	[46]
155	A hydroperoxide obtained by autoxidation of dihydrofuranocembranoid (**155**)	Known	*Sarcophyton tenuispiculatum*	Collected from southern Taiwan and cultured at the Graduate Institute of Natural Products, Kaohsiung Medical University, Taiwan	Inactive in PPAR-ɣ transcription factor assay; showed cytotoxicity against the MCF-7 and HepG2 cell line with an IC_50_ value of 27.2 ± 4.0 and 36.4 ± 5.3 µm, respectively; inactive on cytotoxic assay towards MDA-MB-231 and HeLa cell line; inactive in inflammatory assay in LPS-stimulated J774A.1 macrophage cell	[46]
156	( +)-7*α*,8*β*-dihydroxydeepoxysarcophine (**156**)	Known	*Sarcophyton tenuispiculatum*	Collected from southern Taiwan and cultured at the Graduate Institute of Natural Products, Kaohsiung Medical University, Taiwan	Inactive in PPAR-ɣ transcription factor assay; showed anti-inflammatory activity through potentially inhibited IL-1β production to 56 ± 1% in LPS-stimulated murine macrophage J774A.1 cell at a concentration of 30 µm; inactive on cytotoxic assay towards MCF-7, MDA-MB-231, HepG2 and HeLa cell line	[46]
157	Sarcoroseolide A (**157**)	New	*Sarcophyton roseum*	Dahab, Red Sea, Egypt	Showed no anti-inflammatory activity via iNOS inhibition and/or Nrf-2 induction and no cytotoxicity activity toward SK-MEL, KB, BT-549, and SK-OV-3 cell lines and two kidney (LLC-PK1 and VERO) non-cancerous cell lines	[47]
158	Sarcoroseolide B (**158**)	New	*Sarcophyton roseum*	Dahab, Red Sea, Egypt	Showed anti-inflammatory activity via iNOS inhibition with IC_50_ of 50 µM.Showed no cytotoxicity activity toward SK-MEL, KB, BT-549, and SK-OV-3 cell lines and two kidney (LLC-PK1 and VERO) non-cancerous cell lines	[47]
159	Sarcoroseolide C (**159**)	New	*Sarcophyton roseum*	Dahab, Red Sea, Egypt	Showed no anti-inflammatory activity via iNOS inhibition and/or Nrf-2 induction and no cytotoxicity activity toward SK-MEL, KB, BT-549, and SK-OV-3 cell lines and two kidney (LLC-PK1 and VERO) non-cancerous cell lines	[47]
160	Sarcoroseolide D (**160**)	New	*Sarcophyton roseum*	Dahab, Red Sea, Egypt	Showed no anti-inflammatory activity via iNOS inhibition and/or Nrf-2 induction and no cytotoxicity activity toward SK-MEL, KB, BT-549, and SK-OV-3 cell lines and two kidney (LLC-PK1 and VERO) non-cancerous cell lines	[47]
161	2-*epi*-sarcophine (**161**)	Known	*Sarcophyton roseum*	Dahab, Red Sea, Egypt	Showed anti-inflammatory activity via Nrf-2 induction at 100 μM (2.1-fold), 50 μM (1.4-fold), and 25 μM (0.9-fold).. Showed no cytotoxicity activity toward SK-MEL, KB, BT-549, and SK-OV-3 cell lines and two kidney (LLC-PK1 and VERO) non-cancerous cell lines	[47]
162	2R,7R,8R-dihydroxydeepoxysarcophine (**162**)	Known	*Sarcophyton roseum*	Dahab, Red Sea, Egypt	Showed anti-inflammatory activity via iNOS inhibition with IC_50_ of 39 µM and Nrf-2 induction at 100 μM (1.8-fold), 50 μM (1.5-fold), and 25 μM (1.5-fold). Showed no cytotoxicity activity toward SK-MEL, KB, BT-549, and SK-OV-3 cell lines and two kidney (LLC-PK1 and VERO) non-cancerous cell lines	[47]
163	Cherbonolide F (**163**)	New	*Sarcophyton cherbonnieri*	Jihui Fish Port, Taiwan	Low and moderate activities on anti-inflammatory assay with inhibition of superoxide anion generation (11.0% ± 8.7%) and elastase release (35.1% ± 10.6%) at 30 µM	[48]
164	Cherbonolide G (**164**)	New	*Sarcophyton cherbonnieri*	Jihui Fish Port, Taiwan	Moderate and high activities on anti-inflammatory assay with inhibition of superoxide anion generation (29.8% ± 9.8%) and elastase release (48.2% ± 12.5%) at 30 µM	[48]
165	Cherbonolide H (**165**)	New	*Sarcophyton cherbonnieri*	Jihui Fish Port, Taiwan	High and moderate activities on anti-inflammatory assay with inhibition of superoxide anion generation (44.5% ± 7.9%) and elastase release (35.6% ± 10.7%) at 30 µM	[48]
166	Cherbonolide I (**166**)	New	*Sarcophyton cherbonnieri*	Jihui Fish Port, Taiwan	Low and moderate activities on anti-inflammatory assay with inhibition of superoxide anion generation (6.4% ± 7.3%) and elastase release (27.6% ± 12.8%) at 30 µM	[48]
167	Cherbonolide J (**167**)	New	*Sarcophyton cherbonnieri*	Jihui Fish Port, Taiwan	Low and moderate activities on anti-inflammatory assay with inhibition of superoxide anion generation (6.2% ± 5.5%) and elastase release (29.7% ± 11.1%) at 30 µM	[48]
168	Cherbonolide K (**168**)	New	*Sarcophyton cherbonnieri*	Jihui Fish Port, Taiwan	Low activities on anti-inflammatory assay with inhibition of superoxide anion generation (12.9% ± 11.4%) and elastase release (16.7% ± 10.2%) at 30 µM	[48]
169	Cherbonolide L (**169**)	New	*Sarcophyton cherbonnieri*	Jihui Fish Port, Taiwan	Low and moderate activities on anti-inflammatory assay with inhibition of superoxide anion generation (17.1% ± 11.6%) and elastase release (27.6% ± 12.0%) at 30 µM	[48]

**Fig. 2:**
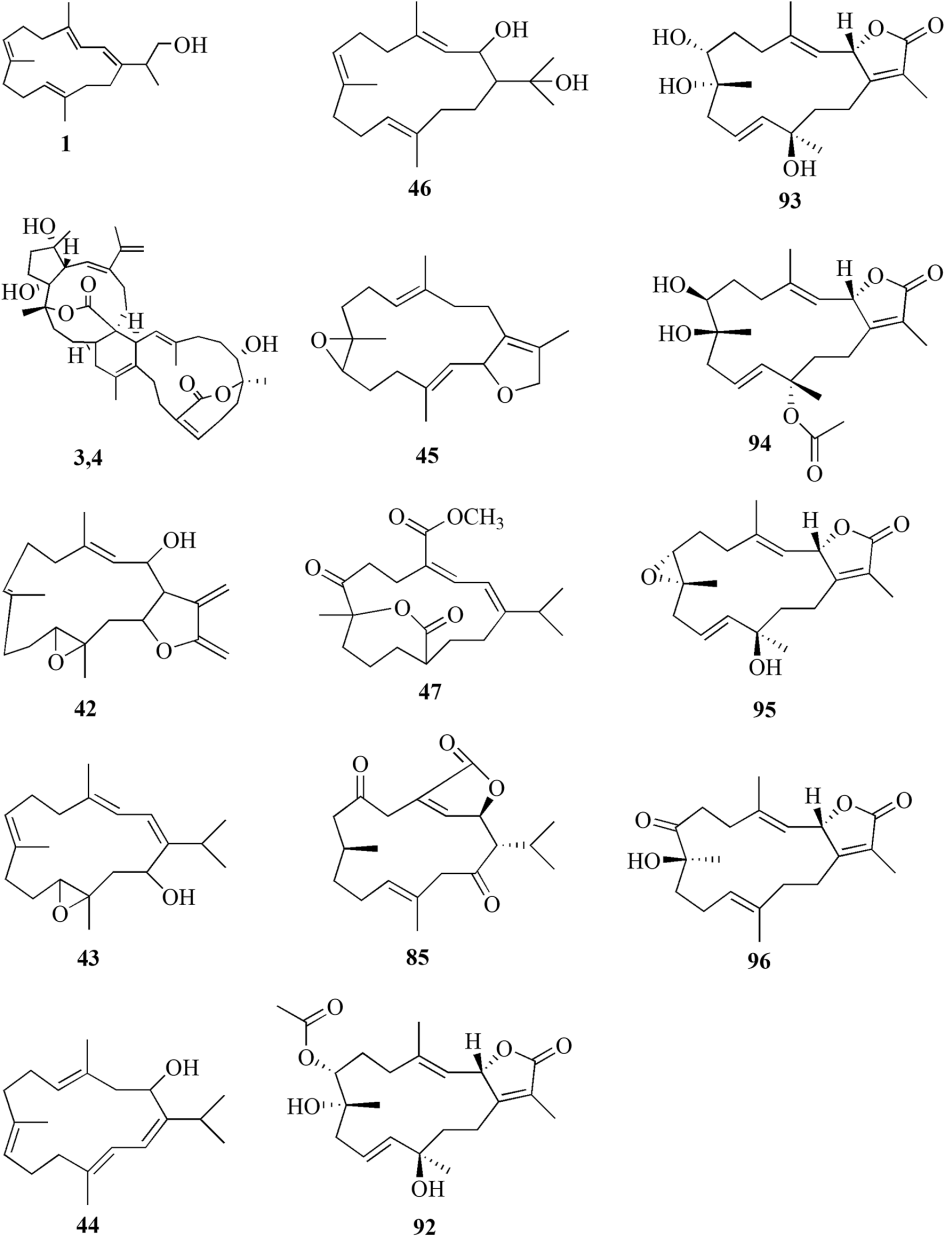
Cembranoids isolated from *Sarchophyton* sp. (**1, 42–47, 85, 92–96**) and *Sarcophyton subviride* (**3,4**)

**Fig. 3:**
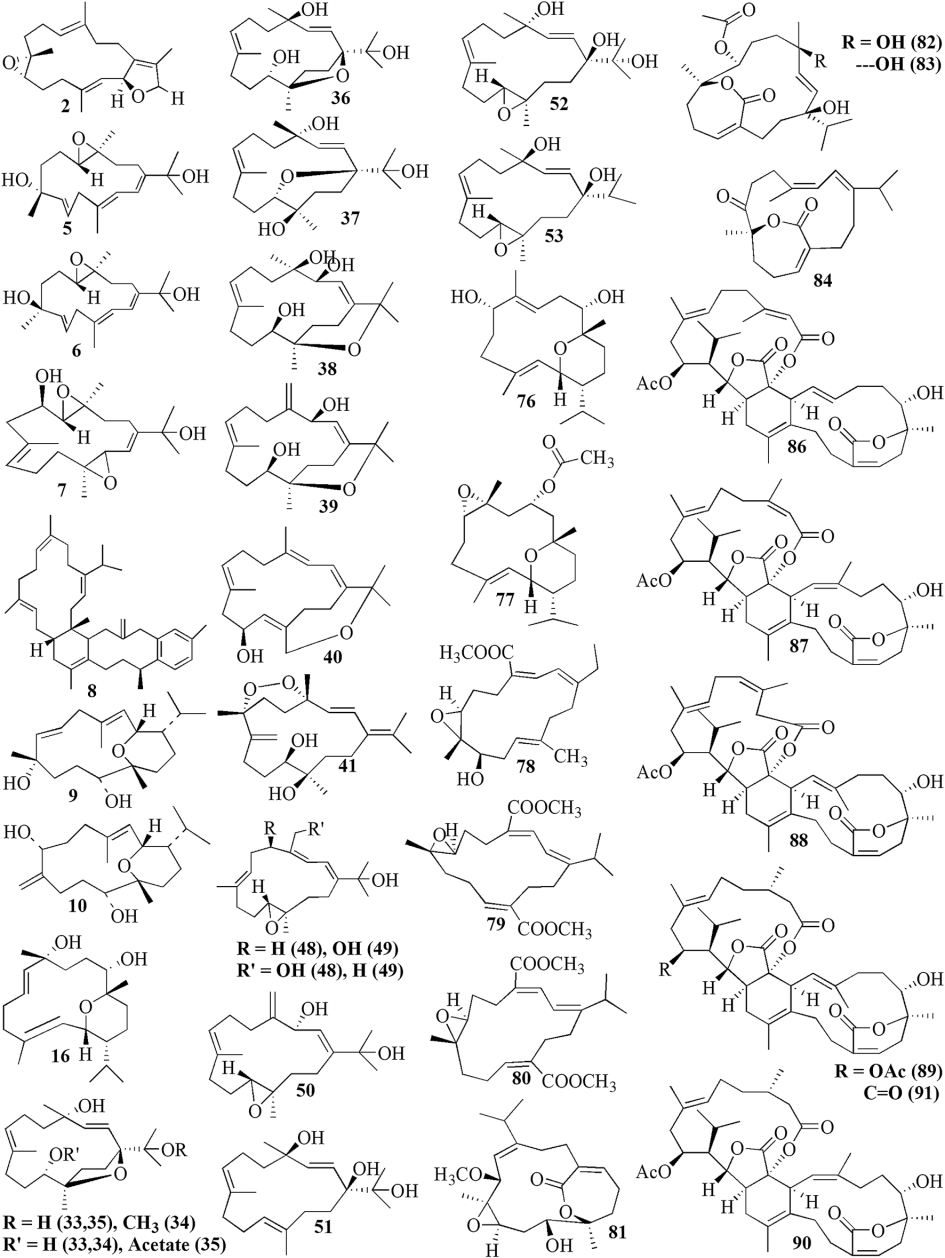
Cembranoids isolated from *Sarchophyton trocheliophorum*

Cembrane diterpenes have been isolated in a number of different locations. Fresh soft coral *Sarcophyton* sp. from Karah Island, Terengganu, West Malaysia yielded a new cembrane diterpene, 16-hydroxycembra-1,3,7,11-tetraene **1** (Fig. 2) [17]. The compound is a colorless oil, [α]D 25: − 9.3 (c 0.18, CHCl3) with the molecular formula of C_20_H_32_O (HR-MS m/z 289.2486 [M+H]+, calcd. 289.2526). A known compound cembranoid diterpene compound, sarcophytoxide **2,** was isolated as yellow crystalline needles (~ 0.5% yield) from the n-hexane fraction of *Sarcophyton trocheliophorum* collected in Selayar Island, South Sulawesi, Indonesia (Fig. 3). This compound has a molecular formula of C_20_H_30_O_2_ (m/z 325 [M+Na]^+^, ESI–MS positive ion) and been tested for its new anti-microbial activity (Table 1, entry 2) [18]. Two new biscembranoid-like compounds, bissubvilides A-B **3–4** were isolated from *Sacrophyton subviride* in Xisha Islands, South China Sea. These compounds have been tested for their anti-cancer activity but showed no activity (Table 1, entries 3,4) [19]. *S. trocheliophorum* from Yalong Bay, China, yielded three new highly oxidative cembranoids sarcophytols D-F **5–7**. Unfortunately, none of them showed activities on protein tyrosine phosphatase 1B (PTP1B) inhibitory effect (Table 1, entries 5–7) [20]. Another study isolated a new tetracyclic biscembrane hydrocarbon, trocheliane **8** (C_40_H_58_), along with two new cembranoid diterpenes, sarcotrocheldiols A-B **9–10** (C_20_H_34_O_3_), from the same species in Red Sea coast, Saudi Arabia [21]. These cembranoids were isolated as gummy materials with m/z of 538.4528 (M^+^, HREIMS) and 322.2500 (M^+^, HREIMS), respectively.

**Fig. 4:**
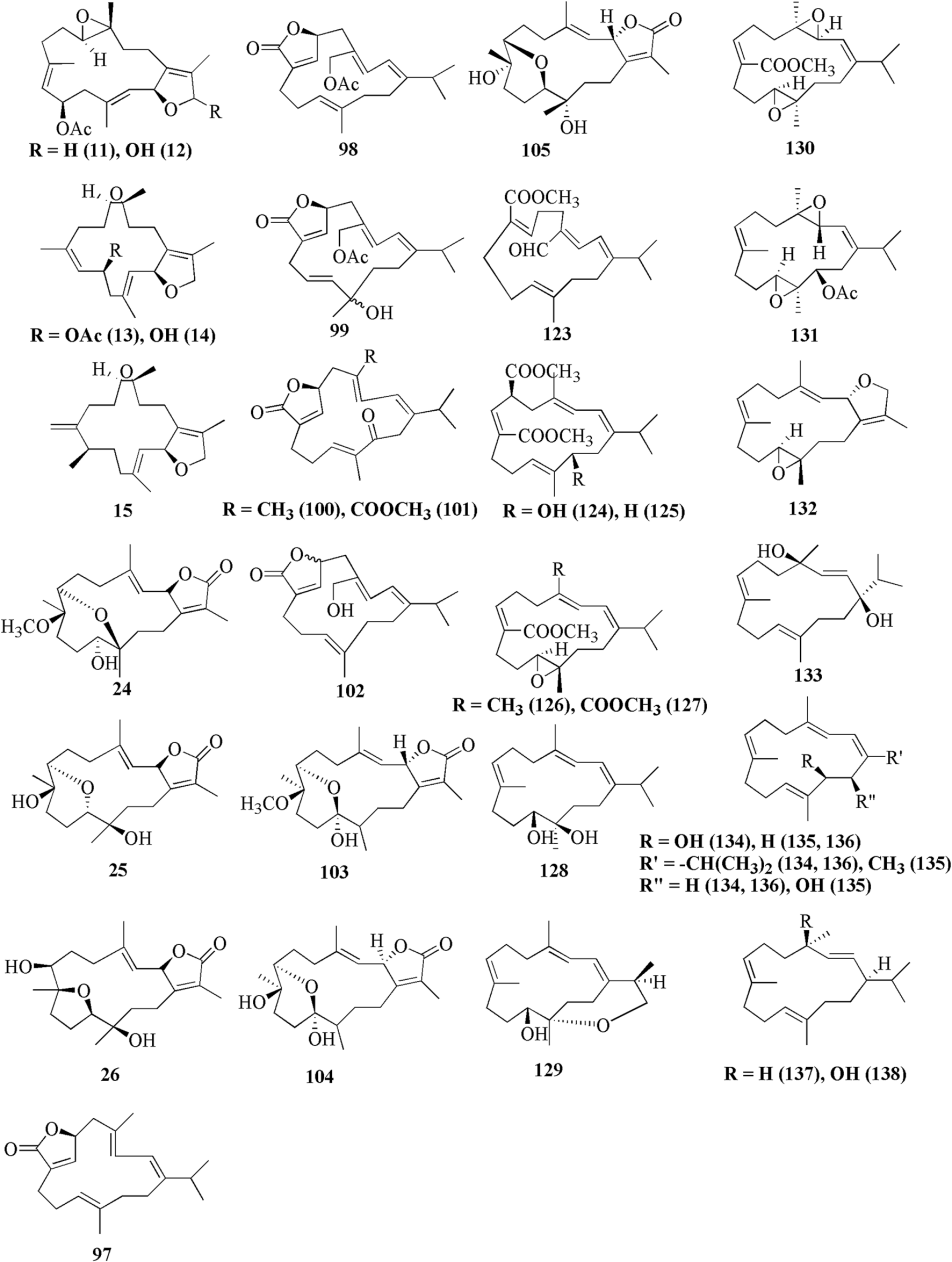
Cembranoids isolated from *Sarchophyton ehrenbergi*

Five new compounds, sarcophytonoxides A-E **11–15** were isolated from *Sarcophyton ehrenbergi* in North Reed (Beijiao) in the Xisha Islands, South China Sea (Fig. 4). HRESIMS analysis revealed sarcophytonoxides A, C and E are isomers with the molecular formula of C_22_H_32_O_4_. Meanwhile, sarcophytonoxides B and D have molecular formula of C_22_H_32_O_5_ and C_20_H_30_O_3_, respectively. However, these compounds have been tested for anti-cancer activity against human ovarian cancer cell line A2780, however, they showed no effect (Table 1, entries 11–15) [22]. A new pyrane-based cembranoid diterpene, 9-hydroxy-10,11-dehydro-sarcotrocheliol **16**, was isolated from *S. trocheliophorum*. However, this compound showed no anti-bacterial activity against multiple microorganisms (Table 1, entry 16) [23]. A study isolated two novel biscembranoids, sarelengans A-B **17**–**18**, along with five new cembranoids, sarelengans C-G **19–23** from *Sarcophyton elegans* in Xisha Islands, South China Sea with only **18** and **19** exhibited anti-inflammatory activity (Table 1, entries 17–23) [24]. *S. ehrenbergi* from the Egyptian Red Sea off the coast of Hurghada yielded three novel cembrene diterpenoids sarcoehrenbergilids A-C **24–26** [25]. Sarcoehrenbergilids A was found as a white crystal with a molecular formula of C_21_H_32_O_5_ (m/z at [M+Na]^+^ of 387.2142) while Sarcoehrenbergilids B and C were isomers observed as a white powder with a molecular formula of C_20_H_30_O_5_ (m/z at [M+Na]^+^ of 373.1986). Another species *Sarcophyton glaucom* from the same area was reported to yields five new diterpenes, sarcophinone **27**, 8-*epi*-sarcophinone **28**, (+)-7α,8β-dihydroxydeepoxysarcophine **29**, sinumaximol G **30**, and sarcophine **31** [26]. Several new cembranoids, (+)-(1*E*,3*E*,11*E*)-7,8-epoxycembra-1,3,11,15-tetraene **32** from *Sarcophyton stellatum* in Inner reef of Mahambo, Tamatave, Madagascar [27], and sarcophytrols M-U **33–41** from *S. trocheliophorum* in Yalong Bay, Hainan Province, China [28], was discovered but their activities have not been tested (Table 1, entries 32–41).

A study reported a new cembranoid, 2-hydroxy-crassocolide E **42**, and five known cembranoids, sarcophytoxide **43**, sarcassin E **44**, 3,7,11‐cembreriene‐2,15‐diol **45**, 11,12‐epoxy sarcophytol A **46**, and sarcophytol A **47** from *Sarcophyton* sp. in the western side of Mahengtang Island, Indonesia, with newly discovered anti-cancer activities against breast cancer MSF-7 (IC_50_ < 30 mg/L) (Table 1, entries 42–47) [29]. Six new cembranoids related to **33–41**, Sarcophytrols G-L **48–53** was also isolated from *S. trocheliophorum* from Yalong Bay, Hainan Province, China (Fig. 3). These compounds were tested for their inhibitory activity against PTP1B but showed no effect (Table 1, entries 48–53) [30]. Eight novel cembrane-type diterpenoids were also discovered from *Sarcophyton mililatensis* isolated from Guangxi Autonomous Region, China, namely (+)-(6R)-6-hydroxyisosarcophytoxide **54**, (+)-(6R)-6-acetoxyisosarcophytoxide **55**, (+)-17-hydroxyisosarcophytoxide **56**, sarcomililatins A-D **57–60**, and sarcomililatol **61**. Most of these compounds did not exhibit anti-cancer and anti-inflammatory activities, except for **57,** which showed a moderate anti-inflammatory activity (Table 1, entries 54–61). Along with these newly discovered compounds, a known compound (+)-isosarcophytoxide **62** was also isolated and reported to have strong anti-cancer and moderate anti-inflammatory activity (Table 1, entry 62) [31].

**Fig. 5:**
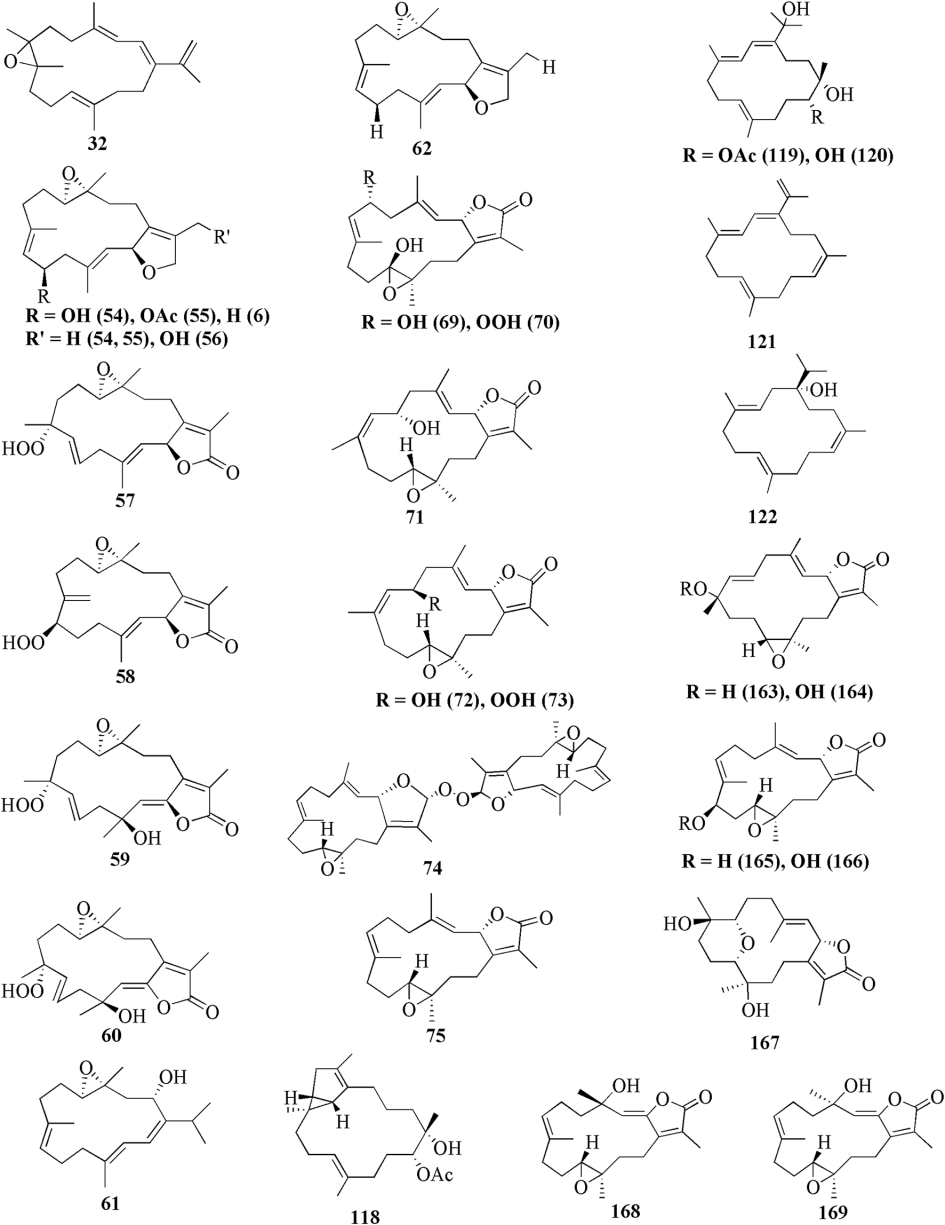
Cembranoids isolated from *Sarcophyton stellatum* (**32**), *Sarcophyton mililatensis* (**54–62, 118–122**), *Sarcophyton cherbonnieri* (**69–75, 163–169**)

*Sarcophyton stellatum* from the coast of Dongsha Atoll, Taiwan, was reported to yield seven new cembrane-based diterpenoids, stellatumolides A-C **63–65**, stellatumonins A-B **66–67**, and stellatumonone **68** (Fig. 5). Unfortunately, none of these compounds was found to have anti-cancer activity as tested (Table 1, entries 63–68) [33]. Within the same country, more precisely in Jihui Fish Port, a study reported five new cembranoids, cherbonolides A-E **69–73**, a biscembranoid peroxide, bischerbolide peroxide **74**, and a known cembranoid, isosarcophine **75**, from *Sarcophyton cherbonnieri* [34].

Several studies isolated new compounds as well as known compounds with newly discovered biological activities from *S. trocheliophorum* in three different locations. From near Mahmieat of the Red Sea, Hurghada, Eas Egypt, two new pyrane-based cembrane diterpenoids 9-hydroxy-7,8-dehydro-sarcotrocheliol **76**, and 8,9-expoy-sarcotrocheliol acetate **77**, were tested for their antibacterial activity but were proved as inactive (Table 1, entries 76,77) [35]. From Yalong Bay, Hainan Province, China, six new highly oxidative cembranoids were discovered, sarcophytonolides S-U **78–80**, and sartrolides **81–83**. These new compounds were tested for their anti-diabetic activity along with a known compound sarcophytolide **84**, but only **81** and **84** possessed the activity (Table 1, entries 78–84) [36]. A known biscembranoid, glaucumolide A **86**, together with five new biscembranoids, bistrochelides A-E **87–91,** were isolated from this species in Xisha Islands in the South China Sea. Following testing for their immunological activities, **86–89** were found to affect T-lymphocyte proliferation and differentiation, while **90–91** lacked this activity (Table 1, entries 86–91) [38].

A new cembranolide diterpene with anti-fungal activity, sarcophytonolide V **85**, was discovered from *Sarcophyton* sp. in Sepanggar Bay, North Borneo [37]. In the Egyptian Red Sea off the coast of Hurghada, a study on *Sarcophyton* sp. also isolated five new cembrane-type diterpenoids with moderate anti-cancer activity, namely 7-acetyl-8-epi-sinumaximol G **92**, 8-epi-sinumaximol-G **93**, 12-acetyl-7, 12-epi-sinumaximol G **94**, 12-hydroxysarcoph-10-ene **95**, and 8-hydroxy-epi-sarcophinone **96** (Table 1, entries 92–96) [32]. A study on *S. ehrenbergi* from South China Sea reported five new cembranoids, sarcoehrenolides A-E **97–101**, and a known cembranoid, ehrenbergol D **102**. Compound **99** has not been tested for its biological activities, while the others were tested for their anti-cancer properties but were found to be inactive (Table 1, entries 97–101). Most of these compounds have anti-inflammatory activity, except for **101** [39]. Another study on the same species from the Egyptian Red Sea off the coast of Hurghada isolated three known cembrene diterpenoids, sarcoehrenbergilids D-F **103–105**, which were reported to have anti-cancer activities (Table 1, entries, 103–105) [40].

**Fig. 6:**
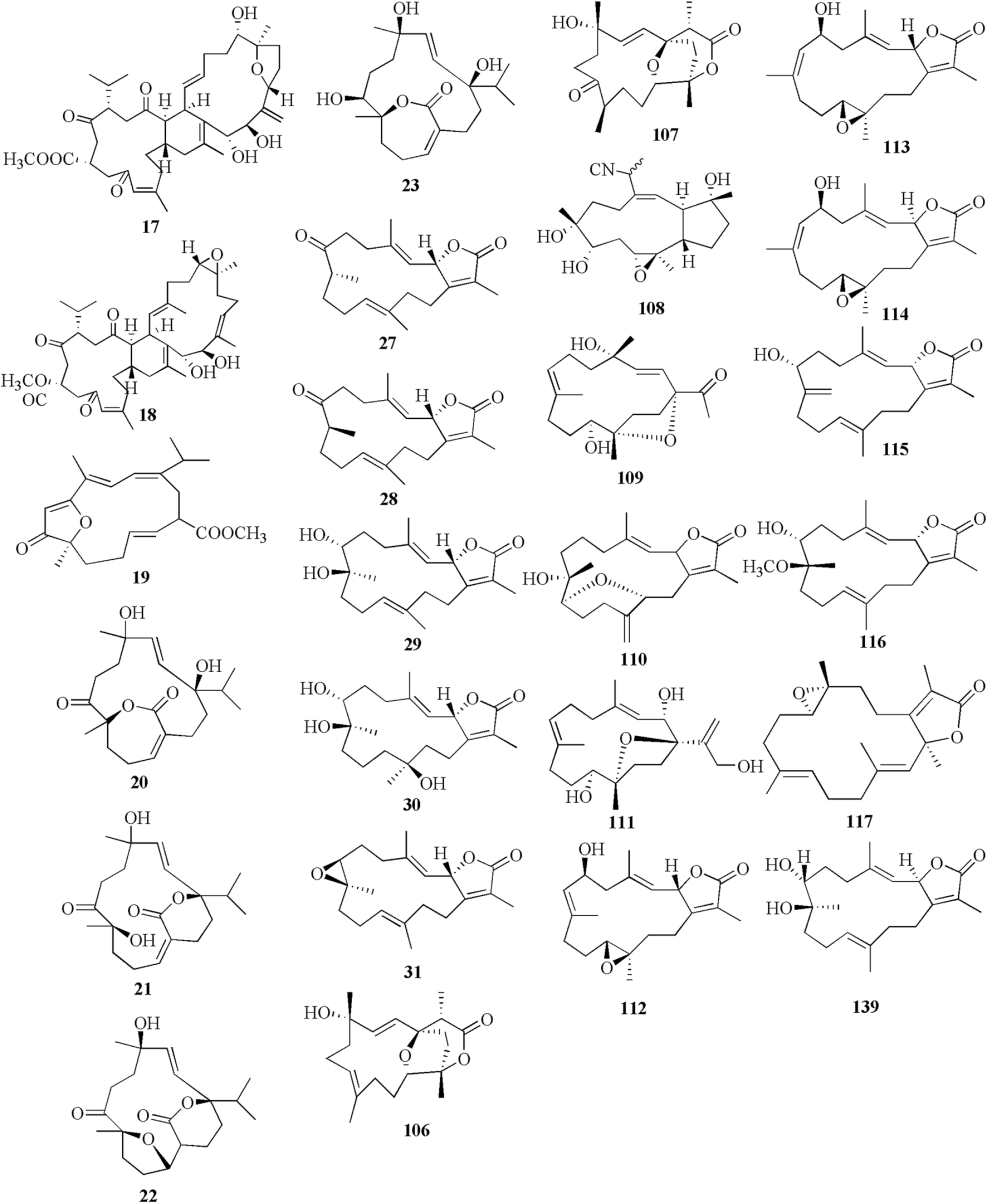
Cembranoids isolated from *Sarchophyton elegans* (***17–23,*** and *Sarchophyton glaucum* (**27–31, 106–117, 139**)

*Sarcophyton glaucum* from Xisha Islands of the South China Sea was reported to yield nine new cembrane diterpenes, sarcoglaucins A-I **106–114,** along with three known analogues, trochelioid **115**, 7α-hydroxy-△^8(19)^-deepoxysarcophine **116**, and (−)-sartrochine **117** (Fig. 6). None of them possessed anti-cancer and anti-bacterial activities (Table 1, entries 106–114) [41]. A new diterpenoid, sarcomililate A **118**, two new cembranoids, sarcomililatols A-B **119–120**, and two known related diterpenoids, yalogene A **121** and sarcophytol M **122**, were isolated from *Sarcophyton mililatensis* in Xigu Island, Hainan Province, China. Most of them were active as an anti-cancer agent, except for **120** (Table 1, entries 119–121) [42].

Another study on *S. ehrenbergi* from Weizhou Island, Guangxi Province, China, isolated eleven new cembrane diterpenes, sarcoehrenins A-I **123–131**, (2S,11S,12S)-isosarcophytoxide **132**, and sarcoehrenin J **133**. These compounds were tested for their anti-inflammatory potentials; however, only **129** and **130** were active. In addition, this study also discovered new anti-inflammatory activity on five known compounds within the same species, 13S)-cembra-1,3,7,11-tetraen-13-ol **134**, (+)-sarcophtol **135**, cembrene-C **136**, (1R,4R,2E,7E,11E)-cembra-2,7,11-trien-4-ol **137**, and (1S,4R,2E,7E,11E)-cembratrien-4-ol **138** (Table 1, entries 123–138) [43]. Lastly, a known trans-diol derivative of sarcophine, (7S, 8R)-dihydroxy-deepoxysarcophine **139** was isolated from *S. glaucum* in Dabah, Ras Sudr, and Sharm El-Sheikh, Red Sea Coast, and revealed that **139** exhibited anti-cancer and neurological activities (Table 1, entry 139) [44].

**Fig. 7:**
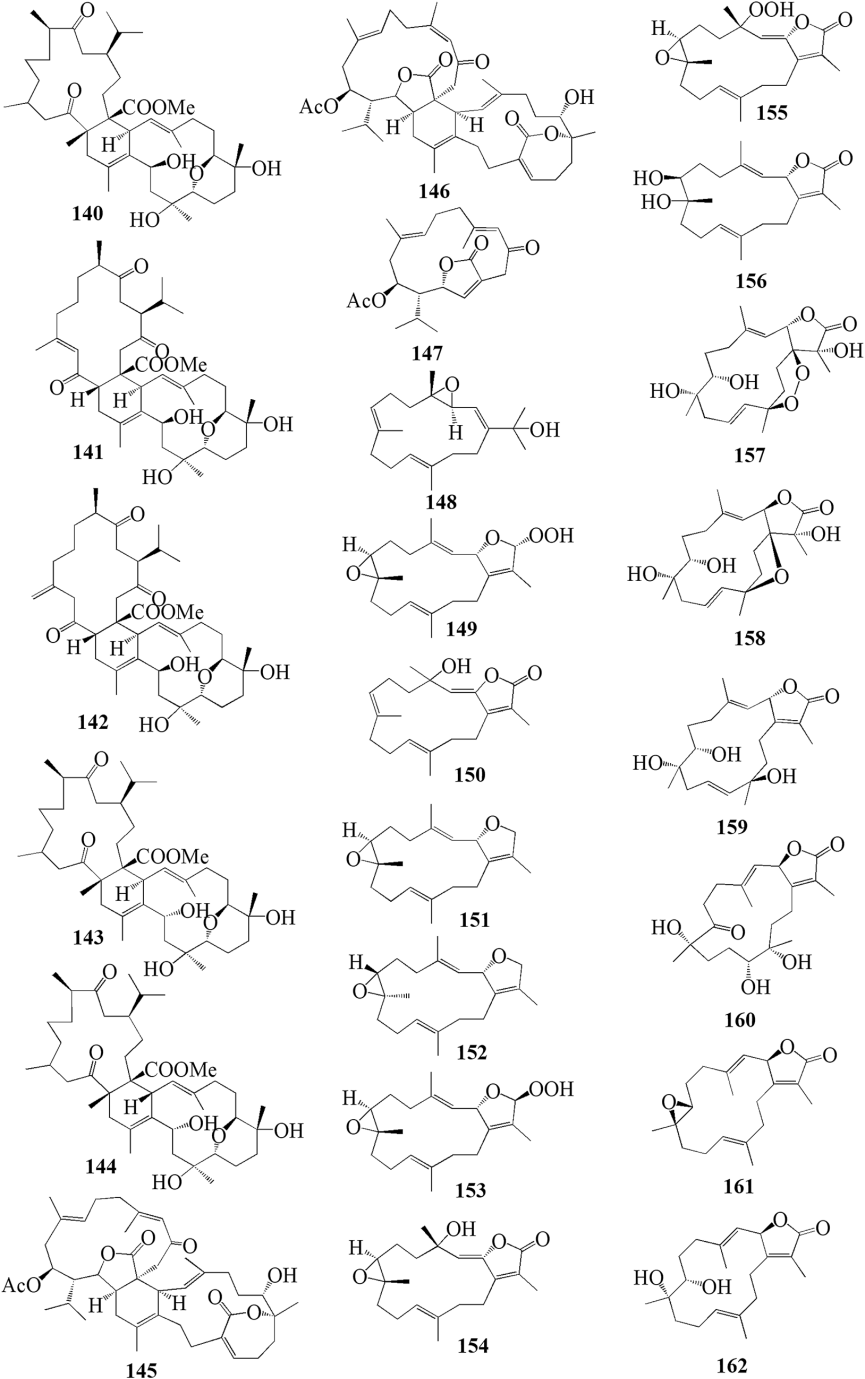
Cembranoids isolated from *Sarchophyton digitatum* (**140–147**), *Sarchophyton tenuispiculatum* (**148–156**) and *Sarchophyton roseum* (**157–162**)

Furthermore, S. *digitatum* which cultured in the National Museum of Marine Biology and Aquarium, Taiwan contained seven biscembranoids and one cembranoid. Four out of seven biscembranoids were unreported compounds namely sardigitolides A-D **140–143** (Fig. 7). The other three biscembranoids were reported before and namely sarcophytolide L **144** and glaucumolides A-B **145–146**. The only known cembranoid collected from this species namely isosarcophytonolide D **147**. The reported cembrane-type diterpenoid from S. *digitatum* was reported to display various anti-cancer and anti-inflammatory activities [45]. Another study reported nine cembranoids from *Sarcophyton tenuispiculatum* which culture at Kaohsiung Medical University, Taiwan. The three novel cembranoids sarcotenusenes A-C **148–150** were mostly inactive in PPAR-ɣ transcription factor assay; cytotoxic assay towards MCF-7, MDA-MB-231, HepG2 and HeLa cell line; and inflammatory assay. Moreover, (2S, 7S, 8S)-sarcophytoxide **151**, (2S, 7R, 8R)-sarcophytoxide **152**, sarcophytonin F **153**, 3,4-dihydro-4α-hydroxy-∆^2^-sarcophine **154**, A hydroperoxide obtained by autoxidation of dihydrofuranocembranoid **155**, and ( +)-7*α*,8*β*-dihydroxydeepoxysarcophine **156** were also displayed various results on the abovementioned assay [46]. Additionaly, six cembranoids were isolated from S. *roseum* collected in Dahab, Red Sea, Egypt. The new cembranoid sarcoroseolides A-D **157–160** and the known cembranoid 2-*epi*-sarcophine **161** and 2R,7R,8R-dihydroxydeepoxysarcophine **162** were being assessed for its anti-inflammatory and anti-cancer activities [47]. Lastly, *Sarcophyton cherbonnieri* collected from Jihui Fish Port, Taiwan, contained seven novel cembranoid that possessed various anti-inflammatory activities through inhibition of superoxide anion generation and elastase release, namely cherbonolides F-L **163–169** [48].

### Cembranoids from Genus Sinularia

**Fig. 8:**
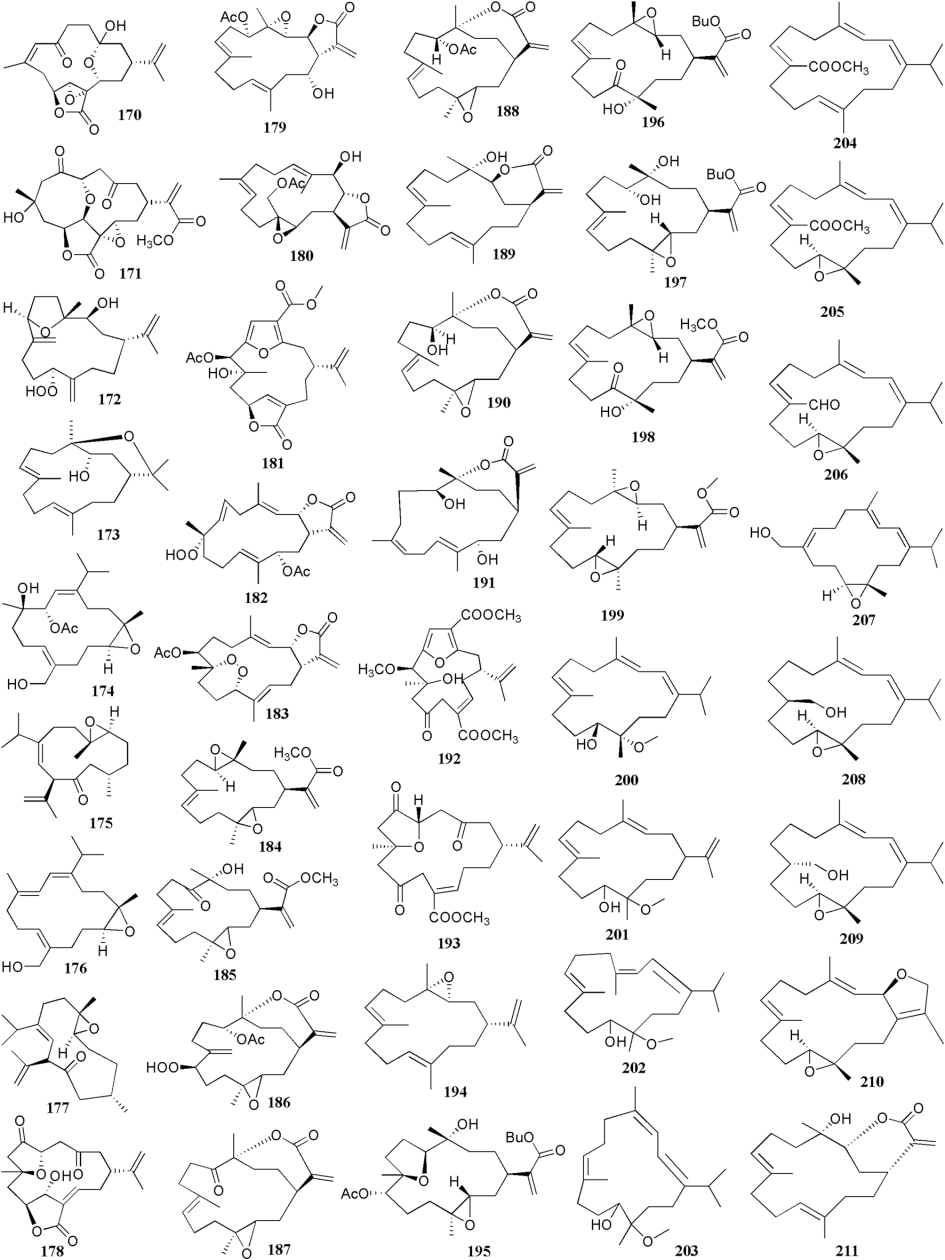
Cembranoids reported from *Sinularia* erecta (**170–172**), *Sinularia gravis* (**173**), *Sinularia nanolobata* (**174–177**), *Sinularia compacta* (**178–180**), *Sinularia sandensis* (**181**), *Sinularia* sp. (**182–183, 192–194, 400–203**), *Sinularia flexibilis* (**184–191, 195–199**) and *Sinularia scabra* (**204–211**)

The present study reported 42 cembranoid compounds isolated from *Sinularia* sp. collected from various geographical areas (Fig. 8). Twenty-nine of those were new compounds and the other 13 were previously known compounds with newly discovered activities. One of the new compounds was newly discovered and had not been thoroughly tested for their biological activities.

Soft coral *Sinularia erecta* from the South China Sea yielded three new norcembranoids, sinulerectols A-C **170–172** [49], whereas a new-non tested cembranoid diterpene named isodecaryiol **173** was collected from Madagascar *Sinularia gravis* [50]. Three new non-active cembranoids from Taiwan were isolated from S. *nanolobata* namely nanolobols A-C **174–176** along with one known biologically active cembranoid sinulariol C **177** [51]. *Sinularia compacta* from the South China Sea contained three new cembranoid diterpenes namely 5-*epi*-sinuleptolide **178**, michaolide F **179**, and 20-acetylsinularolide B **180** [53]. S. *sandensis* was reported to produce a known compound 7-acetylsinumaximol B **181** [55]. Kamada et al. isolated *Sinularia* sp. from Sabah, Malaysia and discovered a new cembranoid named sinularolide F **182** and a known cembranoid named denticulatolide **183** [56]. Taiwanese S. *flexibilis* produced seven compounds, three of which were new compounds with no biological activities named flexibilisins D-E **184–185** and flexibilisolide H **186** (Table 2, entries 17–19). The other four compounds were known compounds with various biological activities, namely 11-dehydrosinulariolide **187**¸ 11-*epi*-sinulariolide acetate **188**, (S)-14-deoxycrassin **189**, and sinulariolide **190** [57].

**Table 2:** The biological activities of cembranoid isolates from *genera Sinularia*

Entry	Compound name (number)	Novelty	Sources	Geographical area of collection	Biological activities	References
1	Sinulerectol A (**170**)	New	*Sinularia erecta*	Off the coast of Dongsha Atoll, north of the South China Sea	Anti-inflammatory activities through inhibition of superoxide generation and elastase release in fMLP/CB-induced human neutrophils with IC_50_ value of 2.3 ± 0.4 μM	[49]
2	Sinulerectol B (**171**)	New	*Sinularia erecta*	Off the coast of Dongsha Atoll, north of the South China Sea	Anti-inflammatory activities through inhibition of superoxide generation and elastase release in fMLP/CB-induced human neutrophils with IC_50_ value of 8.5 ± 0.3 μM	[49]
3	Sinulerectol C (**172**)	New	*Sinularia erecta*	Off the coast of Dongsha Atoll, north of the South China Sea	Anti-proliferation activity against K-562 cell line with IC_50_ value of 9.2 ± 3.3 μM	[49]
4	Isodecaryiol (**173**)	New	*Sinularia gravis*	Inner reef of Mahambo, Tamatave province at the east coast of Madagascar	Compound not tested	[50]
5	Nanolobol A (**174**)	New	*Sinularia nanolobata*	Off the coast of Jihui Fishing Port, Taitung county, Taiwan	Not cytotoxic against P388, K-562, HT-29	[51]
6	Nanolobol B (**175**)	New	*Sinularia nanolobata*	Off the coast of Jihui Fishing Port, Taitung county, Taiwan	Not cytotoxic against P388, K-562, HT-29	[51]
7	Nanolobol C (**176**)	New	*Sinularia nanolobata*	Off the coast of Jihui Fishing Port, Taitung county, Taiwan	Not cytotoxic against P388, K-562, HT-29	[51]
8	Sinulariol C (**177**)	Known	*Sinularia nanolobata*	Off the coast of Jihui Fishing Port, Taitung county, Taiwan	Anti-inflammatory activity through NO reduction on RAW 264.7 cells to 19.6% and 2.3% at concentration of 50 μM and 100 μM with high cell viability	[51]
9			*Sinularia scabra*	Off the coast of Xigu Island, Hainan Province, China	Strong inhibitory activity on the proliferation of Con A-induced T lymphocyte cells with IC_50_ value of 4.5 µM	[52]
10	5-*epi*-Sinuleptolide (**178**)	New	*Sinularia compacta*	Tongguling National Nature Reserve of Coral Reefs, South China Sea	Anti-proliferation activity against HCT-116 and A-549 with IC_50_ values of 10.1 and 14.7 μM, respectively	[53]
11			*Sinularia* sp.	Yongxing Island of Xisha Islands in the South China Sea	Anti-proliferation activity against HeLa and HCT-116 with IC_50_ values of 11.6 and 33.3 μM, respectively	[54]
12	Michaolide F (**179**)	New	*Sinularia compacta*	Tongguling National Nature Reserve of Coral Reefs, South China Sea	Exhibited lethality against brine shrimp *Artemia salina* with lethal ratio of 90.5% at concentration of 50 μg/mL	[53]
13	20-Acetylsinularolide B (**180**)	New	*Sinularia compacta*	Tongguling National Nature Reserve of Coral Reefs, South China Sea	Exhibited lethality against brine shrimp *Artemia salina* with lethal ratio of 90.0% at concentration of 50 μg/mL	[53]
14	7-Acetylsinumaximol B (7-AB) (**181**)	Known	*Sinularia sandensis*	Aquaculture	Exerted a concentration-dependent anti-proliferative effect on NCI-N87 cells and apoptosis induction. Anti-proliferation activity was associated with the release of cytochrome c from mitochondria, activation of pro-apoptotic proteins (such as caspase-3/-9, Bax and Bad), and inhibition of anti-apoptotic proteins (Bcl-2, Bcl-xL, and Mcl-1). 7-AB also triggered endoplasmic reticulum (ER) stress, leading to activation of the PERK/elF2α/ATF4/CHOP apoptotic pathway. 7-AB initiated autophagy in NCI-N87 cells and induced the expression of autophagy-related proteins, including Atg3, Atg5, Atg7, Atg12, LC3-I, and LC3-II	[55]
15	Sinularolide F (**182**)	New	*Sinularia* sp.	Mantanani Island, Sabah	Anti-inflammatory activity through inhibition of NO, IL-1β, IL-6 and anti-proliferation activity through apoptosis induction	[56]
16	Denticulatolide (**183**)	Known	*Sinularia* sp.	Mantanani Island, Sabah	Anti-inflammatory activity through inhibition of NO, IL-1β, IL-6 and anti-proliferation activity through apoptosis induction	[56]
17	Flexibilisin D (**184**)	New	*Sinularia flexibilis*	Off the coast of Liuqiu, Taiwan	Not toxic towards P-388, K-562, and HT-29 cancer cell lines (IC_50_ values > 40 µM) and did not have anti-inflammatory effect through N-formyl-methionyl-leucyl-phenylalanine/cytochalasin B (fMLF-CB)-induced superoxide anion generation and elastase release assay in human neutrophils at concentration of 10 µM	[57]
18	Flexibilisin E (**185**)	New	*Sinularia flexibilis*	Off the coast of Liuqiu, Taiwan	Not toxic towards P-388, K-562, and HT-29 cancer cell lines (IC_50_ values > 40 µM) and did not have anti-inflammatory effect through N-formyl-methionyl-leucyl-phenylalanine/cytochalasin B (fMLF-CB)-induced superoxide anion generation and elastase release assay in human neutrophils at concentration of 10 µM	[57]
19	Flexibilisolide H (**186**)	New	*Sinularia flexibilis*	Off the coast of Liuqiu, Taiwan	Not toxic towards P-388, K-562, and HT-29 cancer cell lines (IC_50_ values > 40 µM) and did not have anti-inflammatory effect through N-formyl-methionyl-leucyl-phenylalanine/cytochalasin B (fMLF-CB)-induced superoxide anion generation and elastase release assay in human neutrophils at concentration of 10 µM	[57]
20	11-Dehydrosinulariolide (**187**)	Known	*Sinularia flexibilis*	Off the coast of Liuqiu, Taiwan	Anti-proliferation activity against P388, K562, HT29 cancer cell line with IC_50_ values of 9.3, 23.4, and 15.9 μM, respectively	[57]
21				Off the coast of Yalong Bay, Hainan, China	Broad anti-proliferation activity against A549, HT-29, SNU-398, and Capan-1 human tumor cell lines with IC_50_ values of 27.4, 22.7, 8.9, and 9.4 µM, respectively	[58]
22	11-*epi*-Sinulariolide acetate (**188**)	Known	*Sinularia flexibilis*	Off the coast of Liuqiu, Taiwan	Anti-proliferation activity against P388, K562, HT29 cancer cell line with IC_50_ values of 6.9, 12.2, and 9.6 μM, respectively	[57]
23				Off the coast of Yalong Bay, Hainan, China	High anti-inflammatory activity through inhibition levels of TNF-α with IC_50_ value of 2.7 µM. Moderate anti-proliferation activities against HT-29, SNU-398, and Capan-1 with IC_50_ values ranging from 24.9 to 32.6 µM	[58]
24	(*S*)-14-Deoxycrassin (**189**)	Known	*Sinularia flexibilis*	Off the coast of Liuqiu, Taiwan	Anti-proliferation activity against P388 and K562 cancer cell line with IC_50_ values of 16.0 and 26.7 μM, respectively. Anti-inflammatory activity through inhibition of superoxide anion generation and elastase release	[57]
25				Off the coast of Liuqiu, Taiwan	Anti-proliferation activity against K562 and HT29 cancer cell line with IC_50_ values of 21.7 and 27.1 μM, respectively	[57]
26	Sinulariolide (**190**)	Known	*Sinularia flexibilis*	Off the coast of Yalong Bay, Hainan, China	Low anti-inflammatory activity through inhibition levels of TNF-α with IC_50_ value of 4.7 µM. Moderate anti-proliferation activities against HT-29, SNU-398, and Capan-1 with IC_50_ values ranging from 24.7 to 33.6 µM	[58]
27			*Sinularia scabra*	Off the coast of Xigu Island, Hainan Province, China	Significant inhibitory effects on the proliferation of LPS induced B lymphocyte cells with IC_50_ value of 9.2 µM	[52]
28	Sandensolide (**191**)	Known	*Sinularia flexibilis*	National Museum of Marine Biology & Aquarium, Pingtung, Taiwan	Anti-oral cancer activity by inducing oxidative stress-mediated cell death pathways through suppressing colony formation, inducing apoptosis, cell cycle arrest, induction of reactive oxygen species (ROS) and was observed in in vitro cultured human OSCC models (Ca9.22, SCC9 and HSC-3 cell lines)	[59]
29	Sinulin C (**192**)	New	*Sinularia* sp.	Yongxing Island of Xisha Islands in the South China Sea	Not cytotoxic against HeLa, HCT-116, and A549 tumour cell lines and did not have inhibitory activity against PTP1B	[54]
30	Sinulin D (**193**)	New	*Sinularia* sp.	Yongxing Island of Xisha Islands in the South China Sea	Mild inhibitory activity against PTP1B with IC_50_ value of 47.5 mM (with sodium orthovanadate as positive control, IC_50_ 881 μM)	[54]
31	(1*R*,3*S*,4*S*,7*E*,11*E*)-3,4-Epoxycembra-7,11,15-triene (**194**)	Known	*Sinularia* sp.	Yongxing Island of Xisha Islands in the South China Sea	Mild inhibitory activity against PTP1B with IC_50_ value of 12.5 mM (with sodium orthovanadate as positive control, IC_50_ 881 μM)	[54]
32	Xidaosinularide A (**195**)	New	*Sinularia flexibilis*	Off the coast of Yalong Bay, Hainan, China	Low anti-inflammatory activity through inhibition levels of TNF-α with IC_50_ value of 20.7 µM	[58]
33	Xidaosinularide B (**196**)	New	*Sinularia flexibilis*	Off the coast of Yalong Bay, Hainan, China	Low anti-inflammatory activity through inhibition levels of TNF-α with IC_50_ value of 38.9 µM	[58]
34	Xidaosinularide C (**197**)	New	*Sinularia flexibilis*	Off the coast of Yalong Bay, Hainan, China	Very low anti-inflammatory activity through inhibition levels of TNF-α with IC_50_ value > 50 µM	[58]
35	Sinuladiterpene I (**198**)	Known	*Sinularia flexibilis*	Off the coast of Yalong Bay, Hainan, China	Moderate anti-inflammatory activity through inhibition levels of TNF-α with IC_50_ value of 13.3 µM	[58]
36	Flexilarin B (**199**)	Known	*Sinularia flexibilis*	Off the coast of Yalong Bay, Hainan, China	Low anti-inflammatory activity through inhibition levels of TNF-α with IC_50_ value of 4.2 µM	[58]
37	1*E*,3*E*,7*E*,-11-hydroxy-12-methoxy-1-isopropyl-4,8,12-trimethyl-icyclotetradeca-1,3,7-triene (**200**)	New	*Sinularia* sp.	Xisha Islands, South China Sea, China	Moderate inhibitory activity against Aß_42_ aggregation with percent inhibition of 20.6% at 10 µM (showed equal potency than the positive control curcumin (20.5%))	[60]
38	3*E*,7*E*-11-hydroxy-12-methoxy-1-isopropenyl-4,8,12-trimethyl-icyclo tetradeca-3,7-diene (**201**)	New	*Sinularia* sp.	Xisha Islands, South China Sea, China	Showed no potent activity against Aß_42_ aggregation inhibition (2.1%) and no cytotoxicity against human tumor cell lines (SH-SY5Y, MDA-MB-426, A549, Hep3B, and HT-29) with proliferation inhibitory rate < 50% at concentration of 10 and 100 µM, respectively	[60]
39	1*E*,3*Z*,7*E*,-11-hydroxy-12- methoxy-1-isopropyl-4,8,12-trimethyl-icyclotetradeca-1,3,7-triene (**202**)	New	*Sinularia* sp.	Xisha Islands, South China Sea, China	Moderate inhibitory activity against Aß_42_ aggregation with percent inhibition of 37.2% at 10 µM (showed higher potency than the positive control curcumin (20.5%))	[60]
40	1*Z*,3*Z*,7*E*,-11-hydroxy-12-methoxy-1-isopropyl-4,8,12-trimethyl-icyclotetradeca-1,3,7-triene (**203**)	New	*Sinularia* sp.	Xisha Islands, South China Sea, China	Showed no potent activity against Aß_42_ aggregation inhibition (1.5%) and no cytotoxicity against human tumour cell lines (SH-SY5Y, MDA-MB-426, A549, Hep3B, and HT-29) with proliferation inhibitory rate < 50% at concentration of 10 and 100 µM, respectively	[60]
41	Xiguscabrate A (**204**)	New	*Sinularia scabra*	Off the coast of Xigu Island, Hainan Province, China	No inhibitory activity on the proliferation of Con A-induced T lymphocyte cells with IC_50_ values > 50 µM	[52]
42	Xiguscabrate B (**205**)	New	*Sinularia scabra*	Off the coast of Xigu Island, Hainan Province, China	Strong inhibitory activity on the proliferation of Con A-induced T lymphocyte cells with IC_50_ value of 8.4 µM	[52]
43	Xiguscabral A (**206**)	New	*Sinularia scabra*	Off the coast of Xigu Island, Hainan Province, China	No inhibitory activity on the proliferation of Con A-induced T lymphocyte cells with IC_50_ values of 15.8 µM	[52]
44	Xiguscabrol A (**207**)	New	*Sinularia scabra*	Off the coast of Xigu Island, Hainan Province, China	Strong inhibitory activity on the proliferation of Con A-induced T lymphocyte cells with IC_50_ value of 5.5 µM	[52]
45	Xiguscabrol B (**208**)	New	*Sinularia scabra*	Off the coast of Xigu Island, Hainan Province, China	Strong inhibitory activity on the proliferation of Con A-induced T lymphocyte cells with IC_50_ value 3.9 µM	[52]
46	8-*epi*-Xiguscabrol B (**209**)	New	*Sinularia scabra*	Off the coast of Xigu Island, Hainan Province, China	Strong inhibitory activity on the proliferation of Con A-induced T lymphocyte cells with IC_50_ value of 2.3 µM	[52]
47	(2*R*,11*S*,12*S*)-Isosarco phytoxide (**210**)	Known	*Sinularia scabra*	Off the coast of Xigu Island, Hainan Province, China	Considerable specific inhibition on B cell proliferation, with IC_50_ value of 4.4 µM and selectivity index (SI) of 10.9, much better than the positive control CsA (SI = 3.0). It dose-dependently inhibited CD19^+^ B cells proliferation by LPS induction. **180** also showed modulatory effects on cytokines production, with the manifestation of decreased IL-6 production and slightly increased IL-10 production. **180** could suppress the derivational expression of CD86 on CD19^+^ B cells upon LPS stimulation. In vitro, LPS addition led to B cells growth and plasma cells formation (from 2.31% to 11.0%) and compound **180** dose-dependently inhibited the percentage of plasma cells	[52]
48	( −)-14-Deoxycrassin (**211**)	Known	*Sinularia scabra*	Off the coast of Xigu Island, Hainan Province, China	Strong inhibitory activity on the proliferation of Con A-induced T lymphocyte cells with IC_50_ value of 6.1 µM	[52]
49	Sinulacrassin A (**212**)	New	*Sinularia crassa*	West Island, South China Sea	Compound not tested	[61]
50	Sinulacrassin B (**213**)	New	*Sinularia crassa*	West Island, South China Sea	Inhibitory effect toward α-Glucosidase with IC_50_ value of 10.65 ± 0.16 μM; not toxic against LO2 cells with IC_50_ > 100 μM	[61]
51	Sinulacrassin C (**214**)	New	*Sinularia crassa*	West Island, South China Sea	No inhibitory effect toward α-Glucosidase	[61]
52	*ent*-Xishaflavalin G (**215**)	New	*Sinularia crasa*	West Island, South China Sea	No inhibitory effect toward α-Glucosidase	[61]
53	S-( +)-Cembrane A (**216**)	Known	*Sinularia crassa*	West Island, South China Sea	Inhibitory effect toward α-Glucosidase with IC_50_ value of 30.31 ± 1.22 μM; not toxic against LO2 cells with IC_50_ > 100 μM	[61]
54	Humilisin A (**217**)	New	*Sinularia humilis*	Ximao Islands, Hainan, China	No anti-inflammatory effects in LPS-stimulated BV-2 microglial cells	[62]
55	Humilisin B (**218**)	New	*Sinularia humilis*	Ximao Islands, Hainan, China	No anti-inflammatory effects in LPS-stimulated BV-2 microglial cells	[62]
56	Humilisin C (**219**)	New	*Sinularia humilis*	Ximao Islands, Hainan, China	No anti-inflammatory effects in LPS-stimulated BV-2 microglial cells	[62]
57	Humilisin D (**220**)	New	*Sinularia humilis*	Ximao Islands, Hainan, China	No anti-inflammatory effects in LPS-stimulated BV-2 microglial cells	[62]
58	Humilisin E (**221**)	New	*Sinularia humilis*	Ximao Islands, Hainan, China	No anti-inflammatory effects in LPS-stimulated BV-2 microglial cells	[62]
59	Humilisin F (**222**)	New	*Sinularia humilis*	Ximao Islands, Hainan, China	Significant anti-inflammatory effects in LPS-stimulated BV-2 microglial cells with 83.96% ± 2.02% and 65.70% ± 2.76% NO level decrease at 10 and 20 μM, respectively; low toxicity toward BV-2 microglial cells	[62]

A known cembrane, sandensolide **191** was isolated from aquacultured S. *flexibilis* in Pingtung, Taiwan [59]. Qin et al. isolated two new and two known compounds from Chinese *Sinularia* sp., named sinulins C-D **192–193** and 5-*epi*-sinuleptolide **178**, (1R,3S,4S,7E,11E)-3,4-epoxycembra-7,11,15-triene **194**, with **192** being reported as not showing any biological activity as tested (Table 2, entries 29–31) [54]. Eight cembranoids were isolated from Chinese *S*. *flexibilis,* three of which were newly discovered. The three new compounds were categorized as polyoxygenated cembranoids (or flexibilide-like cembranoids) and named xidaosinularides A-C **165–167**. The known compounds were categorized as polyoxygenated cembranoids and included 11-dehydrosinulariolide **187**, 11-*epi*-sinulariolide acetate **188**, sinulariolide **190**, sinuladiterpene I **198**, and flexilarin B **199** [58] Tables 3 and 4.

**Table 3:** The biological activities of cembranoid isolates from genera *Lobophytum*

Entry	Compound name (number)	Novelty	Sources	Geographical area of collection	Biological activities	References
1	Cembrene A (**223**)	New	*Lobophytum* sp.	Off the Saudi Arabia Red Sea Coast at Jeddah	Moderate anti-bacterial activity with inhibition zone diameter of 11–15 mm and MIC value of 30 μg/mL. Significant toxicity against *A. salina* with LD_50_ value of 25 μg/mL and significant anti-tumor activity against Ehrlich carcinoma cells with LD_50_ value of 50 μg/mL	[63]
2	Locrassumin A (**224**)	New	*Lobophytum crassum*	Inner coral reef of Meishan, Hainan Province, China	Moderate inhibition against LPS-induced NO production with IC_50_ value of 17 ± 3 μM	[64]
3	Locrassumin B (**225**)	New	*Lobophytum crassum*	Inner coral reef of Meishan, Hainan Province, China	Compound not tested	[64]
4	Locrassumin C (**226**)	New	*Lobophytum crassum*	Inner coral reef of Meishan, Hainan Province, China	Compound not tested	[64]
5	Locrassumin D (**227**)	New	*Lobophytum crassum*	Inner coral reef of Meishan, Hainan Province, China	Compound not tested	[64]
6	Locrassumin E (**228**)	New	*Lobophytum crassum*	Inner coral reef of Meishan, Hainan Province, China	Compound not tested	[64]
7	Locrassumin F (**229**)	New	*Lobophytum crassum*	Inner coral reef of Meishan, Hainan Province, China	Compound not tested	[64]
8	Locrassumin G (**230**)	New	*Lobophytum crassum*	Inner coral reef of Meishan, Hainan Province, China	Moderate inhibition against LPS-induced NO production with IC_50_ value of 13 ± 2 μM	[64]
9	(−)-Laevigatol B (**231**)	New	*Lobophytum crassum*	Inner coral reef of Meishan, Hainan Province, China	Compound not tested	[64]
10	(−)-Isosarcophine (**232**)	New	*Lobophytum crassum*	Inner coral reef of Meishan, Hainan Province, China	Compound not tested	[64]
11	(−)-*7R,8S*-Dihydroxydeepoxy sarcophytoxide (**233**)	New	*Lobophytum crassum*	Inner coral reef of Meishan, Hainan Province, China	Compound not tested	[64]
*12*	*ent*-Sarcophine (**234**)	Known	*Lobophytum crassum*	Inner coral reef of Meishan, Hainan Province, China	Moderate inhibition against LPS-induced NO production with IC_50_ value of 24 ± 2 μM	[64]
13	Sarcophytonolide O (**235**)	Known	*Lobophytum crassum*	Inner coral reef of Meishan, Hainan Province, China	Moderate inhibition against LPS-induced NO production with IC_50_ value of 8 ± 1 μM	[64]
14	Ketoemblide (**236**)	Known	*Lobophytum crassum*	Inner coral reef of Meishan, Hainan Province, China	Moderate inhibition against LPS-induced NO production with IC_50_ value of 12 ± 2 μM	[64]
15	Lobophylin F (**237**)	New	*Lobophytum crassum*	Off the coast of Dongsha Atoll	Compound not tested	[65]
16	Lobophylin G (**238**)	New	*Lobophytum crassum*	Off the coast of Dongsha Atoll	Compound not tested	[65]
17	Lobophylin H (**239**)	New	*Lobophytum crassum*	Off the coast of Dongsha Atoll	Compound not tested	[65]
18	Compound 1 (**240**)	New	*Lobophytum* sp.	Coast of Irabu Island, Okinawa, Japan	Weak anti-bacterial activity with 10 mm inhibiton zone against S. *aureus* and E. *coli* at 25 μg/disc. Mild cytotoxicity against HCT116 with IC_50_ value of 135.37 μM. Anti-inflammatory activity through reducing NO production with IC_50_ value of 41.21 μM	[66]
19	Compound 2 (**241**)	New	*Lobophytum* sp.	Coast of Irabu Island, Okinawa, Japan	Weak anti-bacterial activity with 9 mm inhibiton zone against S. *aureus* and 10 mm against E. *coli* at 25 μg/disc. Mild cytotoxicity against HCT116 with IC_50_ value of 177.11 μM. Anti-inflammatory activity through reducing NO production with IC_50_ value of 64.96 μM	[66]
20	Compound 3 (**242**)	New	*Lobophytum* sp.	Coast of Irabu Island, Okinawa, Japan	Weak anti-bacterial activity with 9 mm inhibiton zone against S. *aureus* and 10 mm against E. *coli* at 25 μg/disc. Mild cytotoxicity against HCT116 with IC_50_ value of 153.11 μM. Anti-inflammatory activity through reducing NO production with IC_50_ value of 74.76 μM	[66]
21	Grandilobatin B (**243**)	Known	*Lobophytum* sp.	Coast of Irabu Island, Okinawa, Japan	Anti-bacterial activity with 10 mm inhibiton zone against S. *aureus* and 12 mm against E. *coli* at 25 μg/disc	[66]
22	Sinugibberol (**244**)	Known	*Lobophytum* sp.	Coast of Irabu Island, Okinawa, Japan	Anti-bacterial activity with 10 mm inhibiton zone against S. *aureus* and 15 mm against E. *coli* at 25 μg/disc	[66]
23	Lobophyolide A (**245**)	New	*Lobophytum crassum*	Off the coast of Pingtung, Taiwan	Potent anti-inflammatory activity through inhibition of LPS induced IL-12 release by DC 93.4 ± 0.5% and inhibition of LPS induced NO release by DC 93.5 ± 6.5% DC survival 76.0 ± 0.01%	[11]
24	Lobophyolide B (**246**)	New	*Lobophytum crassum*	Off the coast of Pingtung, Taiwan	Anti-inflammatory activity through inhibition of LPS induced IL-12 release by DC 93.6 ± 0.0% and inhibition of LPS induced NO release by DC 95.9 ± 3.2% DC survival 52.0 ± 0.04%	[11]
25	16-Methoxycarbonyl cembrene A (**247**)	Known	*Lobophytum crassum*	Off the coast of Pingtung, Taiwan	Anti-inflammatory activity through inhibition of LPS induced IL-12 release by DC 86.3 ± 1.1% and inhibition of LPS induced NO release by DC 86.1 ± 2.2% DC survival 75.0 ± 0.01%	[11]
26	Sinarone (**248**)	Known	*Lobophytum crassum*	Off the coast of Pingtung, Taiwan	Potent anti-inflammatory activity through inhibition of LPS induced IL-12 release by DC 77.0 ± 1.5% and inhibition of LPS induced NO release by DC 54.9 ± 0.50% DC survival 85.0 ± 0.08%	[11]
27	Sinaluriol D (**249**)	Known	*Lobophytum crassum*	Off the coast of Pingtung, Taiwan	Potent anti-inflammatory activity through inhibition of LPS induced IL-12 release by DC 86.4 ± 0.0% and inhibition of LPS induced NO release by DC 86.1 ± 3.0% DC survival 85.0 ± 5.00%	[11]
28	Culobophylin D (**250**)	New	*Lobophytum crassum*	Collected from the coast of Pingtung, Taiwan, then were preserved and aquacultured in National Museum of Marine Biology & Aquarium (Pingtung, Taiwan)	Inactive at cytotoxicity test against leukemia cell lines (Molt 4, K562, U937, and Sup-T1)	[67]
29	Culobophylin E (**251**)	New	*Lobophytum crassum*	Collected from the coast of Pingtung, Taiwan, then were preserved and aquacultured in National Museum of Marine Biology & Aquarium (Pingtung, Taiwan)	Compound not tested	[67]
30	Lobocrassin C (**252**)	Known	*Lobophytum crassum*	Collected from the coast of Pingtung, Taiwan, then were preserved and aquacultured in National Museum of Marine Biology & Aquarium (Pingtung, Taiwan)	Anti-proliferation activity against Sup-T1 cell line with IC_50_ of 35.8 μM	[67]
31	Lobophylin (**253**)	Known	*Lobophytum crassum*	Collected from the coast of Pingtung, Taiwan, then were preserved and aquacultured in National Museum of Marine Biology & Aquarium (Pingtung, Taiwan)	Anti-proliferation activity against K562, Molt 4, Sup-T1 with IC_50_ values of 16.3, 12.3, and 4.6 μM, respectively	[67]
32	Crassocolide E (**254**)	Known	*Lobophytum crassum*	Collected from the coast of Pingtung, Taiwan, then were preserved and aquacultured in National Museum of Marine Biology & Aquarium (Pingtung, Taiwan)	Anti-proliferation activity against K562, Molt 4, U937, and Sup-T1 with IC_50_ values of 11.3, 6.2, 15.8, and 5.2 μM, respectively	[67]
33	Sarcocrassocolide (**255**)	Known	*Lobophytum crassum*	Collected from the coast of Pingtung, Taiwan, then were preserved and aquacultured in National Museum of Marine Biology & Aquarium (Pingtung, Taiwan)	Antiproliferation activity against K562, Molt 4, U937, and Sup-T1 with IC_50_ values of 18.1, 8.4, 4.4, and 8.3 μM, respectively	[67]
34		Known	*Lobophytum crassum*	Collected from the coast of Pingtung, Taiwan, then were preserved and aquacultured in National Museum of Marine Biology & Aquarium (Pingtung, Taiwan)	Anti-proliferation activity against K562, Molt 4, U937, and Sup-T1 with IC_50_ values of 3.3, 1.2, 7.1, and 1.5 μM, respectively	[53]
35	13-Acetoxysarcocrassocolide (**256**)		*Lobophytum crassum*	Collected from the coast of Pingtung, Taiwan, then were preserved and aquacultured in National Museum of Marine Biology & Aquarium (Pingtung, Taiwan)	Exerted its cytotoxic activity in oral cancer cells Ca9-22 through the promotion of ROS generation and the suppression of the anti-oxidant enzyme activity. The apoptotic effect was found to be mediated through the interruption of the Keap1/Nrf2/p62/SQSTM1 pathway. It increased the expression of apoptosis- and DNA damage-related proteins in a concentration- and time-dependent manner. It exerted potent anti-tumor effect against oral cancer cells, as demonstrated by the in vivo xenograft animal model. It significantly reduced the tumor volume (55.29%) and tumor weight (90.33%)	[54]
36	Sarocrassocolide M (**257**)	Known	*Lobophytum crassum*	Collected from the coast of Pingtung, Taiwan, then were preserved and aquacultured in National Museum of Marine Biology & Aquarium (Pingtung, Taiwan)	Anti-proliferation activity against K562, Molt 4, U937, and Sup-T1 with IC_50_ values of 15.3, 11.6, 32.0, and 10.2 μM, respectively	[67]
37	(*R*)-14-deoxycrassin (**258**)	Known	*Lobophytum crassum*	Collected from the coast of Pingtung, Taiwan, then were preserved and aquacultured in National Museum of Marine Biology & Aquarium (Pingtung, Taiwan)	Anti-proliferation activity against K562, Molt 4, U937, and Sup-T1 with IC_50_ values of 4.5, 2.9, 7.0, and 4.5 μM, respectively	[67]
38	Lobocrassin B (**259**)	Known	*Lobophytum crassum*	Collected from the coast of Pingtung, Taiwan, then were preserved and aquacultured in National Museum of Marine Biology & Aquarium (Pingtung, Taiwan)	Anti-proliferation activity against K562, Molt 4, U937, Sup-T1 with IC_50_ values of 3.3, 2.3, 5.2, and 6.2 μM, respectively	[67]
39	Sarcocrassocolide F (**260**)	Known	*Lobophytum crassum*	Collected from the coast of Pingtung, Taiwan, then were preserved and aquacultured in National Museum of Marine Biology & Aquarium (Pingtung, Taiwan)	Anti-proliferation activity against K562, Molt 4, U937, and Sup-T1 with IC_50_ values of 12.3, 4.8, 10.9, 6.1 μM, respectively	[67]
40	Sarcocrassocolide G (**261**)	Known	*Lobophytum crassum*	Collected from the coast of Pingtung, Taiwan, then were preserved and aquacultured in National Museum of Marine Biology & Aquarium (Pingtung, Taiwan)	Anti-proliferation activity against K562, Molt 4, U937, and Sup-T1 with IC_50_ values of 13.0, 7.0, 23.3, 6.6 μM, respectively	[67]
41	Compound 4 (**262**)	New	*Lobophytum* sp.	Coast of Irabu Island, Okinawa, Japan	Moderate anti-proliferation activity against HeLa, A459, B16-F10, and RAW 264.7 cells with IC_50_ of 7.81, 9.30, 10.83, and 5.99 μM, respectively. Anti-inflammatory effect through suppression of NO production in a dose-dependent manner with IC_50_ of 10.67 µM (at 24 h) in LPS-stimulated RAW 264.7 macrophage cells at non-cytotoxic concentrations	[69]
42	Compound 5 (**263**)	New	*Lobophytum* sp.	Coast of Irabu Island, Okinawa, Japan	Low anti-proliferation activity against HeLa, A459, and RAW 264.7 cells with IC_50_ of 49.33, 54.09, and 43.74 μM, respectively. Anti-inflammatory effect through suppression of NO production in a dose-dependent manner with IC_50_ of 13.92 µM (at 24 h) in LPS-stimulated RAW 264.7 macrophage cells at non-cytotoxic concentrations	[69]
43	Compound 6 (**264**)	New	*Lobophytum* sp.	Coast of Irabu Island, Okinawa, Japan	Low anti-proliferation activity against RAW 264.7 cells with IC_50_ of 45.22. Anti-inflammatory effect through suppression of NO production in a dose-dependent manner with IC_50_ of 14.02 µM (at 24 h) in LPS-stimulated RAW 264.7 macrophage cells at non-cytotoxic concentrations	[69]
44	Lobophytrol A (**265**)	New	*Lobophytum* sp*.*	Off the coast of Weizhou Island, Guangxi Autonomous Region, China	Showed no effects on anti-inflammatory and immunological activity assay	[70]
45	Lobophytrol B (**266**)	New	*Lobophytum* sp.	Off the coast of Weizhou Island, Guangxi Autonomous Region, China	Showed no effects on anti-inflammatory and immunological activity assay	[70]
46	Lobophytrol C (**267**)	New	*Lobophytum* sp.	Off the coast of Weizhou Island, Guangxi Autonomous Region, China	Showed no effects on anti-inflammatory and immunological activity assay	[70]
47	Lobophytolin A (**268**)	New	*Lobophytum* sp.	Off the coast of Xisha Islands, Hainan Province	Inactive at a concentration of 10 µM, on the HT-29, Capan-1, A549, and SNU-398 tumor cell lines (showed IC_50_ > 50 µM)	[71]
48	Lobophytolin B (**269**)	New	*Lobophytum* sp.	Off the coast of Xisha Islands, Hainan Province	Inactive at a concentration of 10 µM, on the HT-29, Capan-1, A549, and SNU-398 tumor cell lines (IC_50_ values ranging from 30 to 40 µM)	[71]
49	Lobophytolin C (**270**)	New	*Lobophytum sp.*	Xisha Island, Hainan, China	Moderate cytotoxicity against SNU-398 with IC_50_ value of 42.54 ± 6.26 μM; weak inhibitory effect of XBP-Splicing on B16-F10 tumor cells at 10 μM	[72]
50	Lobophytolin D (**271**)	New	*Lobophytum sp.*	Xisha Island, Hainan, China	Cytotoxic against HT-29, Capan-1, A549, and SNU-398 with IC_50_ values of 4.52 ± 0.82; 6.62 ± 4.02; 5.17 ± 0.86; 6.15 ± 2.28 μM, respectively; weak inhibitory effect of XBP-Splicing on B16-F10 tumor cells at 10 μM	[72]
51	Lobophytolin E (**272**)	New	*Lobophytum sp.*	Xisha Island, Hainan, China	Not cytotoxic against HT-29, Capan-1, A549, and SNU-398; weak inhibitory effect of XBP-Splicing on B16-F10 tumor cells at 10 μM	[72]
52	Lobophytolin F (**273**)	New	*Lobophytum sp.*	Xisha Island, Hainan, China	Not cytotoxic against HT-29, Capan-1, A549, and SNU-398; weak inhibitory effect of XBP-Splicing on B16-F10 tumor cells at 10 μM	[72]
53	Lobophytolin G (**274**)	New	*Lobophytum sp.*	Xisha Island, Hainan, China	Not cytotoxic against HT-29, Capan-1, A549, and SNU-398; weak inhibitory effect of XBP-Splicing on B16-F10 tumor cells at 10 μM	[72]
54	Lobophytolin H (**275**)	New	*Lobophytum sp.*	Xisha Island, Hainan, China	Not cytotoxic against HT-29, Capan-1, A549, and SNU-398; weak inhibitory effect of XBP-Splicing on B16-F10 tumor cells at 10 μM	[72]
55	Lobophytolin I (**276**)	New	*Lobophytum sp.*	Xisha Island, Hainan, China	Not cytotoxic against HT-29, Capan-1, A549, and SNU-398; weak inhibitory effect of XBP-Splicing on B16-F10 tumor cells at 10 μM	[72]

**Table 4:** The biological activities of cembranoid isolates from other soft coral species

Entry	Compound name (number)	Novelty	Sources	Geographical area of collection	Biological activities	Refs.
1	Claudieunicellin S (**277**)	Known	*Cladiella tuberculosa*	Off the Penghu Archipelago waters, Taiwan	Moderate anti-proliferation activity against MOLT-4, K562, SUP-T1 with IC_50_ values of 6.04, 6.80, 6.90 μg/mL, respectively	[73]
2	Briarenolide ZI (**278**)	New	*Briareum* sp.	Off the coast of southern Taiwan	Inactive on iNOS level assay and cytotoxicity assay against RAW 264.7	[74]
3	Briarenolide ZII (**279**)	New	*Briareum* sp.	Off the coast of southern Taiwan	Anti-inflammatory activity through reducing iNOS level to 47.2% at a concentration of 10 μM	[74]
4	Briarenolide ZIII (**280**)	New	*Briareum* sp.	Off the coast of southern Taiwan	Inactive on iNOS level assay and cytotoxicity assay against RAW 264.7	[74]
5	Briarenolide ZIV (**281**)	New	*Briareum* sp.	Off the coast of southern Taiwan	Inactive on iNOS level assay and cytotoxicity assay against RAW 264.7	[74]
6	Briarenolide ZV (**282**)	New	*Briareum* sp.	Off the coast of southern Taiwan	Inactive on iNOS level assay and cytotoxicity assay against RAW 264.7	[74]
7	Briarenolide ZVI (**283**)	New	*Briareum* sp.	Off the coast of southern Taiwan	Anti-inflammatory activity through reducing iNOS level to 55.7% at a concentration of 10 μM	[74]
8	10-Hydroxy-nephthenol acetate (**284**)	New	*Nephthea* sp.	Layangan, Sabah	Anti-bacterial activity against S. *aureus* and E. *coli* with MBC of 180 and 75 μg/mL, respectively. Anti-proliferation activity against HeLa and MCF-7 with IC_50_ values of 40 and 25 μg/mL, respectively	[75]
9	7,8-Epoxy-10-hydroxy-nephthenol acetate (**285**)	New	*Nephthea* sp.	Layangan, Sabah	Anti-bacterial activity against S. *aureus* and E. *coli* with MBC of 150 and 75 μg/mL, respectively. Anti-proliferation activity against HeLa and MCF-7 with IC_50_ values of 125 and 75 μg/mL, respectively	[75]
10	6-Acetoxy-7,8-epoxy-10-hydroxy-nephthenol acetate (**286**)	New	*Nephthea* sp.	Layangan, Sabah	Compound not tested	[75]
11	3-Deacetylpraelolide (**287**)	New	*Junceella fragilis*	Inner coral reef in Hainan Island of China	Anti-inflammatory activity through inhibition of NO production with % inhibition of 39.4 ± 1.2% (at 50 μM) in RAW 264.7 cell	[76]
12	13-α-Acetoxyl-3-deacetylpraelolide (**288**)	New	*Junceella fragilis*	Inner coral reef in Hainan Island of China	Anti-inflammatory activity through inhibition of NO production with % inhibition of 42.7 ± 1.4% (at 50 μM) in RAW 264.7 cell	[76]
13	13-α-Acetoxyl-2-deacetylpraelolide (**289**)	New	*Junceella fragilis*	Inner coral reef in Hainan Island of China		
14	13-α-Acetoxyl-3-deacetyljunceellin (**290**)	New	*Junceella fragilis*	Inner coral reef in Hainan Island of China	Anti-inflammatory activity through inhibition of NO production with % inhibition of 36.3 ± 0.6% (at 50 μM) in RAW 264.7 cell	[76]
15	13-α-Acetoxyl-2-deacetyljunceellin (**291**)	New	*Junceella fragilis*	Inner coral reef in Hainan Island of China		
16	Klyflaccicembranol A (**292**)	New	*Klyxum flaccidum*	Off the coast of Hsiao Liuchiu Island (Pingtung County), along the coast of the island of Pratas, Taiwan	Weak anti-inflammatory activity through NO inhibitory activity with % inhibition of 25%	[77]
17	Klyflaccicembranol B (**293**)	New	*Klyxum flaccidum*	Off the coast of Hsiao Liuchiu Island (Pingtung County), along the coast of the island of Pratas, Taiwan	Anti-proliferation activity against A549 and K562 with IC_50_ values of 16.5 and 34.6 μM, respectively	[77]
18	Klyflaccicembranol C (**294**)	New	*Klyxum flaccidum*	Off the coast of Hsiao Liuchiu Island (Pingtung County), along the coast of the island of Pratas, Taiwan	Weak anti-inflammatory activity through NO inhibitory activity with % inhibition of 12%	[77]
19	Klyflaccicembranol D (**295**)	New	*Klyxum flaccidum*	Off the coast of Hsiao Liuchiu Island (Pingtung County), along the coast of the island of Pratas, Taiwan	Anti-proliferation activity against K562 with IC_50_ values of 44.9 μM. Moderate anti-inflammatory activity through NO inhibition to 65% with IC_50_ value of 46.7 μg/mL	[77]
20	Klyflaccicembranol E (**296**)	New	*Klyxum flaccidum*	Off the coast of Hsiao Liuchiu Island (Pingtung County), along the coast of the island of Pratas, Taiwan	Strong anti-inflammatory activity through NO inhibition to 88% at concentration of 50 μg/mL	[77]
21	Klyflaccicembranol F (**297**)	New	*Klyxum flaccidum*	Off the coast of Hsiao Liuchiu Island (Pingtung County), along the coast of the island of Pratas, Taiwan	Anti-proliferation activity against A549 with IC_50_ values of 21.4 μM. Moderate anti-inflammatory activity through NO inhibition to 64% with IC_50_ value of 47.0 μg/mL	[77]
22	Klyflaccicembranol G (**298**)	New	*Klyxum flaccidum*	Off the coast of Hsiao Liuchiu Island (Pingtung County), along the coast of the island of Pratas, Taiwan	Compound not tested	[77]
23	Klyflaccicembranol H (**299**)	New	*Klyxum flaccidum*	Off the coast of Hsiao Liuchiu Island (Pingtung County), along the coast of the island of Pratas, Taiwan	Anti-proliferation activity against A549, K652, and P388 with IC_50_ values of 49.4, 47.4, and 34.6 μM, respectively. Weak anti-inflammatory activity through NO inhibitory activity with % inhibition of 20%	[77]
24	Klyflaccicembranol I (**300**)	New	*Klyxum flaccidum*	Off the coast of Hsiao Liuchiu Island (Pingtung County), along the coast of the island of Pratas, Taiwan	Anti-proliferation activity against HT-29 with IC_50_ values of 41.9 μM. Strong anti-inflammatory activity through NO inhibition to 87% at concentration of 50 μg/mL	[77]
25	Gibberosene D (**301**)	Known	*Klyxum flaccidum*	Off the coast of Hsiao Liuchiu Island (Pingtung County), along the coast of the island of Pratas, Taiwan	Weak anti-inflammatory activity through NO inhibitory activity with % inhibition of 15%	[77]
26	(3*E*,6*E*,10*E*)-8a-butoxy-17 (15 → 14), 20(12 → 11)- bis-abeo-cembra-3,6,10, 14(17),15-pentaene (**302**)	New	*Chicoreus ramosus*	Fishing harbors of Tuticorin located along the south-east coastlines of Tamil Nadu in Gulf of Mannar area, which were located between Sri Lanka and India	Anti-oxidant activity through DPPH and ABTS^+^ scavenging activity with IC_50_ values of 0.26 and 0.36 mg/mL, respectively. Anti-inflammatory activity through inhibition of 5-lipooxygenase with IC_50_ value of 0.76 mg/mL	[78]
27	Compound 7 (**303**)	New	*Eunicea* sp.	Off Caribbean Sea (Panama)	Compound not tested	[79]
28	Compound 8 (**304**)	New	*Eunicea* sp.	Off Caribbean Sea (Panama)	Improving INS-1 pancreatic beta cell proliferation with ratio of 1.9 ± 0.5 (fold to control)	[79]
29	Compound 9 (**305**)	New	*Eunicea* sp.	Off Caribbean Sea (Panama)	Compound not tested	[79]
30	Compound 10 (**306**)	New	*Eunicea* sp.	Off Caribbean Sea (Panama)	Compound not tested	[79]
31	Compound 11 (**307**)	New	*Eunicea* sp.	Off Caribbean Sea (Panama)	Compound not tested	[79]
32	Compound 12 (**308**)	New	*Eunicea* sp.	Off Caribbean Sea (Panama)	Compound not tested	[79]
33	Compound 13 (**309**)	New	*Eunicea* sp.	Off Caribbean Sea (Panama)	Compound not tested	[79]
34	Compound 14 (**310**)	New	*Eunicea* sp.	Off Caribbean Sea (Panama)	Compound not tested	[79]
35	Compound 15 (**311**)	New	*Eunicea* sp.	Off Caribbean Sea (Panama)	Compound not tested	[79]
36	Euniolide (**312**)	Known	*Eunicea* sp.	Off Caribbean Sea (Panama)	Improving INS-1 pancreatic beta cell proliferation with ratio of 1.7 ± 0.5 (fold to control)	[79]
37	14-Deoxycrassin (**313**)	Known	*Eunicea* sp.	Off Caribbean Sea (Panama)	Improving INS-1 pancreatic beta cell proliferation with ratio of 1.7 ± 0.6 (fold to control)	[79]
38	Pseudoplexauric acid methyl ester (**314**)	Known	*Eunicea* sp.	Off Caribbean Sea (Panama)	Improving INS-1 pancreatic beta cell proliferation with ratio of 2.2 ± 0.6 (fold to control)	[79]
39	(1S*,3S*,4S*,7*E*,11*E*)-3,4- epoxy-13-oxo-7,11,15- cembratriene (**315**)	Known	*Eunicea* sp.	Off Caribbean Sea (Panama)	Improving INS-1 pancreatic beta cell proliferation with ratio of 1.4 ± 0.4 (fold to control)	[79]
40	(-)-Eunicenone (**315**)	Known	*Eunicea* sp.	Off Caribbean Sea (Panama)	Improving INS-1 pancreatic beta cell proliferation with ratio of 1.1 ± 0.1 (fold to control)	[79]
41	Chabrolene (**316**)	New	*Nephtea* sp.	Mantanani Island, Sabah	Repellent activity against *Sitophilus zeamais* at 25 μg/cm^2^	[80]
42	Asperdiol acetate (**317**)	Known	*Pseudoplexaura flagellosa*	Santa Marta Bay, Colombia	Moderate cytotoxicity against PC3 and A549 with IC_50_ of 34.2 and 64.0 μg/mL, respectively	[81]
43	Knightal (**318**)	Known	*Pseudoplexaura flagellosa*	Santa Marta Bay, Colombia	Moderate cytotoxicity against MDA-MB-231, PC3, and L929 cell lines with IC_50_ of 52.7; 54.28; 68.7 μg/mL, respectively	[81]
44	14-Acetoxycrassine (**319**)	Known	*Pseudoplexaura porosa*	Colombian Caribbean Sea	Acetylcholinesterase (AChE) inhibition activity with IC_50_ value of 1.40 ± 0.113 µM, which showed potential to be develop as neurodegenerative diseases treatment, eg. Alzheimer disease	[82]
45	Asperdiol (**320**)	Known	*Eunicea knighti*	Colombian Caribbean Sea	Acetylcholinesterase (AChE) inhibition activity with IC_50_ value of 0.358 ± 0.130 µM, which showed potential to be develop as neurodegenerative diseases treatment, eg. Alzheimer disease	[82]
46	Flaccidodioxide (**321**)	New	*Klyxum flaccidum*	Along the coast of Pratas Island, Taiwan	Low anti-proliferation activity against P388D1 mouse lymphocytic leukemia cell line with IC_50_ of 19.6 μg/mL	[83]
47	Flaccidodiol (**322**)	New	*Klyxum flaccidum*	Along the coast of Pratas Island, Taiwan	Showed no inhibition activity of superoxide anion and elastase at a concentration of 10 µM relative to the control group	[83]
48	14-O-acetylsarcophytol B (**323**)	Known	*Klyxum flaccidum*	Along the coast of Pratas Island, Taiwan	Potent anti-proliferation activity against human lung adenocarcinoma (A549), human colorectal adenocarcinoma (DLD-1), and mouse lymphocytic leukemia (P388D1) cell lines with IC_50_ values of 10.8; 11.7; 8.9 μg/ml, respectively. Anti-inflammatory activity by reducing the level of elastase release to 59.66 ± 0.83% with IC_50_ value of 7.22 ± 0.85 µM, at a concentration of 10 µM relative to the control group	[83]
49	17-*epi*-Junceellolide B (**324**)	New	*Junceella fragilis*	Conco Island, Vietnam	No significant cytotoxic activity against LNCaP, HepG2, KB, MCF-7, SK-Mel2, HL-60, LU-1 and SW480 cancer cell lines (IC_50_ > 100 μM)	[84]
50	Junceellolide B (**325**)	Known	*Junceella fragilis*	Conco Island, Vietnam	Weak cytotoxicity against LNCaP cell line with IC_50_ of 85.34 ± 4.96 μM, relative to that of the positive control ellipticine (IC_50_ 1.42 ± 0.08 μM)	[84]
51	Briaviodiol B (**326**)	New	*Briareum violaceum*	Cultured-type B. *violaceum*, collected from the tank	Anti-inflammatory activity in LPS induced-RAW 264.7 macrophage cells by inhibiting significantly the expression of iNOS protein to 43%	[85]
52	Briaviodiol C (**327**)	New	*Briareum violaceum*	Cultured-type B. *violaceum*, collected from the tank	No in vitro anti-inflammatory activity in LPS induced-RAW 264.7 macrophage cells through expression of iNOS protein at concentration of 10 μM	[85]
53	Briaviodiol D (**328**)	New	*Briareum violaceum*	Cultured-type B. *violaceum*, collected from the tank	Anti-inflammatory activity in LPS induced-RAW 264.7 macrophage cells by inhibiting significantly the expression of iNOS protein to 61%	[85]
54	Briaviodiol E (**329**)	New	*Briareum violaceum*	Cultured-type B. *violaceum*, collected from the tank	Anti-inflammatory activity in LPS induced-RAW 264.7 macrophage cells by inhibiting significantly the expression of iNOS protein to 46%	[85]
55	Fragilide M (**330**)	New	*Junceella fragilis*	Off the coast of Lanyu Island (Orchid Island), Taiwan	Inactive to reduce the level of COX-2 and iNOS in relation to control cells stimulated with LPS only in RAW 264.7 macrophage cells and did not induce cytotoxicity in RAW 264.7 macrophage cells	[86]
56	Fragilide N (**331**)	New	*Junceella fragilis*	Off the coast of Lanyu Island (Orchid Island), Taiwan	Inactive to reduce the level of COX-2 and iNOS in relation to control cells stimulated with LPS only in RAW 264.7 macrophage cells and did not induce cytotoxicity in RAW 264.7 macrophage cells	[86]
57	Fragilide O (**332**)	New	*Junceella fragilis*	Off the coast of Lanyu Island (Orchid Island), Taiwan	Inactive to reduce the level of COX-2 and iNOS in relation to control cells stimulated with LPS only in RAW 264.7 macrophage cells and did not induce cytotoxicity in RAW 264.7 macrophage cells	[86]
58	Erythrolide A (**333**)	Known	*Erythropodium caribaeorum*	Three sites in Providencia Island (SW Caribbean), one in Santa Marta bay, and two sites at Islas del Rosario (near Cartagena)	Anti-proliferation activity against A549, MCF-7 and PC3 cancer cell line with IC_50_ values of 18.41, 6.77 and 2.45 μM, respectively	[87]
59	Erythrolide B (**334**)	Known	*Erythropodium caribaeorum*	Three sites in Providencia Island (SW Caribbean), one in Santa Marta bay, and two sites at Islas del Rosario (near Cartagena)	Anti-proliferation activity against A549, MCF7, and PC3 cancer cell line with IC_50_ values of 27.09, 15.21, and 6.46 μM, respectively	[87]
60	Erythrolide D (**335**)	Known	*Erythropodium caribaeorum*	Three sites in Providencia Island (SW Caribbean), one in Santa Marta bay, and two sites at Islas del Rosario (near Cartagena)	Anti-proliferation activity against A549, MCF7, and PC3 cancer cell line with IC_50_ values of 2.58, 42.45, and 60.00 μM, respectively	[87]
61	Erythrolide F (**336**)	Known	*Erythropodium caribaeorum*	Three sites in Providencia Island (SW Caribbean), one in Santa Marta bay, and two sites at Islas del Rosario (near Cartagena)	Low anti-proliferation activity against A549 cancer cell line with IC_50_ value of 46.49 μM	[87]
62	Erythrolide J (**337**)	Known	*Erythropodium caribaeorum*	Three sites in Providencia Island (SW Caribbean), one in Santa Marta bay, and two sites at Islas del Rosario (near Cartagena)	Anti-proliferation activity against A549, MCF7, and PC3 cancer cell line with IC_50_ values of 37.93, 56.06, and 42.49 μM, respectively	[87]
63	Erythrolide U (**338**)	Known	*Erythropodium caribaeorum*	Three sites in Providencia Island (SW Caribbean), one in Santa Marta bay, and two sites at Islas del Rosario (near Cartagena)	Low anti-proliferation activity against A549 cancer cell line with IC_50_ value of 36.65 μM	[87]
64	Erythrolide W (**339**)	New	*Erythropodium caribaeorum*	Three sites in Providencia Island (SW Caribbean), one in Santa Marta bay, and two sites at Islas del Rosario (near Cartagena)	No cytotoxicity against A549, MCF7, and PC3 cancer cell line with IC_50_ values > 120 μM	[87]
65	Erythrolide X (**340**)	New	*Erythropodium caribaeorum*	Three sites in Providencia Island (SW Caribbean), one in Santa Marta bay, and two sites at Islas del Rosario (near Cartagena)	No cytotoxicity against A549, MCF7, and PC3 cancer cell line with IC_50_ values > 120 μM	[87]
66	Cladieunicellin U (**341**)	New	*Cladiella* sp.	Penghu Archipelago waters, Taiwan	Anti-inflammation activity through decreasing the release of elastase with inhibition rates of 12.01%. Moderate anti-proliferation activity toward the leukemia K562 cells with IC_50_ of 12.76 μg/mL	[88]
67	Cladieunicellin V (**342**)	New	*Cladiella* sp.	Penghu Archipelago waters, Taiwan	Anti-inflammation activity through decreasing the generation of superoxide anions by human neutrophils with inhibition rates of 13.43%. Moderate anti-proliferation activity toward the leukemia MOLT-4 cells with IC_50_ of 18.83 μg/mL	[88]
68	Sclerophytin A (**343**)	Known	*Cladiella* sp.	Penghu Archipelago waters, Taiwan	Anti-inflammation activity through decreasing the release of elastase with inhibition rates of 11.35%	[88]
69	Sclerophytin B (**344**)	Known	*Cladiella* sp.	Penghu Archipelago waters, Taiwan	Anti-inflammation activity through decreasing the release of elastase and superoxide anions with inhibition rates of 16.37% and 28.12%, respectively. Moderate anti-proliferation activity toward the leukemia K562 cells with IC_50_ of 11.39 μg/mL	[88]
70	Briaviodiol F (**345**)	New	*Briareum violaceum*	Cultivation tank at the National Museum of Marine Biology and Aquarium (NMMBA) in Southern Taiwan	No significant cytotoxic effects in RAW 264.7 and showed no suppression effect on iNOS release	[89]
71	Briaviotriol A (**346**)	New	*Briareum violaceum*	Cultivation tank at the National Museum of Marine Biology and Aquarium (NMMBA) in Southern Taiwan	Anti-inflammatory activity by exerted inhibition effects on inducible nitric oxide synthase (iNOS) release from LPS-stimulated RAW 264.7 cells to 67.7%, when compared with results of the cells stimulated with only LPS at concentration of 10 μM	[89]
72	Briaviotriol B (**347**)	New	*Briareum violaceum*	Cultivation tank at the National Museum of Marine Biology and Aquarium (NMMBA) in Southern Taiwan	Anti-inflammatory activity by exerted inhibition effects on inducible nitric oxide synthase (iNOS) release from LPS-stimulated RAW 264.7 cells to 79.5%, when compared with results of the cells stimulated with only LPS at concentration of 10 μM	[89]
73	Briaviodiol A (**348**)	Known	*Briareum violaceum*	Cultivation tank at the National Museum of Marine Biology and Aquarium (NMMBA) in Southern Taiwan	Anti-inflammatory activity by exerted inhibition effects on inducible nitric oxide synthase (iNOS) release from LPS-stimulated RAW 264.7 cells to 61.9%, when compared with results of the cells stimulated with only LPS at concentration of 10 μM	[89]
74	Xishaflavalin G (**349**)	New	*Lemnalia flava*	Xisha Islands, South China Sea, China	No inhibitory effects on the ConA-induced T lymphocytes and/or lipopolysaccharide-(LPS)-induced B lymphocytes proliferation	[90]
75	Xishaflavalin H (**350**)	New	*Lemnalia flava*	Xisha Islands, South China Sea, China	No inhibitory effects on the ConA-induced T lymphocytes and/or lipopolysaccharide-(LPS)-induced B lymphocytes proliferation	[90]
76	Nephthenol (**351**)	Known	*Lemnalia flava*	Xisha Islands, South China Sea, China	Inhibit the proliferation of ConA-induced T lymphocyte cells and/or LPS-induced B lymphocyte cells in vitro, with IC_50_ values of 10.7 and 38.6 μM, respectively	[90]
77	4-Hydroxy-1-(16-methoxyprop-16-en-15-yl)-8-methyl-21,22-dioxatricyclo [11.3.1.1^5,8^] octadecane-3,19-dione (**352**)	New	*Stomopneustes variolaris*	South-east coast of Arabian Sea (Kadiapattanam coast)	Anti-inflammatory activity through inhibiting 5-lipoxygenase with IC_50_ of 2.01 mM, compared to ibuprofen (IC_50_ 4.50 mM) with selectivity ratio of COX-1 to COX-2 for the studied compound was found to be greater (1.25) than that of ibuprofen (0.43). Potent anti-oxidant activity through DPPH and ABTS^+^ scavenging activity with IC_50_ values of 1.41 and 1.61 mM, respectively, and found to be greater than the standard agent α-tocopherol (IC_50_ 1.51 and 1.70 mM, respectively)	[91]
78	Briaviolide Y (**353**)	Known	*Briareum excavatum*	Off the coast of Lanyu Island, Taiwan	Anti-inflammatory activity through significantly reducing the release of COX-2 to 65.30% at 10 µM in RAW 264.7 macrophages stimulated by LPS	[92]
79	Briaviolide Z (**354**)	Known	*Briareum excavatum*	Off the coast of Lanyu Island, Taiwan	Anti-inflammatory activity through significantly reducing the release of iNOS to 60.29% at 10 µM in RAW 264.7 macrophages stimulated by LPS	[92]
80	Briarenol L (**355**)	New	*Briareum excavatum*	Off the coast of Lanyu Island, Taiwan	No inhibition against iNOS and COX-2 expression at 10 µM from RAW 264.7 macrophages stimulated by LPS	[92]
81	Briarenol W (**356**)	New	*Briareum stechei*	Cultured in the National Museum of Marine Biology and Aquarium (NMMBA), Pingtung, Taiwan	Inactive in anti-inflammatory activity assay by assessing the release of iNOS and COX-2 in LPS-stimulated RAW 264.7 macrophage cells	[93]
82	Briarenol X (**357**)	New	*Briareum stechei*	Cultured in the National Museum of Marine Biology and Aquarium (NMMBA), Pingtung, Taiwan	Showed anti-inflammatory activity by enhancing the release of iNOS and COX-2 (142.03 and 159.21%, respectively) in LPS-stimulated RAW 264.7 macrophage cells at concentration of 10 µM	[93]
83	Briarenol Y (**358**)	New	*Briareum stechei*	Cultured in the National Museum of Marine Biology and Aquarium (NMMBA), Pingtung, Taiwan	Inactive in anti-inflammatory activity assay by assessing the release of iNOS and COX-2 in LPS-stimulated RAW 264.7 macrophage cells	[93]
84	Briarenol Z (**359**)	New	*Briareum stechei*	Cultured in the National Museum of Marine Biology and Aquarium (NMMBA), Pingtung, Taiwan	Inactive in anti-inflammatory activity assay by assessing the release of iNOS and COX-2 in LPS-stimulated RAW 264.7 macrophage cells	[93]
85	Solenolide A (**360**)	Known	*Briareum stechei*	Cultured in the National Museum of Marine Biology and Aquarium (NMMBA), Pingtung, Taiwan	Showed anti-inflammatory activity by enhancing the release of iNOS and COX-2 (134.11 and 196.03%, respectively) in LPS-stimulated RAW 264.7 macrophage cells at concentration of 10 µM	[93]

*Sinularia* sp. from Xisha Islands yielded four new cembranoids named 1*E*,3*E*,7*E*,-11-hydroxy-12-methoxy-1-isopropyl-4,8,12-trimethyl-icyclotetradeca-1,3,7-triene **200**¸ 3*E*,7*E*-11-hydroxy-12-methoxy-1-isopropenyl-4,8,12-trimethyl-icyclotetradeca-3,7-diene **201**, 1*E*,3*Z*,7*E*,-11-hydroxy-12-methoxy-1-isopropyl-4,8,12-trimethyl-icyclotetradeca-1,3,7-triene **202**, and 1*Z*,3*Z*,7*E*,-11-hydroxy-12-methoxy-1-isopropyl-4,8,12-trimethyl-icyclotetradeca-1,3,7-triene **203**. The study showed that **201** and **203** had no biological activity [60]. *Sinularia scabra* from Hainan, China, contained ten cembranoids. Six of them were novel compounds, namely, xiguscabrates A-B **204**–**205**, xiguscabral A **206**, xiguscabrols A-B **207–208**, and 8-*epi*-xiguscabrol B **209**, with **204** and **206** not yet found to have biologically activity as tested. The known compound were sinulariol C **177**, sinulariolide **190**, (2*R*,11*S*,12*S*)-isosarcophytoxide **210**, and ( −)-14-deoxycrassin **211** [52]**. **Figure 8 shows the structure of cembranoids isolated from *Sinularia* sp.

**Fig. 9:**
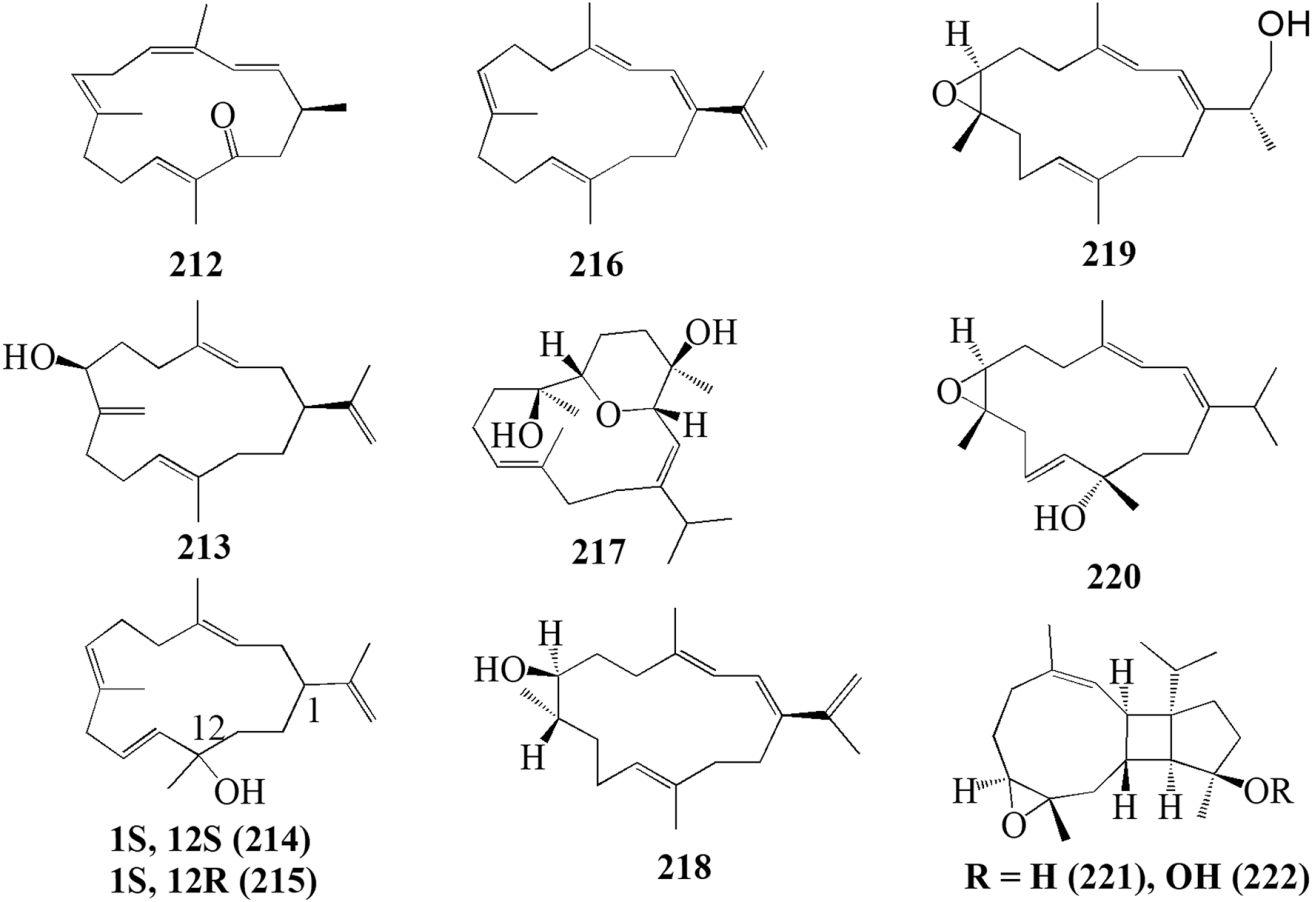
Cembranoids isolated from *Sinularia crassa* (**212–216**) and *Sinularia humilis* (**217–222**)

*Sinularia crassa* from West Island, South China Sea contained four new and one known cembrane-type diterpenoids; sinulacrassins A-C **212–214**, *ent*-xishaflavalin G **215**, and S-(+)-cembrane A **216** (Fig. 9). Compound 212 was not tested for its activity, while compound 213 and 216 showed a potential inhibitory effect towards α-Glucosidase [61]. Lastly, six novel compounds were reported from *Sinularia humilis* collected from Ximao Islands, Hainan, China namely humilisins A-F **217–222**. Compound 222 was the only reported diterpenoid that possessed biological activity by decreasing NO level in anti-inflammatory assay [62].

### Cembranoids Reported from Genus Lobophytum

**Fig. 10:**
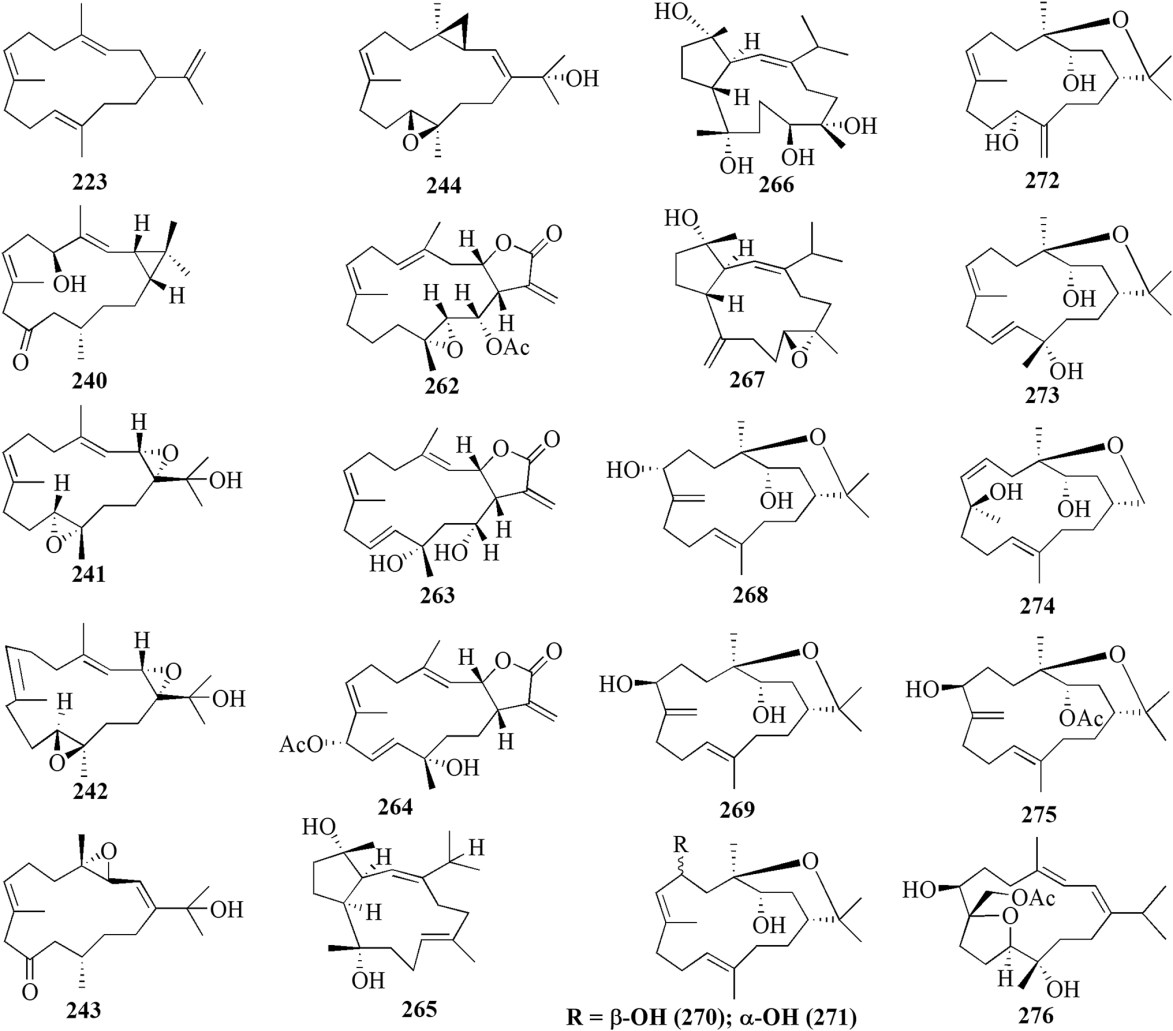
Cembranoids reported from *Lobophytum* sp

**Fig. 11:**
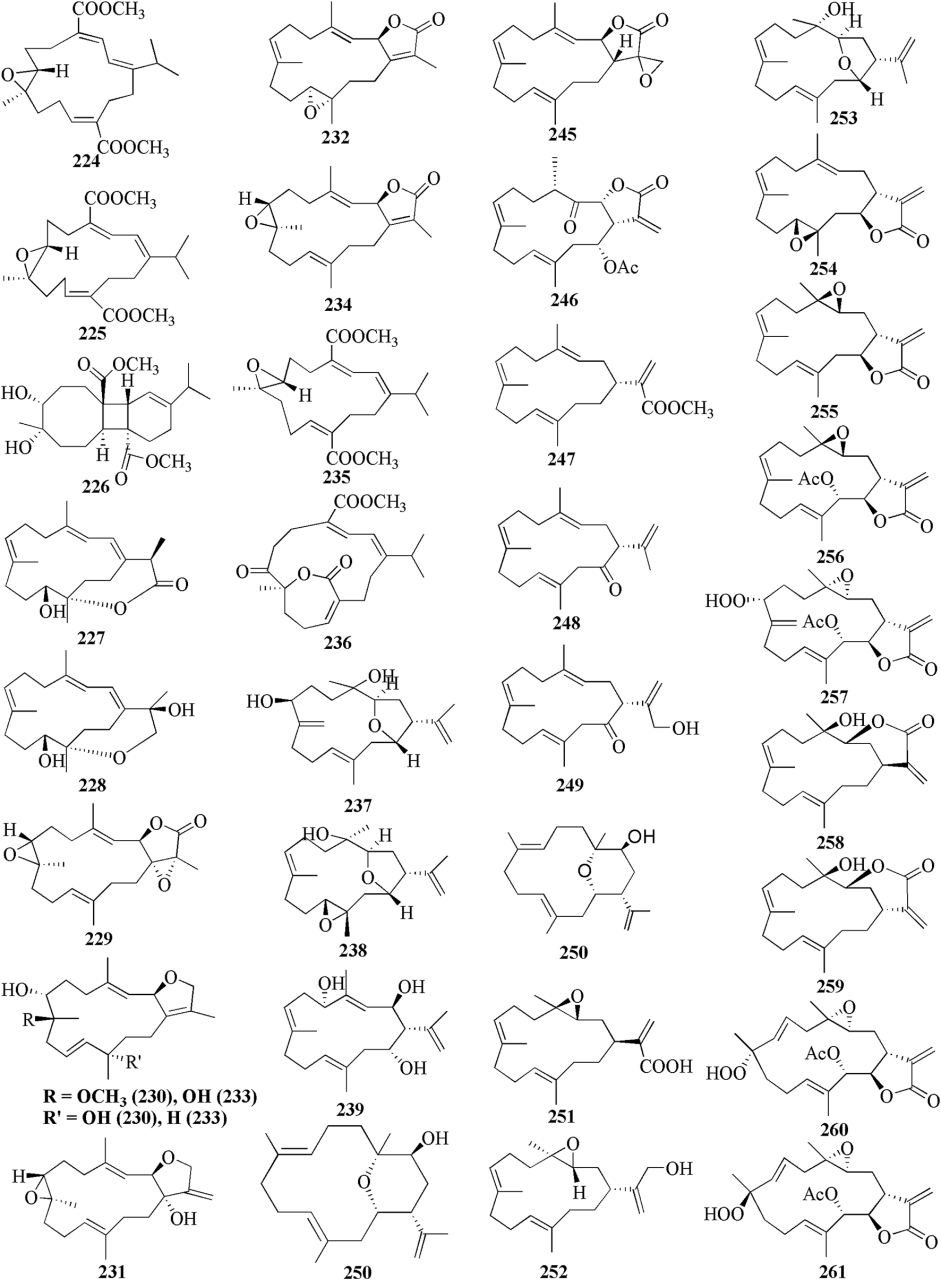
Cembranoids reported from *Lobophytum crassum*

The present study reported 47 cembranoid compounds isolated from *Lobophytum* sp. collected from various geographical areas (Figs. 10, 11). Twenty-nine of those were new compounds and the other 18 were previously known compounds with newly discovered activities. Twelve of the new compounds were newly discovered and have not been thoroughly tested for their biological activities.

*Lobophytum crassum* was found to produce different cembranoid compounds. Cembrene A **223**, a new cembranoid diterpene, was isolated from Red Sea *Lobophytum* sp. in Jeddah [63]. Ten new cembranoids and three known cembranoids were isolated from Hainan *Lobophytum crassum* in Meishan, China. Locrassumin A **224** and G **230** were the new compounds showing biological activities, whereas locrassumins B-F **225–229**, (−)-laevigatol B **231**, (−)-isosarcophine **232**, and (−)-*7R,8S*-dihydroxydeepoxy sarcophytoxide **233** were the new compounds that have not been tested yet for their biological activity. Meanwhile, three known compounds with new activities were *ent*-sarcophine **234**, sarcophytonolide O **235**, and ketoemblide **236** [64]. Three new-non tested compounds, lobophylins F–H **237–239**, were isolated from Dongsha Atoll L. *crassum* [65].

Another study discovered a Japanese *Lobophytum* sp. that produced one new casbane-type diterpenoid and two new cembrane diterpenoids (compound 1–3 **240–242**) with various biological activities. Moreover, it also produced two known compounds, grandilobatin B **243** and sinugibberol **244** [66]. The latter study reported that five cembranoids was obtained from from *Lobophytum crassum* collected from the coast of Pingtung, Taiwan. Two of them were new compounds named lobophyolides A-B **245–246**, whereas three were known compounds called 16-methoxycarbonyl cembrene A **247**, sinarone **248**, and sinaluriol D **249** [11]. In the same sampling area, twelve compounds were reported from the aquaculture *Lobophytum crassum*. Two compounds were new (culobophylin D **250,** and culobophylin E **251**) while the others were known compounds including lobocrassin C **252**, lobophylin **253**, crassocolide E **254**, sarcocrassocolide **255**, 13-acetoxysarcocrassocolide **256**, sarocrassocolide M **257**, (*R*)-14-deoxycrassin **258**, lobocrassin B **259**, sarcocrassocolides F-G **260–261** [67]. Recently, a known compound 13-acetoxysarcocrassocolide **256** was also reported from the same aquacultured *Lobophytum crassum* by Liu et al. [68].

Three new unnamed cembranolide diterpenes (compound 4–6 **262–264**) with various biological activities were isolated from Irabu Island *Lobophytum* sp. which have [69]. Furthermore, three new capnosane-type diterpenoids with no biological activities named lobophytrols A-C **265–267** were isolated from *Lobophytum* sp collected in Weizhou Island, China [70]. Lastly, new macrocyclic cembranoids lobophytolins A-B **268–269** isolated from *Lobophytum* sp. were collected from Xisha Islands, China, with both compounds not showing any biological activities [71]. Lastly, seven unreported cembranoid was isolated from *Lobophytum* sp. collected from the Xisha Island, Hainan, China. The new cembrane-type diterpenes, namely, lobophytolins C-I **270–276,** displayed various anti-cancer activity towards HT-29, Capan-1, A549, and SNU-398 cancer cell line. Moreover, they also exhibited a weak inhibitory effect of XBP-Splicing on B16-F10 tumor cells [72].

### Cembranoids from Other Soft Corals Species

The present study reported 80 cembranoid compounds isolated from other than the above-mentioned soft coral species collected from various geographical areas (Fig. 10). Fifty-five were new compounds and the other 25 were previously known compounds with newly discovered activities. Ten of the new compounds were newly discovered and have not been thoroughly tested for their biological activities.

In 2016, a known cembranoid named claudieunicellin S **277** was isolated from *Cladiella tuberculosa* collected from Penghu Archipelago waters, Taiwan [73]. Six new briarane-type diterpenoids were isolated from Taiwanese *Briareum* sp. named briarenolides ZI-ZVI **278–283**. Among these, **279** and **283** showed biological activities [74]. Later in 2016, three new cembranoids were isolated from *Nephthea* sp. collected from Sabah, Malaysia. 10-hydroxy-nephthenol acetate **284** and 7,8-epoxy-10-hydroxy-nephthenol acetate **285** were found to be biologically active, whilst 6-acetoxy-7,8-epoxy-10-hydroxy-nephthenol acetate **286** was not tested yet for its biological activity [75]. *Junceella fragilis* from Hainan Island, China contained five new briarane diterpenoids named 3-deacetylpraelolide **287**, 13-α-acetoxyl-3-deacetylpraelolide **288**, 13-α-acetoxyl-2-deacetylpraelolide **289**, 13-α-acetoxyl-3-deacetyljunceellin **290**, and 13-α-acetoxyl-2-deacetyljunceellin **291** [76]. Several cembranoids were also isolated from *Klyxum flaccidum* originated from Hsiao Liuchiu Island, Taiwan, named klyflaccicembranols A-I **292–300** and gibberosene D **301**. Klyflaccicembranol G **298** was the only compound that has not been tested for its biological activities [77].

A novel cembrane has been isolated from *Chicoreus ramosus* collected in fishing harbors between Sri Lanka and India, namely (3E, 6E, 10E)-8a-butoxy-17(15 → 14), 20(12 → 11)-bis-abeo-cembra-3,6,10,14(17),15-pentaene **302** [78]**.** Meanwhile, nine new compounds were isolated from *Eunica* sp. collected from Caribbean Sea, namely compound 7–15 **303–311**. Among these compounds, **304** was the only one showing biological activities, whilst the other compounds were not tested yet. Moreover, five known cembranoids were also isolated this species, namely euniolide **312**, 14-deoxycrassin **313**, pseudoplexauric acid methyl ester **314**, (1S*,3S*,4S*,7*E*,11*E*)-3,4-epoxy-13-oxo-7,11,15-cembratriene **315**, and (–)-eunicenone **316** [79].

The Bornean soft coral *Nephtea* sp. collected from Mantanani Island, Sabah, was found to produce new cembranoid norditerpene, i.e. chabrolene **317** [80]. *Pseudoplexaura flagellosa* collected from Colombia was reported to have two known cembrane diterpenes, namely asperdiol acetate **318** and knightal **319** [81]. Known compounds 14-acetoxycrassine **320** and asperdiol **321** were successfully obtained from *Pseudoplexaura porosa* and *Eunicea knighti* collected in the Caribbean sea, respectively[82]. In 2019, Tseng and co-workers reported cembranoids from *Klyxum flaccidum* collected from Pratas Island, Taiwan. This species contained two new compounds, which are flaccidodioxide **322** and flaccidodiol **323**. The later compound was reported to possess no activities. Moreover, a known compound 14-O-acetylsarcophytol B **324** was also reported from the same species [83]. *Junceella fragilis* collected from Vietnam was reported to produce new briarane-type diterpenoids, 17-*epi*-junceellolide B **325** and junceellolide B **326**. While **325** did not possess any activities, the later showed new activities than before [84].

Aquacultured *Briareum violaceum* has been reported to contain four novel hydroperoxyfurancembranoids, namely briaviodiols B-E **327–330**. One compound named briaviodiol C **328** did not possess any activity [85]. In 2019, three new-non active triacetoxybriaranes were isolated from *Junceella fragilis* in Lanyu Island, Taiwan, namely fragilides M–O **331–333** [86]. Eight chlorinated briarane diterpenoids were isolated from *Erythropodium caribaeorum* originated from Providencia Island, Caribbean. Among these, six known compounds, namely erythrolides A-B **334–335**, erythrolide D **336**, erythrolide F **337**, erythrolide J **338**, and erythrolide U**339,** were reported to have new activities. However, the new compounds erythrolides W-X **340–341** showed no biological activities [87]. *Cladiella* sp. from Taiwan contained two new eunicellin diterpenoids cladieunicellins U-V **342–343** and two known eunicellin diterpenoids sclerophytins A-B **344–345** [88].

Aquacultured *Briareum violaceum* from Southern Taiwan was found to yield three new furanocembranoids and one known furanocembranoid. The new-non active compound was briaviodiol F **346**, while the two other new compounds named briaviotriols A-B **347–348** were biologically active. Briaviodiol A **349** was the only compound that had been isolated before [89]. Xishaflavalins G-H **350–351** were the new isolated cembrane from Chinese soft coral *Lemnalia flava* which did not show any activities, whereas new activities were reported from the known cembrane nephthenol **352** [90]. *Stomopneustes variolaris* from the Arabian Sea contained new cembrane named 4-hydroxy-1-(16-methoxyprop-16-en-15-yl)-8-methyl-21,22-dioxatricyclo [11.3.1.1^5,8^] octadecane-3,19-dione **353** [91]. Lastly, B. *excavatum* from Lanyu Island, Taiwan, contained two known briarane diterpenoids named briaviolides Y–Z **354–355** and one new-non active briarane diterpenoid named briarenol L **356** [92].

## Biological Activities

Cembranoids and their analogues have been reported to have various biological activities such as anti-cancer, anti-bacterial, anti-inflammation, anti-diabetic, neurological activity, anti-fouling, toxicity to brine shrimp, immunosuppressant, anti-Alzheimer’s, anti-oxidant, repellent activity against *Sitophilus zeamais*, and acetylcholinesterase (AChE) inhibition activity. The reported total numbers of cembranoid compounds from genera *Sarcophyton*, *Sinularia*, *Lobophytum*, and other species that were successfully identified were 139, 42, 47, and 80, respectively. Among them, 221 were newly isolated compounds, and the other 87 compounds were previously known with newly discovered activities. The remaining 34 new compounds have not been tested for their biological activities.

### Anti-bacterial

Compound **1** showed antibacterial activity against *Staphylococcus aureus,* with minimum bactericidal concentration (MBC) and minimum inhibitory concentration (MIC) values of 75 and 25 μM, respectively [17]. Compound **2** isolated from *Sarcophyton trocheliophorum* also possessed moderate antibacterial activity against *Bacillus subtilis, Staphylococcus aureus,* and *Vibrio cholerae* with MIC values of 125, 100 and 125 mg/mL, respectively, but it did not have activity against *Escherichia coli* [18]*.* Compound **8**, exhibited antibacterial activity against several bacteria, viz. *Acinetobacter baumannii* (MIC = 4.2 μM), *Escherichia coli* (MIC = 6.0 μM), *Klebsiella pneumoniae* (MIC = 5.8 μM), *Pseudomonas aeruginosa* (MIC = 5.2 μM), *Staphylococcus aureus* (MIC = 4.0 μM), *Staphylococcus epidermidis* (MIC = 5.7 μM), and *Streptococcus pneumoniae* (MIC = 6.0 μM). While, **9** and **10**, which were also tested against the bacteria mentioned above, showed weak antibacterial activity. Compound **9** was reported to have inhibition zones of 7, 8, 7, and 7 mm zones of 11, 11, and 6 mm against *Klebsiella pneumonia, Staphylococcus aureus, and Staphylococcus epidermidis*, respectively [21]. The compound from *Staphylococcus trocheliophorum,* **84**, exerted moderate antibacterial activity against *Staphylococcus aureus* with MIC value of 250 μM [36]. Additionally, **85** exhibited anti-fungal activity towards *Ochroconis humicola* and *Haliphthoros milfordensis* with MIC value of 6.25 μg/mL [37].

Compound **223** isolated from *Lobophytum* sp. showed moderate anti-bacterial activity against *Acinetobacter* sp., *Escherichia coli*, *Klebsiella pneumonia*, *Pseudomona aeruginosa*, *Staphylococcus aureus*, *Staphylococcus epidermidis*, and *Streptococcus pneumonia*. It had inhibition zone diameters of 14, 13, 13, 13, 11, 11, 11 mm, respectively and MIC value of 30 μg/mL against those bacteria [63]. The Okinawan *Lobophytum* sp. produced five cembranoid compounds (**240–244**) that exhibited antibacterial activity against *Staphylococcus aureus* and *Eschericia coli.* At a concentration of 25 μg compound 199–203 had an inhibition zone of 10, 9, 9, 10, 10 mm, respectively against *Staphylococcus aureus* and 10, 10, 10, 12, 15 mm, respectively against *Escherichia coli* [66]. Furthermore, cembranoids isolated from *Nephthea* sp., **284** and **285,** exerted anti-bacterial activity against *Staphylococcus aureus* with MBC of 180 and 150 μg/mL, respectively and *Eschericia coli* with MBC of 75 and 75 μg/mL, respectively [75].

### Anti-cancer

New compounds **24**, **25** and **26** isolated from *Sarcophyton ehrenbergi* showed low to moderate anti-proliferation activity against A549 human lung carcinoma cells with inhibition concentration 50 (IC_50_) values of 50.1, 76.4, and 50.8 μM, respectively, but inactive towards Caco-2 human colorectal adenocarcinoma cells. Compounds **24** and **26** also exhibited low to moderate cytotoxicity to HepG2 human liver carcinoma cells with IC_50_ values of 98.6 and 53.8 μM [17]. In addition, the known compounds **27–31** isolated from *Sarcophyton glaucom* also exerted moderate to potent activity against HepG2. Compound **27** and **28** were tested together and exerted effective concentration 50 (EC_50_) value of 11.32 µg/mL, while compound **29**–**31** possessed EC_50_ values of 17.84; 9.97; and 10.32 µg/mL, respectively [26]. Another study reported anti-cancer activity against MCF-7 human breast cancer cells from compounds **30** and **31** with IC_50_ values of 24.97 and 22.39 μg/mL, respectively [32].

Compounds **42**–**47** also showed cytotoxic activity towards MCF-7, with growth inhibition 50 (GI_50_) values of 18.13; 12.22; 24.2; 22.27; 18.88; and 20.041 ppm, respectively [29]. Compound **62** extracted from *Sarcophyton mililatensis* was reported to have strong cytotoxicity towards HL-60 human leukemia cells and A549 cells, with IC_50_ values of 0.78 μmol/mL and 1.26 μmol/mL, respectively [31]. New compounds **92–96** showed cytotoxicity towards MCF-7 cells with IC_50_ values of 23.84; 26.22; 26.81; 25.28; and 27.2 μg/mL, respectively [32]. New potent anti-cancer activity from known compounds **103**, **104** and **105** isolated from *Sarcophyton ehrenbergi* was reported against A549 cells, with inhibition concentration 25 (IC_25_) values of 23.3, 27.3, and 25.4 μM, respectively. However, they were not active against Caco-2 cells. Additionally, **104** and **105** exhibited weaker activity against HepG2 cells with IC_25_ values of 22.6 and 31.8 μM, respectively [40]. Finally, from genus *Sarcophyton*, **139**, a known compound isolated from *Sarcophyton glaucum* exhibited anti-proliferation activity against HEK293 human embryonic kidney cells with lethal dose 50 (LD_50_) of 123.5 mM [44].

Compound 141, 145–147 isolated from *Sarcophyton digitatum* showed anti-cancer activity towards various cancer cell line. Compound 141 showed cytotoxicity against MCF-7 and MDA-MB-231 with IC_50_ of 9.6 ± 3.0 and 14.8 ± 4.0 µg/mL, respectively. Moreover, 145 showed cytotoxicity towards MCF-7, HepG2, and HeLa with IC_50_ values of 10.1 ± 3.3; 14.9 ± 3.5; and 17.1 ± 4.5 µg/mL, respectively. In addition, 146 exhibited cytotoxicity towards MCF-7, MDA-MB-231, and HepG2 with IC_50_ value of 9.4 ± 3.0; 17.8 ± 4.5; 14.9 ± 4.2 µg/mL, respectively. Lastly, 147 showed cytotoxicity towards MCF-7 with an IC_50_ value of 10.9 ± 4.3 µg/mL [45].

Another study reported that cembranoid isolated from *Sarcophyton tenuispiculatum* also possessed anti-cancer activity including compound 148, 151–155. Compound 148, 151–155 showed cytotoxicity against MCF-7 with IC_50_ value of 34.3 ± 3.7; 37.6 ± 4.2; 33.3 ± 3.5; 30.1 ± 3.1; 24.3 ± 3.0; 27.2 ± 4.0 µm, respectively. Whilst compound 151–152, 154–155 showed cytotoxicity against HepG2 with IC_50_ value of 35.2 ± 4.4; 28.6 ± 3.4; 34.5 ± 4.2 and 36.4 ± 5.3 µm, respectively. Furthermore, compound 153 showed cytotoxicity towards MDA-MB-231 cell line with an IC_50_ value of 38.6 ± 5.0 µm [46].

Several compounds from the genus Sinularia were also reported to have anti-cancer activity. Compound **172** from *Sinularia erecta* showed anti-proliferation activity against K562 human leukimia cell line with an IC_50_ value of 9.2 μM [49]. Compound **178** from *Sinularia compacta* showed anti-proliferation activity against HCT-116 human colorectal carcinoma cell and A549, with IC_50_ values of 10.1 and 14.7 μM, respectively [53]. The cembranoid compound, **178**, isolated from *Sinularia* sp. found in Yongxing Island, South China Sea had anti-cancer activity was towards HeLa human cervical cancer and HCT-116 with IC_50_ values of 11.6 and 33.3 μM, respectively [54].

In 2018, Tsai et al. isolated **181** from aquacultured *Sinularia sandensis.* The compound exerted a concentration-dependent anti-proliferative effect on NCI-N87 human gastric carcinoma cells and promoted apoptosis induction. The anti-proliferation activity was associated with the release of cytochrome c from mitochondria, activation of pro-apoptotic proteins, e.g. cysteine-aspartic proteases(caspase)-3/-9, Bcl-2-associated X protein (Bax) and Bcl-2-associated agonist of cell death (Bad), and inhibition of the anti-apoptotic proteins B-cell lymphoma 2 (Bcl-2), B-cell lymphoma-extra large (Bcl-xL), and myeloid cell leukemia 1 (Mcl-1). This compound also triggered endoplasmic reticulum (ER) stress, leading to activation of the PERK/elF2α/ATF4/CHOP apoptotic pathway. Further, **181** also initiated autophagy in NCI-N87 cells and induced the expression of autophagy-related proteins, including Autophagy related (Atg)3, Atg5, Atg7, Atg12, microtubule-associated protein light chain (LC)3-I, and LC3-II [55].

Compounds **182** and **183** isolated from *Sinularia* sp. found in Sabah, Malaysia possessed anti-proliferation activity against HL-60 cancer cell line through apoptosis mechanism that involved the up-regulation of Bax, the down-regulation of Bcl-xL, and the activation of caspase-3 [56]. Wu et al. isolated 7 cembranoids, **184–190,** from *Sinularia flexibilis* whereas four of them (**187–190**) exhibited anti-proliferation activity. Compound **187** showed anti-proliferation activity against P388 mouse leukimia cells, K562, HT-29 human colon cancer cell lines, with IC_50_ values of 9.3, 23.4, and 15.9 μM, respectively. Compound **188** exhibited anti-proliferation activity against P388, K562, HT-29 cancer cell lines, with IC_50_ values of 6.9, 12.2, and 9.6 μM, respectively. Compound **189** showed anti-proliferation activity against P388 and K562 cancer cell lines, with IC_50_ values of 16.0 and 26.7 μM, respectively. Compound **190** exerted anti-proliferation activity against K562 and HT-29 cancer cell lines, with IC_50_ values of 21.7 and 27.1 μM, respectively [57]. Cembranoid **187** isolated from *Sinularia flexibilis* collected in Hainan exerted broad anti-proliferation activity against A549, HT-29, SNU-398 human hepatocellular carcinoma, and Capan-1 human pancreatic ductal adenocarcinoma cell line, with IC_50_ values of 27.4, 22.7, 8.9, and 9.4 µM, respectively [58]. **188** and **190** isolated from the same species in Hainan, China, showed moderate anti-proliferation activities against HT-29, SNU-398, and Capan-1, with IC_50_ values of 32.6; 24.9; 28.7 µM and 33.6; 24.7; 26.1 µM, respectively [58]. Compound **191** isolated from aquaculture *Sinularia flexibilis* in Taiwan exerted anti-oral cancer activity by inducing oxidative stress-mediated cell death pathways through suppressing colony formation, inducing apoptosis and cell cycle arrest, as well as inducing reactive oxygen species (ROS) as observed in three in vitro cultured human oral squamous cell carcinoma (OSCC) models (Ca9.22, SCC9 and HSC-3 cell lines) [59].

Compound **223** from *Lobophytum* sp. exerted significant anti-tumor activity against Ehrlich ascites carcinoma cells with LD_50_ of 50 μg/mL [63]. Roy et al. isolated **240**–**242** from the Okinawan soft coral *Lobophytum* sp. These compounds showed mild cytotoxicity against HCT-116, with IC_50_ values of 135.37, 177.11, and 153.11 μM, respectively [66]. Out of the twelve new cembranoids isolated from aquacultured *Lobophytum crassum* collected from the coast of Pingtung, Taiwan, **250–261**, ten showed anti-proliferation activitiy [67]. Compound 211 had IC_50_ of 35.8 μM against SUP-T1 human T-cell lymphoblastic lymphoma cell, compound 212 had activity against K562, MOLT-4 human acute T lymphoblastic leukaemia A, SUP-T1, with IC_50_ values of 16.3, 12.3, and 4.6 μM, respectively, while compounds **254–261** was active against K562, Molt 4, U937 human myeloid leukaemia cell line, and SUP-T1. The IC_50_ of compounds **254**–**261** against K562 were 11.3, 18.1, 3.3, 15.3, 4.5, 3.3, 12.3, and 13.0 μM, respectively; against MOLT-4 were 6.2, 8.4, 1.2, 11.6, 2.9, 2.3, 4.8, and 7.0 μM, respectively; against U937 were 15.8, 4.4, 7.1, 32.0, 7.0, 5.2, 10.9, and 23.3 μM, respectively; and against SUP-T1 were 5.2, 8.3, 1.5, 10.2, 4.5, 6.2, 6.1, and 6.6 μM, respectively [67].

Compound **256** from aquacultured *Lobophytum crassum* showed cytotoxic activity against Ca9-22 human oral cancer cells through ROS generation and the suppression of the anti-oxidant enzyme activity. The apoptotic effect was found to be mediated through the interruption of the Keap1/Nrf2/p62/SQSTM1 pathway. It increased the expression of apoptosis and DNA damage-related proteins in a concentration and time-dependent manner. It also exerted potent anti-tumor effect against oral cancer cells, as demonstrated by the in vivo xenograft animal model. This compound reduced the tumor volume by 55.29% and tumor weight by 90.33% [68]. In 2019, Roy et al., isolated three cembranoids, **262**–**264**, from Okinawa, Japan, which showed anti-proliferation activity against various cancer cell lines. Compound **262** showed moderate anti-proliferation activity against HeLa, A459, B16-F10 mouse skin melanoma, and RAW 264.7 mouse macrophage cells, with IC_50_ of 7.81, 9.30, 10.83, and 5.99 μM, respectively. Compound **263** exerted low anti-proliferation activity against HeLa, A459, and RAW 264.7 cells, with IC_50_ of 49.33, 54.09, and 43.74 μM, respectively. Compound **264** possessed low anti-proliferation activity against RAW 264.7 cells, with IC_50_ of 45.22 μM [69].

*Lobophytum* sp. collected from Xisha Island contained two compounds that exhibited anti-cancer activity. Compound 270 showed moderate cytotoxicity against SNU-398 with an IC_50_ value of 42.54 ± 6.26 μM. Besides, 271 exhibited anti-cancer activity towards various cancer cell line including HT-29, Capan-1, A549, and SNU-398 with IC_50_ values of 4.52 ± 0.82; 6.62 ± 4.02; 5.17 ± 0.86; 6.15 ± 2.28 μM, respectively [72]. Compound **277** isolated from *Cladiella tuberculosa* possessed moderate anti-proliferation activity against MOLT-4, K562, SUP-T1, with IC_50_ values of 6.04, 6.80, 6.90 μg/mL, respectively [73]. In 2016, Ishii et al., isolated **284** and **285** from the Bornean soft coral *Nephthea* sp. They possessed anti-proliferation activity against HeLa with IC_50_ values of 40 and 125 μg/mL, respectively, and against MCF-7 with IC_50_ values of 25 and 75 μg/mL, respectively [75]. In 2017, Ahmed et al., isolated **293** which showed anti-proliferation activity against A549 and K562 with IC_50_ values of 16.5 and 34.6 μM, respectively [77]. Four new compounds, namely **295, 296, 299** and **300**, isolated from *Klyxum flaccidum* exerted anti-cancer activity towards various cancer cell lines. **295** possessed anti-proliferation activity against K562 with IC_50_ of 44.9 μM. **297** showed anti-proliferation activity against A549 with IC_50_ of 21.4 μM. **298** exerted anti-proliferation activity against A549, K652, and P388 with IC_50_ values of 49.4, 47.4, and 34.6 μM, respectively. **300** displayed anti-proliferation activity against HT-29 with IC_50_ values of 41.9 μM [77]. Two known compounds, **317** and **318** were isolated from Colombian *Pseudoplexaura flagellosa*. **317** showed moderate cytotoxicity against PC3 human prostate cancer cell line and A549 with IC_50_ of 34.2 and 64.0 μg/mL, respectively. Further, **318** exerted moderate cytotoxicity against MDA-MB-231 human breast cancer cell, PC3, and L929 mouse fibroblast cell lines with IC_50_ of 52.7, 54.28 and 68.7 μg/mL, respectively [81]. Tseng et al. (2019) isolated **321** and **323** from Taiwanese *Klyxum flaccidum* which showed anti-cancer activity. **321** displayed low anti-proliferation activity against the P388D1 mouse lymphocytic leukemia cell line with IC_50_ of 19.6 μg/mL, while **323** showed a broad range of anti-cancer activities against A549, DLD-1 human colorectal adenocarcinoma, and P388D1 cell lines with IC_50_ values of 10.8, 11.7 and 8.9 μg/ml, respectively [83]. The known compound, **325**, isolated from Vietnamese *Junceella fragilis* showed weak cytotoxicity against the LNCaP human prostate adenocarcinoma cells with IC_50_ of 85.34 μM, as compared with that of the positive control ellipticine (IC_50_ of 1.42 μM) [84].

In 2019, Molina et al. isolated six novel cembranoids (**333–338**) which possessed anti-cancer activity towards various cancer cell lines. **333**, **334**, **335** and **337** showed cytotoxicity against A549, MCF-7 and PC3 cancer cell lines. **333** possessed anti-tumor activity against A549, MCF-7 and PC3 with IC_50_ values of 18.41, 6.77 and 2.45 μM, respectively. **334** exerted anti-proliferation activity against A549, MCF7, and PC3 cancer cell lines with IC_50_ values of 27.09, 15.21, and 6.46 μM, respectively. **335** possessed anti-proliferation activity against A549, MCF7, and PC3 cancer cell lines with IC_50_ values of 2.58, 42.45, and 60.00 μM, respectively. **337** exerted anti-proliferation activity against A549, MCF7, and PC3 cancer cell lines with IC_50_ values of 37.93, 56.06, and 42.49 μM, respectively. **337** and **338** showed low anti-proliferation activity against the A549 cancer cell line with IC_50_ of 46.49 and 36.65 μM, respectively [87]. *Cladiella* sp. from Penghu Archipelago contained three new cembranoids (**341, 342** and **344**) which possessed anti-cancer activity. **341** and **344** exhibited moderate anti-proliferation activity toward the leukemia K562 cells with IC_50_ of 12.76 and 11.39 μg/mL, respectively while **342** showed moderate anti-proliferation activity toward the leukemia MOLT-4 cells with IC_50_ of 18.83 μg/mL [88].

### Anti-inflammation

Two novel compounds isolated from *Sarcophyton elegans*, **18** and **19**, showed anti-inflammatory activity by inhibition of lipopolysaccharide (LPS)-induced nitrite oxide (NO) production by RAW 264.7 macrophages with IC_50_ values of 18.2 and 32.5 μM, respectively [24]. Compound **31** isolated from *Sarcophyton glaucom* had inhibition activity towards the expression of inducible nitrite oxide synthase (iNOS) at 50 and 100 μM. This compound also showed activity against the expression of cyclooxygenase-2 (COX-2) at 25, 50, and 100 μM in RAW 264.7 [33]. Other anti-inflammatory activities were also reported from a new compound, **57,** and a known compound, **62.** These two compounds showed inhibitory activity towards Tumor Necrosis Factor α (TNF-α)-induced nuclear factor kappa B (NF-κB) activation (a therapeutical target in cancer), with IC_50_ values of 35.23 and 22.52 μmol/mL, respectively [31].

Novel compounds **69–73**, and known compound **75**, isolated from *Sarcophyton cherbonnieri* exhibited anti-inflammatory activity by the inhibition of N-formylmethionine-leucyl-phenylalanine/cytochalasin B (fMLF/CB)-induced superoxide anion generation and estalase release in human neutrophils at various potentials. Moderate inhibition activities were shown by **69**, **71** and **74** with respective values of 32.1, 44.5, and 64.6% superoxide anion generation, and 37.6, 35.6, and 42.6% elastase release at 30 μM were reported. Weaker activities were exerted by **70, 72**, **73** and **75** with inhibitory effects of 4.0, 6.4, 2.6, and 3.5% on superoxide anion generation, and inhibition by 23.5, 27.6, 30.5, and 20.7% on elastase release have been reported [34]. Three known compounds **97, 98**, and **102**, as well as the newly discovered compound, **100**, isolated from *Sarcophyton ehrenbergi*, exerted anti-inflammatory activity by TNF-α secretion inhibition in RAW 264.7. The most potent activity was exhibited by **98** with IC_50_ similar to dexamethasone as the positive control (8.5 μM vs. 8.7 μM, respectively). Meanwhile, the other three had moderate effects, with IC_50_ values of 28.5, 24.2, and 27.3 μM [39]. Other studies also reported several new and known compounds with similar activity. The IC_50_ of the three new compounds **129**, **130,** and **133** were 21.3, 30.8, and 38.6 μM, respectively, while those of the five known compounds **134–138** were 9.1, 15.4, 29.5, 12.5, and 7.2 μM, respectively [43].

Compounds isolated from the soft coral *Sinularia erecta*, **170** and **171**, exhibited anti-inflammatory activity through the inhibition of superoxide generation and elastase release in fMLP/CB-induced human neutrophils, with IC_50_ values of 2.3 and 8.5 μM, respectively [49]. Taiwanese *Sinularia nanolobata* contained four new cembranoids, **174–177**. Only **177** showed anti-inflammatory activity in RAW 264.7 cells induced by LPS and it effectively reduced the levels of NO to 2.3% at a concentration of 100 µM. Moreover, **177** at a concentration of 50 µM also exhibited good inhibitory activity against iNOS compared to the positive control aminoguanidine (AG). The level of NO was also reduced significantly to 19.6% while giving a 104.6% retention of cell viability [51]. The Bornean soft coral *Sinularia* sp. contained **182** and **183** which showed anti-inflammatory activity through inhibition of NO, prostaglandin E_2_ (PGE_2_), Interleukin (IL)-1β, IL-6, and iNOS in LPS-induced RAW 264.7 macrophages. Compounds **182** and **183** showed the most potent activity on the inhibition of NO production at 12.5 and 25.0 µg/mL compared to that of the negative control. The inhibition against PGE_2_ in LPS-induced RAW 264.7 macrophages of **182** and **183** were shown in a dose-dependent manner. Both compounds also showed significant inhibition against the accumulation of interleukin (IL-1β and IL-6) production at 25.0 µg/mL, with a reduction of less than 10% to both interleukins. The inhibition of NO, IL-1β, and IL-6 shown by **182** and **183** through the downregulation of iNOS expression. Weak inhibition was displayed against PGE_2_ by slight suppression of COX-2 expression [56]. Compound **188** isolated from species collected in Hainan, China, showed high anti-inflammatory activity through inhibition of TNF-α, with an IC_50_ of 2.7 µM [58].

Among several compounds isolated from *Sinularia flexibilis* collected in Liuqiu, only compound **189** showed anti-inflammatory properties by significantly inhibiting the release of superoxide anion generation and elastase with IC_50_ values of 10.8 and 11.0 µM, respectively [94]. Seven of eight cembranoids successfully isolated from *S. flexibilis* (**188, 190, 195, 196, 197, 198,** and **199**) showed anti-inflammatory activity through the inhibition of TNF-α, with IC_50_ values of 2.7, 4.7, 20.7, 38.9, > 50, 13.3, and 4.2 µM, respectively [58].

Hainan soft coral *Lobophytum crassum* contained 13 cembranoids (**224–236**), five of which (**224**, **230**, **234**, **235**, **236**) showed moderate anti-inflammatory activity through inhibition against LPS-induced NO production, with IC_50_ values of 17, 13, 24, 8, and 12 μM, respectively [64]. The Okinawan soft corals *Lobophytum* sp. were found to contain the cembranoids **240**, **241** and **242** that exhibited anti-inflammatory activity through reducing NO production, with IC_50_ values of 41.21, 64.96, and 74.76 μM, respectively [66]. Lai et al. [11] isolated **245** to **249** from *Lobophytum crassum,* which showed potent anti-inflammatory activity through inhibition of LPS induced IL-12 release by dendritic cells (DC), with inhibition potency of 93.4, 93.6, 86.3, 77.0 and 86.4%, respectively. At the same time, inhibition of LPS induced NO release by DC of these five compounds (**245–249)** were recorded at values of 93.5% with DC survival at 76.0%, 95.9% with DC survival at 52.0%, 86.1% with DC survival at 75.0%, 54.9% with DC survival at 85.0%, and 86.1% with DC survival at 85.0% [11]. Cembranoids **262**, **263** and **264** from the Okinawan soft coral *Lobophytum* sp. displayed anti-inflammatory effects through the suppression of NO production in a dose-dependent manner with IC_50_ of 10.67, 13.92, and 14.02 µM, respectively after 24 h in LPS-stimulated RAW 264.7 macrophage cells, at non-cytotoxic concentrations [69].

Two new compounds isolated from *Briareum sp.* (**279** and **283**) displayed anti-inflammatory activity by reducing iNOS level to 47.2% and 55.7%, respectively, at a concentration of 10 μM [74]. From a collection of Hainan *Junceella fragilis*, five cembranoids (**287**, **288, 289**, **290, 291**) were isolated that exerted anti-inflammatory activity through the inhibition of NO production by 39.4, 42.7 (**288** and **289** were tested together) and 36.3% (**290** and **291** were tested together), respectively (at 50 μM) in RAW 264.7 cells [76]. Ten cembranoids (**292–301**) isolated in 2017 from *Klyxum flaccidum*, of which 8 (**292, 294–297, 299, 300, 301**) possessed various anti-inflammatory activities. **292, 294, 299, 301** showed weak NO inhibitory activity with 25, 12, 20, 15% inhibition, respectively, while **295** exerted moderate NO inhibition up to 65% (IC_50_ of 46.7 μg/mL) and **297** up to 64% (IC_50_ value of 47.0 μg/mL). Furthermore, **296** and **300** strongly inhibited 88% and 87% of NO production at 50 µg/ml, respectively [77].

A novel cembranoid from *Chicoreus ramosus*, **302**, showed anti-inflammatory activity through the inhibition of 5-lipooxygenase, with IC_50_ of 0.76 mg/mL [78]. Anti-inflammatory activity was evident in **323** isolated from *K. flaccidum,* predicted to occur by a reduction in the level of elastase release to 59.66%, with IC_50_ of 7.22 µM at a concentration of 10 µM relative to the control group [83]. Three out of four new cembranoids (**326, 328, 329**) isolated from cultured type *Briareum violaceum* possessed anti-inflammatory activity in LPS-induced RAW 264.7 macrophage cells by significantly inhibiting the expression of iNOS protein to 43, 61, 46%, respectively [85]. Four new compounds (**341–344**) isolated in 2019 displayed various anti-inflammatory activities. Compounds **341** and **343** decreased the release of elastase with inhibition rates of 12.01% and 11.35%, respectively, while **342** decreased the generation of superoxide anions by human neutrophils with the inhibition rate of 13.43%, and **344** had an inhibition rate of 28.12%. Additionally, **344** also decreased the release of elastase with the inhibition rate of 16.37% [88]. Three new cembranoids (**346–348**) isolated from aquacultured *B. violaceum* possessed anti-inflammatory activity by suppressing the release of inducible nitric oxide synthase (iNOS) in LPS-stimulated RAW 264.7 cells with values of 67.7, 79.5, and 61.9%, respectively, compared to the results of the cells stimulated with only LPS at a concentration of 10 μM [89]. Anti-inflammatory activity was also shown by the Arabian soft coral *Stomopneustes variolaris,* which produced the novel compound **352** that inhibited 5-lipoxygenase with IC_50_ of 2.01 mM, as compared to positive control ibuprofen (IC_50_ 4.50 mM). The selectivity ratio of cyclooxygenase-1 (COX-1) to COX-2 for the studied compound was found to be greater (1.25) than that of ibuprofen (0.43) [91]. Two known compounds isolated from *Briareum excavatum*, **353** and **354**, displayed an anti-inflammatory effect, where **353** significantly reduced the release of COX-2 to 65.30% at 10 µM in RAW 264.7 macrophages stimulated by LPS. In comparison, **354** showed anti-inflammatory activity through significantly reducing the release of iNOS to 60.29% at 10 µM using the same model [92].

Known cembranoid **145** and **147** isolated from *Sarcophyton digitatum* showed anti-inflammatory activity through inhibiting the production of IL-1β to 68 ± 1 and 56 ± 1%, respectively in LPS-stimulated murine macrophages J774A.1 at a concentration of 10 µg/mL with IC_50_ values of 10.7 ± 2.7 and 14.9 ± 5.1 µg/mL.[45]. In addition, *Sarcophyton tenuispiculatum* contained **156** which possessed anti-inflammatory activity through inhibiting the production of IL-1β to 56 ± 1% in LPS-stimulated murine macrophage J774A.1 cell at a concentration of 30 µm [46]. New briaranes **357** and **360** exhibited anti-inflammatory activity by enhancing the release of iNOS (142.03 and 134.11%, respectively) and COX-2 (159.21 and 196.03%, respectively) in LPS-stimulated RAW 264.7 macrophage cells at concentration of 10 µM [93]. *Sarcophyton roseum* collected from Egypt contained **158** which possessed anti-inflammatory activity via iNOS inhibition with IC_50_ of 50 µM. Whilst, from the same species, **161** was isolated and showed anti-inflammatory activity via Nrf-2 induction at 100 μM (2.1-fold), 50 μM (1.4-fold), and 25 μM (0.9-fold). Furthermore, **162** exhibited anti-inflammatory activity via iNOS inhibition with IC_50_ of 39 µM and Nrf-2 induction at 100 μM (1.8-fold), 50 μM (1.5-fold), and 25 μM (1.5-fold) [47].

*Sarcophyton cherbonnieri* contained cembranoids which possessed anti-inflammatory activity namely **163–169**. Compound **163–169** showed inhibition on superoxide anion generation to 11.0 ± 8.7; 29.8 ± 9.8; 44.5 ± 7.9; 6.4 ± 7.3; 6.2 ± 5.5; 12.9 ± 11.4; and 17.1 ± 11.6%, respectively, at concentration of 30 µM. Furthermore, those compounds also inhibited the release of elastase to 35.1 ± 10.6; 48.2 ± 12.5; 35.6 ± 10.7; 27.6 ± 12.8; 29.7 ± 11.1; 16.7 ± 10.2; and 27.6 ± 12.0%, respectively, at concentration of 30 µM [48]. Lastly, diterpenoid **222** isolated from *Sinularia humilis* collected in Ximao Islands have significant anti-inflammatory effects in LPS-stimulated BV-2 microglial cells with 83.96% ± 2.02% and 65.70% ± 2.76% NO level decrease at 10 and 20 μM, respectively [62].

### Other Biological Activities

Other reported biological activities of cembranoids include induction of T lymphocyte proliferation. Three new compounds and a known compound isolated from *Sarcophyton trocheliophorum*, **86–89**, were reported to be active on T lymphocyte cells from mice splenocytes. Compounds **86**, **88**, and **89** significantly induced cluster of differentiation 3 (CD3^+^) T lymphocyte cells proliferation at 3 μM. In addition, **86** increased the CD4^+^/CD8^+^ T lymphocyte cells ratio on mice splenocytes. In contrast, compound **87** exhibited decreased the CD4^+^/CD8^+^ ratio [38]. Other active agents that exhibited activities related to T lymphocyte cell proliferation were two new compounds, **118** and **119**, and also a known compound, **122**, which were obtained from *Sarcophyton mililatensis.* Those compounds showed anti-proliferation activity against Concanavalin A (ConA)-induced T lymphocyte cell proliferation with IC_50_ values of 49.8, 38.9, and 11.4 μM, respectively. Additionally, the three compounds also exerted anti-proliferation activity on LPS-induced B lymphocyte cells, with IC_50_ values of 20.2, 22.1 and 4.9 μM, respectively. In the same report, a known compound, **121**, also exhibited anti-proliferation activity on LPS-induced B lymphocyte cell proliferation, with IC_50_ of 4.8 μM [42].

One study reported that two compounds, **81** (a new compound) and **84** (a known compound), extracted from *Sarcophyton trocheliophorum* showed inhibitory effect towards protein-tyrosine phosphatase 1B (PTP1B), with IC_50_ values of 19.9 and 15.4 μM, respectively [36]. This inhibitory effect is one of interest in the development of type 2 diabetes mellitus treatment as PTP1B is known as a negative regulator of the insulin signaling pathway [95]. Two new compounds, **107** and **110,** isolated from *Sarcophyton glaucum* exhibited anti-larval settlement activity with an adhesive rate of 6.52 and 4.60% at 25 ppm, respectively. In the same study, three other known compounds, **115, 116** and **117**, were shown to have anti-fouling activity against *Balanus amphitrite,* with adhesive rates of 8.19, 14.14, and 7.78% at 25 ppm, respectively [41]. One of the known compounds from Sarcophyton *glaucum*, **139**, possessed neurological activity by competitive inhibition of neuronal glycine with inhibitory constant (K_I_) = 109 μM. It did not have any effect on strychnine toxicity in a mouse experiment model [44].

Compounds **179** and **180** exhibited lethality against brine shrimp *Artemia salina* with lethal ratios of 90.5 and 90.0%, respectively at a concentration of 50 μg/mL [53]. Several of the ten cembranoids (**177, 190, 204–211**) isolated in 2019 were found to possess immunosuppressive activity. Cembranoid **160** showed significant inhibitory effects on the proliferation of LPS induced B lymphocyte cells, with an IC_50_ value of 9.2 µM. **177, 205, 207, 208, 209, 211** possessed immunosuppressive activities through potent inhibition on the proliferation of Con A-induced T lymphocyte cells, with IC_50_ values of 4.5, 8.4, 5.5, 3.9, 2.3, and 6.1 µM, respectively. Compound **210** had considerable specific inhibition on B lymphocyte cell proliferation, with an IC_50_ value of 4.4 µM and selectivity index (SI) of 10.9. This performance was much better than that of the positive control cyclosporin A (CsA) (SI = 3.0). **210** dose-dependently inhibited CD19^+^ B lymphocyte cells proliferation by LPS induction, while it also showed modulatory effects on cytokine production, with the manifestation of decreased IL-6 production and slightly increased IL-10 production. **210** could suppress the derivational expression of CD86 on CD19^+^ B lymphocyte cells upon LPS stimulation. In vitro, LPS addition led to B lymphocyte cell growth and plasma cell formation (from 2.31% to 11.0%) and compound **210** dose-dependently inhibited the plasma cell proliferation [52]. **193** and **194** isolated from Yongxing Island *Sinularia* sp. possessed anti-diabetic activity through mild inhibitory activity against PTP1B with IC_50_ values of 47.5 and 12.5 mM, respectively, measured against sodium orthovanadate as the positive control (IC_50_ 881 μM) [54]. Cembranoids **200** and **202** isolated from Xisha Islands *Sinularia* sp. can inhibit Alzheimer’s amyloid-beta 42 (Aß_42_) aggregation at a concentration of 10 µM, with inhibition of 20.6 and 37.2%, respectively. This potency was comparable to that of the positive control curcumin (20.5%) [60]. Cembranoid **223** showed significant toxicity against *A. salina* with an LD_50_ value of 25 μg/mL [63].

A new cembranoid, **302,** isolated from *Chicoreus ramosus* possessed anti-oxidant activity through 2,2-diphenyl-1-picrylhydrazyl (DPPH) and 2,2′-azino-bis(3-ethylbenzothiazoline-6-sulfonic acid) (ABTS^+^) scavenging activity, with IC_50_ values of 0.26 and 0.36 mg/mL, respectively [78]. Fourteen cembranoids have been isolated from the Caribbean Sea *Eunicea* sp., with some of them possessing anti-diabetic activity. **304**, **312–315** improved INS-1 pancreatic β cell proliferation with a ratio of 1.9, 1.7, 1.7, 2.2, 1.4, and 1.1, respectively compared with control. In this regard, **303** and **305–311** have not been tested for biological activity [79]. The Bornean soft coral *Nephthea* sp. contained **316,** which showed insecticidal activity through repellent activity against maize weevil *Sitophilus zeamais* (grains pest) at 25 μg/cm^2^ [80]. In 2019, Castellanos et al. isolated **319** from *Pseudoplexaura porosa* and **320** from *Eunicea knighti*, which possessed AChE inhibition activity with IC_50_ of 1.40 and 0.358 µM, respectively. These compounds have the potential to be developed for neurodegenerative disease treatment, e.g. Alzheimer’s disease [82]. The known compound, **351**, isolated from *L. flava* possessed immunosuppressive activity through inhibiting the proliferation of ConA-induced T lymphocyte cells and/or LPS-induced B lymphocyte cells in vitro, with IC_50_ of 10.7 and 38.6 μM, respectively [90]. Compound **352** isolated from *S. variolaris* possessed potent anti-oxidant activity through DPPH and ABTS^+^ scavenging activity with IC_50_ values of 1.41 and 1.61 mM, respectively, which were greater than that of the standard agent α-tocopherol (IC_50_ of 1.51 and 1.70 mM, respectively) [91]. Cembrane-type diterpenoid **213** and **216** showed inhibitory effect toward α-Glucosidase with IC_50_ value of 10.65 ± 0.16 and 30.31 ± 1.22 μM, respectively [61]. Furthermore, *Lobophytum* sp. from Xisha Island contained seven compounds namely **270–276** which exhibited a weak inhibitory effect of XBP-Splicing on B16-F10 tumor cells at a concentration of 10 μM [72].

Many compounds reviewed in this paper were found to have no biological activity of interest reported in the respectively published article, such as **174–176** isolated from *S. nanolobata* [51]. The same was true to **184–186** isolated from *S. flexibilis* [94]; **192** isolated from the South China Sea soft coral *Sinularia* sp. [54], **201** and **203** isolated from Xisha Islands *Sinularia* sp. [60], **204** and **206** isolated from Xigu Island *S. scabra* [52], and neither did for **278, 280**, **281** and **282** isolated from the soft coral *Briareum* sp. did not possess any biological activity [74]. Cembranoids **225–229** and **231–233** isolated by Zhao et al. in 2016 did not exhibit the activity of interest [64]. The same goes for three new cembranoids isolated by Zhang et al. [49], namely **265**, **266** and **267** [70] as well as two new cembranoids, **268** and **269**, isolated by Li et al*.* [43] from the Hainan soft coral *Lobophytum* sp. [71]. No biological activity was detected in another new compound, **322**, from *K. flaccidum* [83], as was the case with a new cembranoid, **324,** isolated from *J. fragilis* [84], and with **327** derived from aquacultured *B. violaceum* [85]. Similarly, no biological activities of interest were recorded in the original published papers of the three novel cembranoids (**330**, **331** and **332**) isolated from *J. fragilis* [86], two new cembranoids (**339** and **340**) from *E. caribaeorum* [87], **345** from aquacultured *B. violaceum* [89], two novel cembranoids, **349** and **350,** from *L. flava* that originated from Xisha Islands [90], as well as a novel compound, **355,** isolated from the Taiwanese soft coral *B. excavatum* [92].

Several cembranoid compounds have been recently discovered and have not been thoroughly tested for their biological activities [27, 28, 39]. Rahelivao et al. [59], isolated a new compound, **173**, from the Madagascar soft coral *S. gravis*, but no biological activity was reported [50]. Dongsha Atoll soft corals *L. crassum* contained three novel compounds, **237**, **238** and **239**, which have not yet been explored for their biological activities [65]. **250** isolated from aquacultured *L. crassum* did not possess any biological activity of interest, while **251** has not been thoroughly tested [67]. **286** isolated from the Bornean soft coral *Nephthea* sp. [75] and **298** extracted from *Klyxum flaccidum* [77] have not been tested yet. Lastly, five briaranes were isolated from *Briareum stechei* which cultured in the National Museum of Marine Biology and Aquarium, Pingtung, Taiwan. briarenols W-Z **356–359** were the new reported compound and solenolide A **360** was the only known compound being isolated. Compound **357** and **360** were the only compound which exhibited anti-inflammatory activity by enhancing the release of iNOS and COX-2 [93].

## Conclusions

Soft corals or Alcyonacea are rich potential sources of uniques compounds, particularly cembranoid diterpenes. These compounds have been demonstrated to display a spectrum of pharmacological activities such as anti-tumor, antibacterial and anti-inflammatory. Discoveries are being reported continually in the literature for cembranoid compounds isolated from soft corals as technologies for chemical extraction and characterization of secondary metabolites become more advanced.

**Fig. 12:**
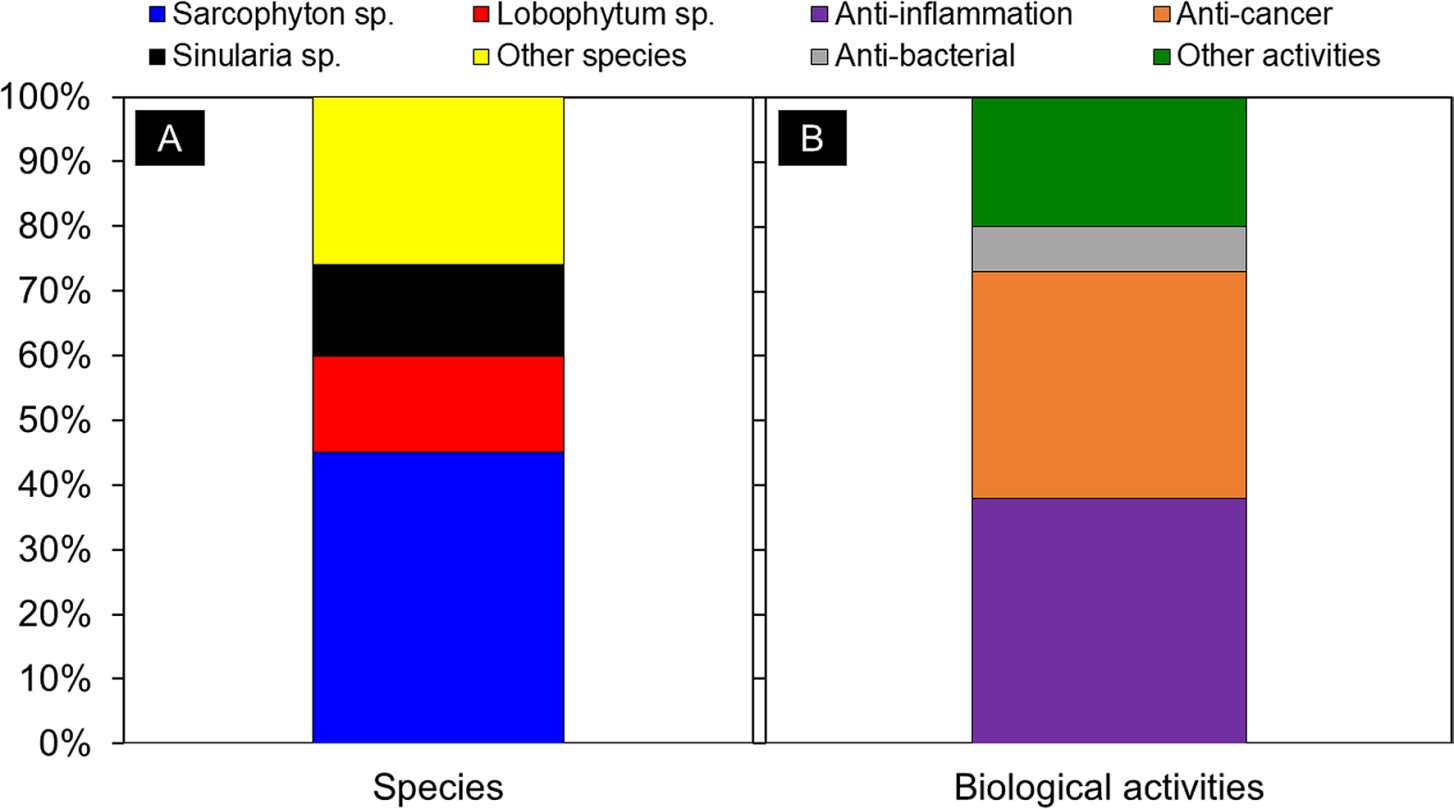
The percentages of cembranoid-producing soft coral species (**a**). The percentage of different biological activities exhibited by cembranoids (**b**)

This review provides an update on recent studies that encompass the isolation of up to 360 cembranoids from marine soft corals and brief accounts of their biological activities reported in the span of the recent five years. Most of the studied compounds were isolated from Sarcophyton sp. (45%), followed by Lobophytum sp. (15%), and Sinularia sp. (14%). Other marine soft corals made up the remaining 26% of species. It is known that cembranoids from marine soft corals possess various biological characteristics. Anti-inflammatory (38%) was found to be the most common biological activity exhibited by cembranoids reported in this review, followed by anti-cancer (35%), and anti-bacterial (7%), whereas other activities encompassed the remaining 20% (Fig. 12). These early findings can lead to more detailed studies for marine cembranoid-based drug discovery and development.

Despite the abundance of unique cembranoids identified, the low quantitiy of isolated compounds may be a big challenge for drug applications' evaluation and development. We consider such approaches like synthesis and biosynthesis studies to be developed for applications of these cembranoids for drug discovery. Furthermore, with the recent advanced technology, various types of specific soft corals are becoming possible in aquaculture. This technology provides more abundant organisms to be extracted and a considerable quantity of molecules to be assessed for in vitro and in vivo study.
